# A Mild, Fast, and Scalable Synthesis of Substituted α-Acyloxy Ketones via Multicomponent Reaction Using a Continuous Flow Approach

**DOI:** 10.3389/fchem.2019.00531

**Published:** 2019-07-30

**Authors:** Carlos Eduardo M. Salvador, Carlos Kleber Z. Andrade

**Affiliations:** Laboratório de Química Metodológica e Orgânica Sintética, Instituto de Química, Universidade de Brasília, Brasília, Brazil

**Keywords:** continuous flow, α-acyloxy ketones, Passerini reaction, microwaves, multicomponent reactions

## Abstract

A continuous flow approach for the synthesis of α-acyloxy ketone derivatives from the corresponding arylglyoxals, isocyanides, and carboxylic acids is described. The target products were obtained in excellent yields in short residence times and with high purities via the first transcription of the microwave-to-flow paradigm to the isocyanide-based Passerini reaction. Furthermore, this methodology allowed a 10-fold scale-up using the same experimental conditions initially established.

## Introduction

Over the last decades, α-acyloxy ketones and their derivatives have become outstanding building blocks in organic synthesis due to their presence in several natural products and active pharmaceutical ingredients (APIs) (Prasad et al., 2018). Moreover, they participate in a wide range of synthetic transformations as starting materials or reactants, which result in important compounds such as cyclobutanes, aminoalcohols, hydroxy ketones, and heterocycles (furans, pyrroles, oxazoles, thiophenes, among others) (Shindo et al., 2007; Prasad et al., 2018).

Several reagents and reaction conditions to synthesize α-acyloxy ketones have been described in the literature (Prasad et al., 2018). However, most of them involve metal catalyzed oxidative coupling or electrophilic halogenations followed by nucleophilic displacement, which suffer from low atom economy and employ high amounts of oxidizing agents (Du et al., 2015; Hunter et al., 2016). Alternatively, functionalized α-acyloxy ketones may be easily accessed in a more sustainable way through the Passerini reaction (Neo et al., 2005, 2011).

**Scheme 1:**
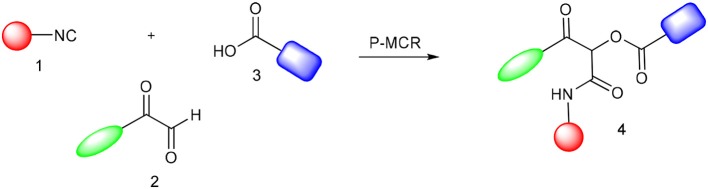
Synthesis of α-acyloxy ketones (4) through Passerini reaction between isocyanides (1), arylglyoxals (2), and carboxylic acids (3).

The Passerini reaction is an efficient and versatile synthetic methodology and belongs to the class of isocyanide-based multicomponent reactions (IMCRs) (Dömling et al., 2012; Kazemizadeh and Ramazani, 2012). This one-pot reaction allows the obtention of α-acyloxy ketones bearing an α-carboxamide moiety from arylglyoxals, aldehydes and carboxylic acids (Scheme 1). In contrast to other processes described in the literature, this reaction not only offers an excellent atom economy without using oxidizing agents but also allows the obtention of functionalized products that can be explored in further transformations. However, the reported reaction times are longer (usually 2 to 3 days) and the Passerini products are obtained in moderate yields and in small scales (Neo et al., 2005, 2011). Compounds **4** are versatile intermediates and can be further converted to β-keto amides by Zn reduction in the presence of NH_4_Cl.

The continuous flow approach has been a relevant strategy in organic synthesis to access a plethora of compounds in a faster and scaled-up manner (Gutmann et al., 2015; Ley et al., 2015; Plutschack et al., 2017), including natural products and APIs (De Souza et al., 2018). Application of this strategy to IMCRs has already been described (Sharma et al., 2013; Heublein et al., 2014; Salvador et al., 2015; Vasconcelos et al., 2017) but its employment in Passerini reactions is still scarce (Sharma et al., 2013).

Herein, we describe a continuous flow approach for the synthesis of α-acyloxy ketone derivatives from the corresponding arylglyoxals, isocyanides and carboxylic acids. The first transcription of the microwave-to-flow paradigm (Glasnov and Kappe, 2011) toward the isocyanide-based Passerini MCR was used to access the target products in excellent yields, short residence times and with high purities through simple evaporation of the solvent at the end (Andrade and Dar, 2016). Furthermore, a 10-fold scale-up was accomplished using the same experimental conditions initially established.

## Results and Discussion

### Batch Microwave Reaction Experiments

To evaluate the most appropriate reaction conditions for the synthesis of α-acyloxy ketones, an initial search for the best solvent was carried out in a batch microwave (MW) preliminary optimization using solvents typically applied in Passerini reactions (Wu et al., 2018) (Table 1) and considering our previous experience in MW-mediated MCR reactions (Barreto et al., 2011a,b, 2014; Salvador et al., 2015; Barreto and Andrade, 2018, 2019). Formation of the Passerini product in toluene at 110°C was not observed probably be due to the low microwave dielectric heating property of this solvent (Gabriel et al., 1998; Kappe et al., 2012) (entry 1). In CHCl_3_ at 60°C, low conversions were observed (entry 2). However, using MeCN at this same temperature, a good conversion of the desired product was achieved (entry 3). In all cases, the applied experimental conditions were the same using equimolar amounts of the starting materials (0.5 mmol), 2 mL of solvent and reaction time of 30 min. Thus, MeCN was chosen as the best solvent for this reaction. Then, we decided to move forward and raise the temperature near the boiling point of MeCN (80°C). To our delight, at this temperature full conversion to product **4a** was achieved (entry 4). By decreasing the reaction time (entries 5–7), we found out that only 5 min were required for a full conversion of phenylglyoxal **2a** to product **4a**, which was isolated essentially pure in 94% yield (entry 7).

**Table 1:** Batch optimization of substituted α-acyloxy ketones synthesis using microwave heating^a^.

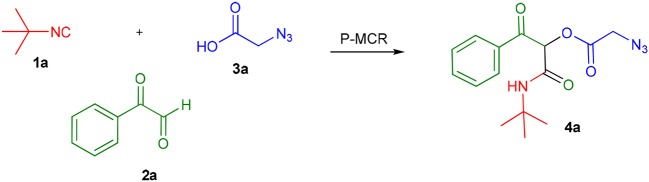				
**Entry**	**Solvent**	**T (°C)**	**Residence time (min)**	**Conversion (°C)**^**b**^
1	Toluene	110	30	–^c^
2	CHCl_3_	60	30	12
3	MeCN	60	30	70
4	MeCN	80	30	>99
5	MeCN	80	20	>99
6	MeCN	80	10	>99
7	MeCN	80	5	>99 (94)^d^

a *Conditions: reactions were carried out using equimolar amounts (0.5 mmol) of isocyanide **1a**, phenylglyoxal **2a** and azidoacetic acid **3a** in 2 mL of solvent*.

b *Determined based on phenylglyoxal **2a** and α-acyloxy ketone **4a** as HPLC-UV/VIS peak area percent at 215 nm*.

c *No product was observed*.

d *Isolated yield in parentheses*.

### Converting Batch Microwave to Continuous Flow System

**Scheme 2:**
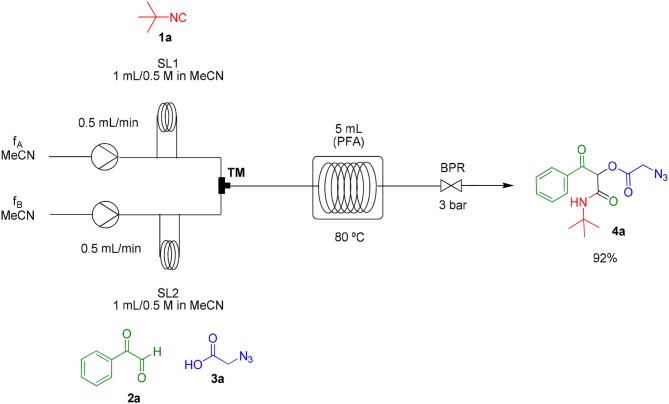
Continuous flow synthesis of α-acyloxy ketone **4a**. Conditions: Feed A (1.0 mL of a 0.5 M solution of **1a** in MeCN) and feed B (1.0 mL of a 0.5 M solution of **2a** and **3a** in MeCN) pumped through a 5 mL PFA reactor at 80°C and 3 bar back-pressure regulator.

As already known, organic reactions performed in short times and well-established temperatures at microwave reactors are prone to be applied to a continuous flow setup device due to the effective residence time control and high thermal transfer achieved in this method (Damn et al., 2010; Kappe et al., 2013). With this in mind, we decided to transpose the microwave batch procedure just established for the synthesis of α-acyloxy ketones **4a** (Table 1, entry 7) into a continuous flow process (Scheme 2). Thus, a 0.5 M solution of *tert*-butyl isocyanide **1a** in MeCN (1 mL) in an injection loop (SL1) was mixed via a syringe pump in a flow rate of 0.5 mL min^−1^ (feed A in MeCN) with a 0.5 M solution of phenylglyoxal **2a** and azidoacetic acid **3a** (in 1 mL), which was pumped simultaneously from the second injection loop (SL2) at the same flow rate (feed B in MeCN). The combined solution possesses the same molar concentration (0.25 M) used in the microwave batch optimization and was pumped through a 5 mL perfluoroalkoxy (PFA) reactor coil (heated at 80°C in an oil bath) with outside (OD) and inner (ID) diameters of 1.6 and 0.8 mm, respectively. Additionally, a 3 bar back pressure regulator (BPR) was used to guarantee greater precision in the follow-up of the residence time. The design of the reactor capacity (5 mL) and the resulting flow rate (1 mL min^−1^) furnished a total residence time unit (RTU) of 5 min (same reaction time used in the microwave optimization process). Satisfyingly, a similar and reproducible yield of compound **4a** was obtained with the continuous flow system as compared to the microwave-assisted synthesis. For comparison, the same reaction was carried out at room temperature and at reflux condition. The first condition gave the product in only 68% yield after 7 h (~10% of non-reacted glyoxal). The second one gave the product in 94% yield but took 4 h to get to completion. Similar results were obtained for arylglyoxal **4c** bearing a strong electron donating group (*p*-OMe). These results show a significant improvement in the reaction outcome using the flow conditions developed in this study.

Inspired by these promising results, we decided to broaden the scope of this methodology and use this flow approach to synthesize various α-acyloxy ketones **4** using isocyanides **1a** and **1b**, arylglyoxals **2a-c**, and carboxylic acids **3a-g**. Numerous α-acyloxy ketones were obtained in excellent yields without the need for any re-optimization of the flowing experimental conditions (Table 2). All compounds from Table 2 were isolated with high purity by a simple evaporation of the solvent (see NMR spectra in the Supplementary Material).

**Table 2:** Scope for the Passerini-MCR flow synthesis of substituted α-acyloxy ketones.

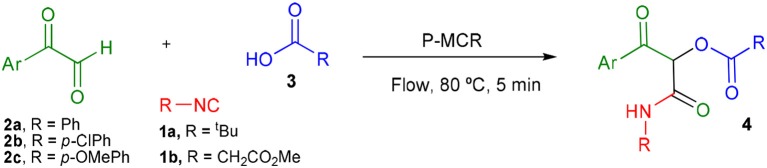					
**Entry**	**RNC**	**Glyoxal**	**Acid**	**α-acyloxy ketone**	**Yield (%)**
1	**1a**	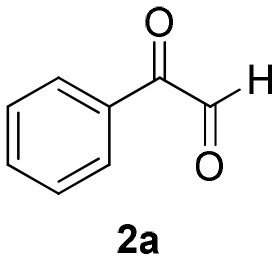	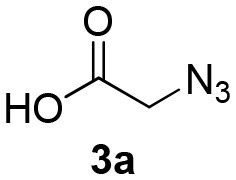	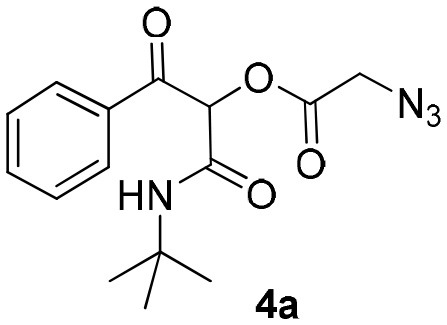	92
2	**1a**	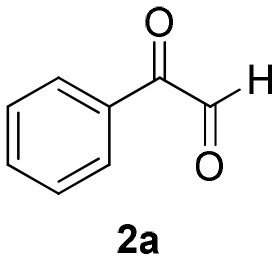	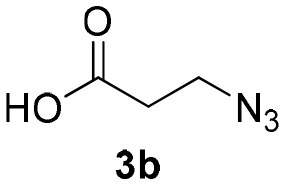	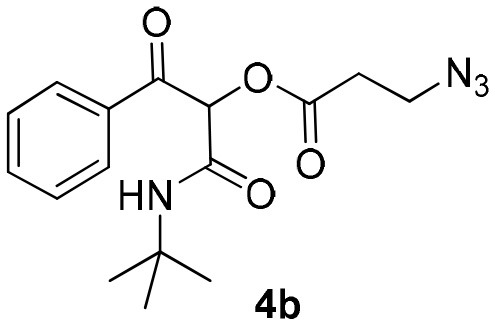	97
3	**1a**	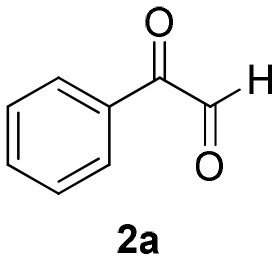	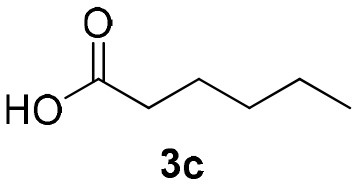	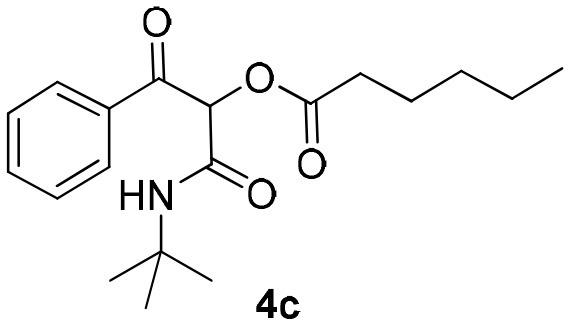	93
4	**1a**	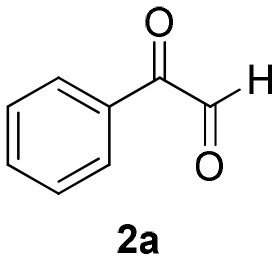	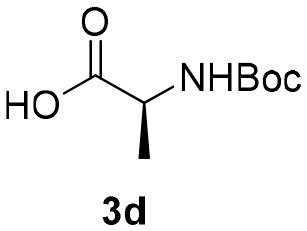	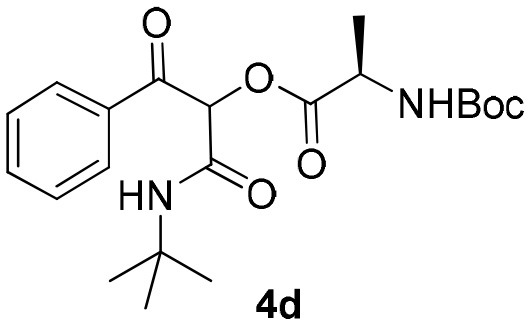	89
5	**1a**	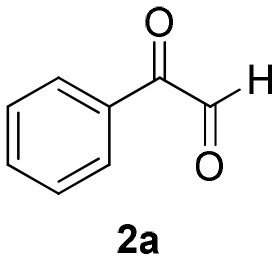	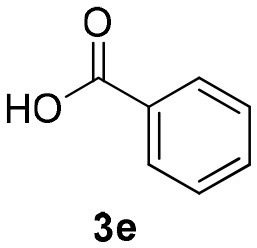	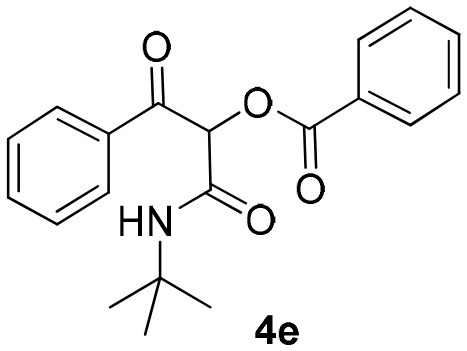	94
6	**1a**	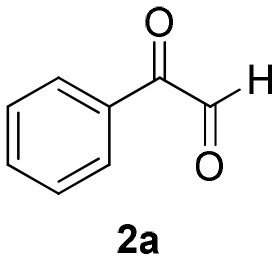	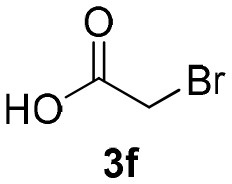	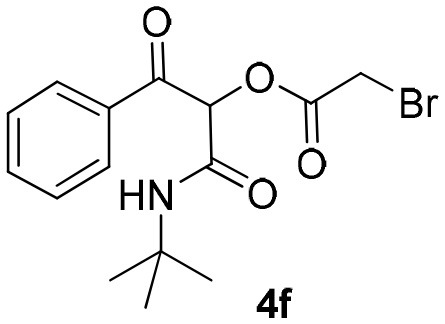	92
7	**1a**	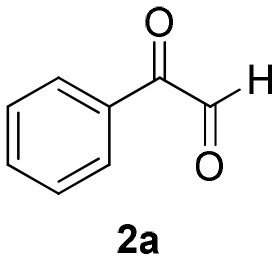	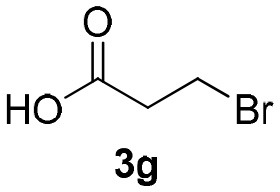	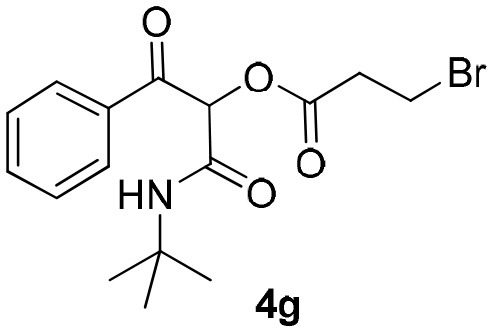	96
8	**1a**	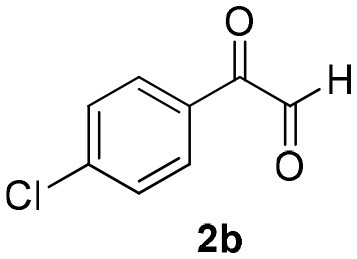	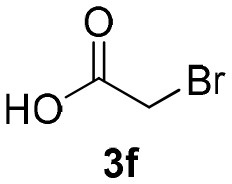	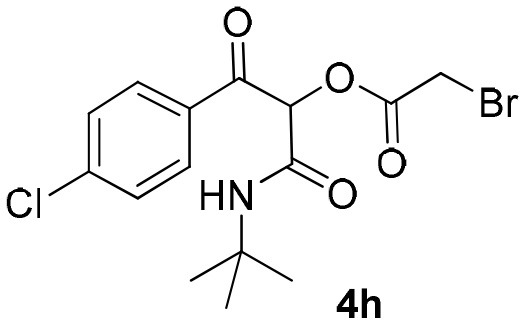	95
9	**1a**	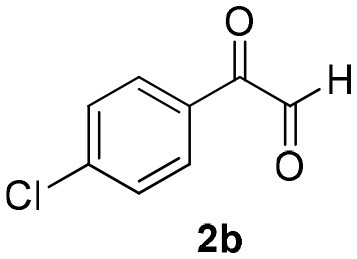	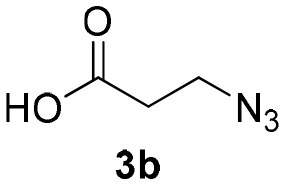	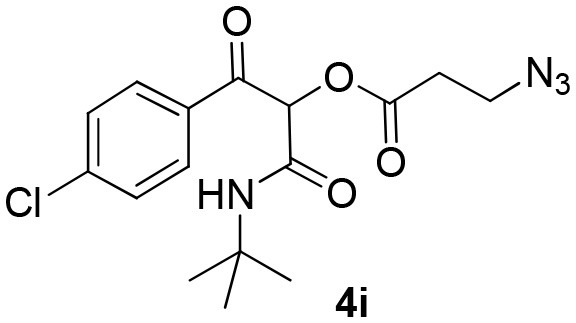	91
10	**1a**	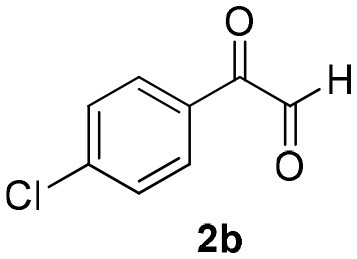	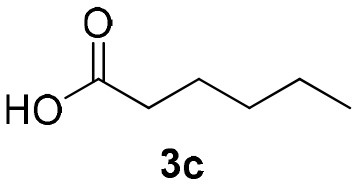	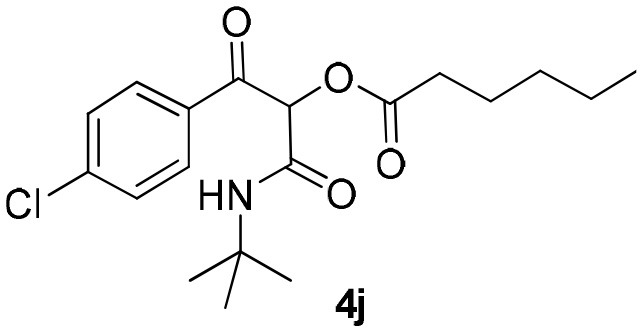	95
11	**1a**	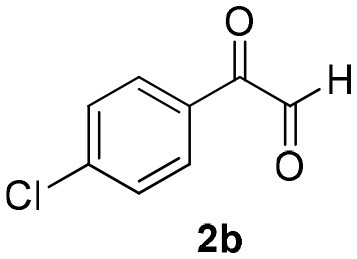	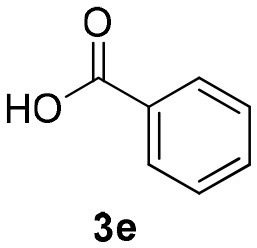	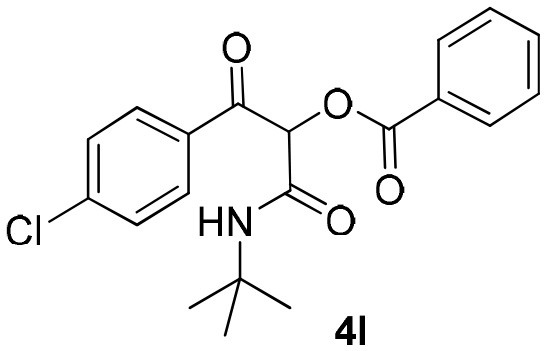	94
12	**1b**	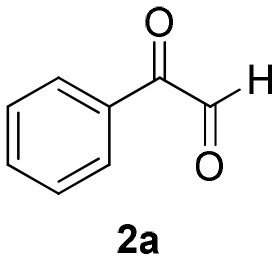	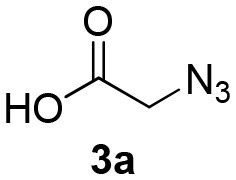	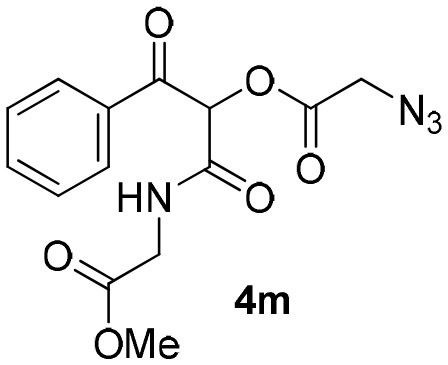	89
13	**1b**	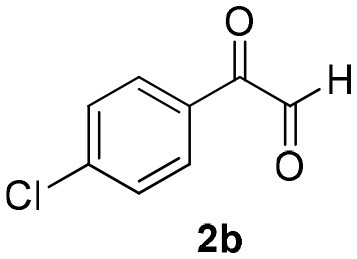	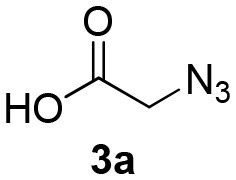	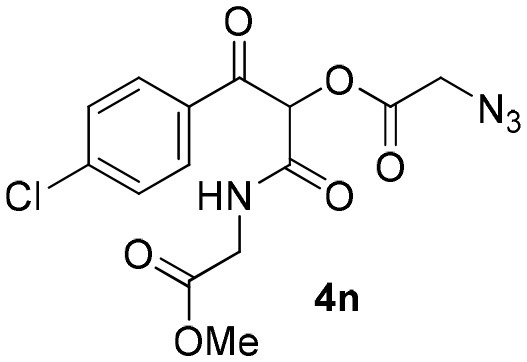	90
14	**1a**	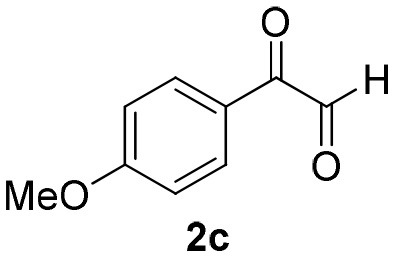	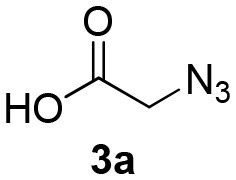	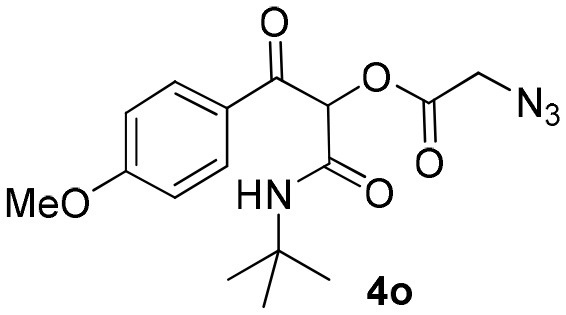	93

### Scale-Up Process

**Scheme 3:**
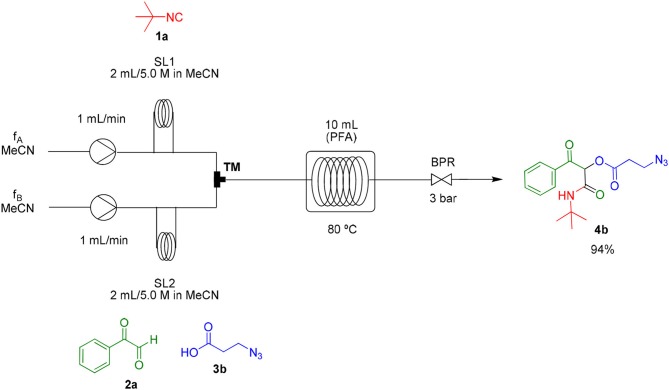
Continuous flow scale-up for the synthesis of α-acyloxy ketone **4b**.

Most organic reactions performed in traditional glassware usually work well at small-scale. Nevertheless, when an increase in scale is required, for instance for a chain development of distinct processes in pharmaceutical and fine chemicals, a great challenge arises in most cases due to inherent problems of scaling up processes such as heat generated and transferred, potential hazards of chemical compounds and environmental issues (Geyer et al., 2006). In contrast, the scale-up reaction performed in flow technology work very well due to the use of versatile approaches, which have similarities with the conditions used in small scale reactions (May et al., 2012, 2016). According to the features of flow processes, the scale-up synthesis of compound **4b** was used as a proof of concept and was achieved by increasing the length of the injection loop (2 mL) for better solubility of the starting materials and the length of the reactor coil (10 mL) to keep the same RTU used before (5 min). With these small adjustments, it was possible to achieve a reproducible 10-fold increase in the scale for the synthesis of compound **4b** in a productivity of 0.312 g/min (Scheme 3).

## Conclusion

In summary, we have applied the first transcription of the microwave-to-flow paradigm for the isocyanide-based Passerini reaction to the synthesis of substituted α-acyloxy ketones via a continuous flow process. The target products can be further functionalized and were easily obtained in low residence times and excellent yields without the need for purification, which constitutes a significant improvement over the existing methods for preparing these compounds. Furthermore, the success of this methodology was corroborated by a 10-fold increase in the scale under similar reaction conditions leading to a productivity of 0.312 g/min.

## Experimental Part

### General Remarks

^1^H-NMR spectra were recorded on a Varian Mercury Plus 300 MHz instrument. ^13^C-NMR spectra were recorded on this same instrument at 75 MHz. Chemical shifts (δ) are expressed in ppm downfield from TMS as internal standard. The letters s, brs, t, dd and m are used to indicate singlet, broad singlet, triplet, double of doublets, and multiplet, respectively. HRMS experiments were performed on a TOF LC/MS instrument equipped with an ESI ion source (positive ionization mode) from Bruker. Analytical HPLC (Shimadzu LC20) analysis was carried out on a C18 reversed-phase (RP) analytical column (150 × 4.6 mm, particle size 5 μm) at 37°C using a mobile phase A (water/acetonitrile 90:10 (v/v) + 0.1% TFA) and B (MeCN + 0.1% TFA) at a flow rate of 1.5 mL/min. The following gradient was applied: linear increase from 30 to 100% B in 17 min. All microwave-mediated reactions were carried out in a CEM, Co. Discover microwave reactor in borosilicate closed glass reaction vessels (10 mL). For continuous flow procedures, KDS 210 Legacy dual syringe pump was used. Static backpressure, modular mixers, sample manual injectors, fittings, and tubings are commercially available from Idex Health & Science, Inc. All solvents and chemicals were obtained from standard commercial suppliers and were used without any further purification.

### Starting Materials

2-azidoacetic acid (Yu et al., 2011), 3-azidopropionic acid (Yang et al., 2013), 2-oxo-2-phenylacetaldehyde, 2-(4-chlorophenyl)-2-oxoacetaldehyde and 2-(4-methoxyphenyl)-2-oxoacetaldehyde (Wang et al., 2013) were prepared and characterized according to literature procedures.

### General Procedure for Batch Optimization of α-Acyloxy Ketones Synthesis Using Microwave Heating (Table 1)

A sealed 10 mL borosilicate glass reaction vessel, equipped with a magnetic stir bar and containing a mixture of equimolar amounts (0.5 mmol) of isocyanide **1a**, phenylglyoxal **2a**, and azidoacetic acid **3a** in 2 mL of MeCN was irradiated in a CEM Co. Discover reactor for 5 min at 80°C. After cooling to room temperature, the reaction mixture was concentrated under reduced pressure and the desired product (**4a**) was isolated in essentially pure form (entry 7).

### General Procedure for the Flow Synthesis of α-Acyloxy Ketones 4a to 4o

The PFA reactor coil (0.8 mm inner diameter; 5.0 mL) was completely filled with solvent (MeCN) and then heated in an oil bath at 80°C. Isocyanide **1a** (0.5 mmol) was diluted in 1.0 mL of MeCN (0.5 M) and stored in a 1 mL sample loop (SL1). Phenylglyoxal **2a** and azidoacetic acid **3a** (0.5 mmol each) were diluted in 1.0 mL of MeCN (0.5 M) and stored in a 1 mL sample loop (SL2). The sample loops were connected via a 6-way manual valve and were at the same time pumped into a T-shaped mixer (TM). After mixing, the resulting solution was fed (1 mL.min^−1^ stream) into the PFA reactor coil, thus remaining for 5 min of residence time under heating (80°C). A static back pressure regulator (3 bar) was used to allow the best fluid flow condition (Kappe et al., 2013). The desired products (**4a−4o**) were obtained after solvent evaporation under reduced pressure.

### General Procedure for Continuous Flow Scale-Up for Synthesis of α-Acyloxy Ketone 4b

Same as above using 5.0 M solutions of compounds **1a**, **2a**, and **3b**, a 10.0 mL PFA reactor coil and 2 mL sample loops. Compound **4b** was obtained in 94% yield (1.56 g) with a productivity of 0.312 g/min (see Scheme 3).

### 1-(*Tert*-Butylamino)-1,3-Dioxo-3-Phenylpropan-2-yl-2-Azidoacetate (4a)

Very viscous yellow oil (0.146 g, 92% yield); HRMS (ESI): m/z: calcd for C_15_H_18_N_4_O_4_Na [M + Na^+^]: 341.1220, found: 341.1226. ^1^H-NMR (300 MHz, CDCl_3_) δ (ppm) 8.17–8.10 (m, 2H), 7.67–7.58 (m, 1H), 7.54–7.46 (m, 2H), 6.30 (s, 1H), 6.14 (brs, 1H), 4.08 (s, 2H), 1.33 (s, 9H). ^13^C-NMR (75 MHz, CDCl_3_) δ (ppm) 191.3, 166.3, 161.7, 134.3, 134.1, 129.7, 128.7, 77.5, 52.2, 50.1, 28.4.

### 1-(*Tert*-Butylamino)-1,3-Dioxo-3-Phenylpropan-2-yl-3-Azidopropanoate (4b)

Very viscous yellow oil (0.161 g, 97% yield); HRMS (ESI): m/z: calcd for C_16_H_20_N_4_O_4_Na [M + Na^+^]: 355.1377, found: 355.1387. ^1^H-NMR (300 MHz, CDCl_3_) δ (ppm) 8.15–8.10 (m, 2H), 7.64–7.58 (m, 1H), 7.53–7.47 (m, 2H), 6.26 (s, 1H), 6.22 (brs, 1H), 3.61 (*t, J* = 6.2 Hz, 2H), 2.75 (*t, J* = 6.2 Hz, 2H), 1.34 (s, 9H). ^13^C-NMR (75 MHz, CDCl_3_) δ (ppm) 191.8, 168.8, 162.3, 134.3, 134.1, 129.6, 128.6, 76.5, 52.0, 46.4, 33.6, 28.4.

### 1-(*Tert*-Butylamino)-1,3-Dioxo-3-Phenylpropan-2-yl-Hexanoate (4c)

Very viscous colorless oil (0.155 g, 93% yield); HRMS (ESI): m/z: calcd for C_19_H_27_NO_4_Na [M + Na^+^]: 356.1832, found: 356.1829; ^1^H-NMR (300 MHz, CDCl_3_) δ (ppm) 8.18–8.08 (m, 2H), 7.65–7.55 (m, 1H), 7.54–7.43 (m, 2H), 6.21 (s, 1H), 6.21–6.17 (brs, 1H), 2.54–2.44 (m, 2H), 1.74–1.59 (m, *2*H), 1.36–1.31 (m, 13H), 0.95–0.85 (m, 3H). ^13^C-NMR (75 MHz, CDCl_3_) δ (ppm) 192.5, 171.6, 162.8, 134.5, 134.0, 129.6, 128.5, 76.1, 51.9, 33.6, 31.1, 28.5, 24.3, 22.2, 13.8.

### 1-(*Tert*-Butylamino)-1,3-Dioxo-3-Phenylpropan-2-yl-(*Tert*-Butoxycarbonyl)-D-Alaninate (4d)

Very viscous brown oil (0.181 g, 89% yield); HRMS (ESI): m/z: calcd for C_21_H_30_N_2_O_6_Na [M + Na^+^]: 429.1996, found: 429.1998; Mixture of diastereomers: ^1^H-NMR (300 MHz, CDCl_3_) δ (ppm) 8.11–8.09 (m, 4H), 7.65–7.57 (m, 2H), 7.53–74.6 (m, 4H), 6.48 (brs, 1H), 6.35 (brs, 1H), 6.31 (s, 1H), 6.23 (s, 1H), 4.54–4.37 (m, 2H), 1.48–1.43 (m, 24H), 1.35 (*d, J* = 2.0 Hz, 18H). ^13^C-NMR (75 MHz, CDCl_3_) δ (ppm) 191.7, 191.5, 171.8, 170.9, 162.6, 162.6, 134.6, 134.4, 134.1, 134.0, 129.7, 129.7, 128.6, 128.6, 80.3, 76.4, 52.0, 49.1, 28.5, 28.5, 28.3, 28.5, 17.8, 17.7.

### 1-(*Tert*-Butylamino)-1,3-Dioxo-3-Phenylpropan-2-yl Benzoate (4e)

Very viscous yellow oil (0.170 g, 94% yield); HRMS (ESI): m/z: calcd for C_20_H_21_NO_4_Na [M + Na^+^]: 362.1363, found: 362.1365; ^1^H-NMR (300 MHz, CDCl_3_) δ (ppm) 8.24–8.04 (m, 2H), 8.15–8.01 (m, 2H), 7.67–7.57 (m, 2H), 7.55–7.46 (m, 4H), 6.43 (s, 1H), 6.28 (brs, 1H), 1.39 (s, 9H). ^13^C-NMR (75 MHz, CDCl_3_) δ (ppm) 192.4, 164.6, 162.9, 134.6, 134.1, 133.9, 133.4, 129.7, 129.7, 128.7, 128.6, 76.8, 52.0, 28.6.

### 1-(*Tert*-Butylamino)-1,3-Dioxo-3-Phenylpropan-2-yl-2-Bromoacetate (4f)

Very viscous yellow oil (0.164 g, 92% yield); HRMS (ESI): m/z: calcd for C_15_H_18_BrNO_4_Na [M + Na^+^]: 380.0296, found: 380.0296; ^1^H-NMR (300 MHz, CDCl_3_) δ (ppm) 8.13–8.10 (m, 2H), 7.66–7.59 (m, 2H), 7.55–7.47 (m, 2H), 6.27 (s, 1H), 6.26 (brs, 1H), 4.09–3.86 (m, 2H), 1.35 (s, 9H). ^13^C-NMR (75 MHz, CDCl_3_) δ (ppm) 191.9, 165.0, 162.0, 134.3, 134.2, 129.8, 128.6, 77.1, 52.1, 28.5, 24.8.

### 1-(*Tert*-Butylamino)-1,3-Dioxo-3-Phenylpropan-2-yl-3-Bromopropanoate (4g)

Very viscous colorless oil (0.178 g, 96% yield); HRMS (ESI): m/z: calcd for C_16_H_20_BrNO_4_Na [M + Na^+^]: 392.0468, found: 392.0473; ^1^H-NMR (300 MHz, CDCl_3_) δ (ppm) 8.15–8.10 (m, 2H), 7.66–7.57 (m, 1H), 7.54–7.45 (m, 2H), 6.27 (s, 1H), 6.25 (brs, 1H), 3.61 (*t, J* = 6.6 Hz, 2H), 3.14 (*t, J* = 6.6 Hz, 2H), 1.34 (s, 9H). ^13^C-NMR (75 MHz, CDCl_3_) δ (ppm) 191.8, 168.5, 162.3, 134.4, 134.2, 129.7, 128.6, 76.5, 52.1, 37.2, 28.5, 25.1.

### 1-(*Tert*-Butylamino)-3-(4-Chlorophenyl)-1,3-Dioxopropan-2-yl-2-Bromoacetate (4h)

Very viscous colorless oil (0.186 g, 95% yield); HRMS (ESI): m/z: calcd for C_15_H_17_BrClNO_4_Na [M + Na^+^]: 411.9922, found: 411.9918; ^1^H-NMR (300 MHz, CDCl_3_) δ (ppm) 8.12–8.06 (m, 8H), 7.51–7.44 (m, 8H), 6.24 (brs, 1H), 6.21 (s, 1H), 3.99 (m, 2H), 1.35 (s, 9H). ^13^C-NMR (75 MHz, CDCl_3_) δ (ppm) 190.0, 164.9, 161.8, 141.0, 132.6, 131.2, 129.3, 129.0, 128.8, 76.9, 52.2, 28.5, 24.7.

### 1-(*Tert*-Butylamino)-3-(4-Chlorophenyl)-1,3-Dioxopropan-2-yl-3-Azidopropanoate (4i)

Very viscous yellow oil (0.175 g, 91% yield); HRMS (ESI): m/z: calcd for C_16_H_19_N_4_O_4_Na [M + Na^+^]: 389.0987, found: 389.1002; ^1^H-NMR (300 MHz, CDCl_3_) δ (ppm) 8.12–8.06 (m, 2H), 7.51–7.44 (m, 2H), 6.25 (brs, 1H), 6.20 (s, 1H), 3.64 (*t, J* = 6.2 Hz, 2H), 2.78 (*t, J* = 6.2 Hz, 2H), 1.34 (s, 9H). ^13^C-NMR (75 MHz, CDCl_3_) δ (ppm) 190.7, 168.8, 162.1, 140.8, 132.7, 131.6, 129.0, 76.4, 52.1, 46.5, 33.6, 28.5.

### 1-(*Tert*-Butylamino)-3-(4-Chlorophenyl)-1,3-Dioxopropan-2-yl-Hexanoate (4j)

Very viscous yellow oil (0.175 g, 95% yield); HRMS (ESI): m/z: calcd for C_19_H_26_ClNO_4_Na [M + Na^+^]: 390.1443, found: 390.1446; ^1^H-NMR (300 MHz, CDCl_3_) δ (ppm) 8.11–8.06 (m, 2H), 7.49–7.44 (m, 2H), 6.19 (brs, 1H), 6.14 (s, 1H), 2.54–2.45 (m, 2H), 1.73–1.59 (m, *2*H), 1.36–1.31 (m, 13H), 0.95–0.85 (m, 3H).^13^C-NMR (75 MHz, CDCl_3_) δ (ppm) 191.3, 171.5, 162.6, 140.5, 132.9, 131.1, 128.8, 75.9, 51.9, 33.6, 31.0, 28.4, 24.3, 22.2, 13.8.

### 1-(*Tert*-Butylamino)-3-(4-Chlorophenyl)-1,3-Dioxopropan-2-yl-Benzoate (4l)

Very viscous brown oil (0.176 g, 94% yield); HRMS (ESI): m/z: calcd for C_20_H_20_ClNO_4_Na [M + Na^+^]: 396.0973, found: 396.0966; ^1^H-NMR (300 MHz, CDCl_3_) δ (ppm) 8.18–8.11 (m, 2H), 8.09–8.04 (m, 2H), 7.68–7.60 (m, 1H), 7.55–7.46 (m, 4H), 6.36 (s, 1H), 6.29 (bs, 1H), 1.39 (s, 9H). ^13^C-NMR (75 MHz, CDCl_3_) δ (ppm) 191.2, 164.5, 162.7, 140.7, 134.0, 132.9, 131.1, 129.8, 129.0, 128.7, 76.7, 52.1, 28.5.

### 1-((2-Methoxy-2-Oxoethyl)Amino)-1,3-Dioxo-3-Phenylpropan-2-yl-2-Azidoacetate (4m)

Very viscous colorless oil (0.151 g, 89% yield); HRMS (ESI): m/z: calcd for C_14_H_14_N_4_O_6_ [M + H^+^]: 341.1220, found: 341.1226. ^1^H-NMR (300 MHz, CDCl_3_) δ (ppm) 8.14–8.10 (m, 2H), 7.66–7.61 (m, 1H), 7.54–7.47 (m, 2H), 6.47 (s, 1H), 6.47 (brs, 1H), 4.16–4.08 (dd, *J* = 18.0, 6.0 Hz, 1H), 4.12 (s, 2H), 3.99–3.91 (dd, *J* = 18.0, 6.0 Hz, 1H), 3.73 (s, 3H). ^13^C NMR (75 MHz, CDCl_3_) δ 190.3, 169.4, 166.5, 163.2, 134.5, 133.8, 129.8, 128.7, 76.7, 52.6, 50.0, 41.1.

### 1-(4-Chlorophenyl)-3-((2-Methoxy-2-Oxoethyl)Amino)-1,3-Dioxopropan-2-yl-2-Azidoacetate (4n)

Very viscous yellow oil (0.168 g, 90% yield); HRMS (ESI): m/z: calcd for C_14_H_13_N_4_O_6_Cl [M + H^+^]: 369.0596, found: 369.0604. ^1^H-NMR (300 MHz, CDCl_3_) δ (ppm) 8.09–8.05 (m, 2H), 7.50–7.46 (m, 1H), 7.54–7.47 (m, 2H), 7.13 (brs, 1H), 6.42 (s, 1H), 4.16–4,08 (dd, *J* = 18.0, 6.0 Hz, 1H), 4.14 (s, 2H), 4.02–3.96 (dd, *J* = 18.0, 6.0 Hz, 1H), 3.95 (s, 3H). ^13^C-NMR (75 MHz, CDCl_3_) δ (ppm) 189.1, 169.4, 166.4, 163.0, 141.3, 132.1, 131.2, 129.1, 76.7, 52.7, 50.0, 41.1.

### 1-(*Tert*-Butylamino)-3-(4-Methoxyphenyl)-1,3-Dioxopropan-2-yl-2-Azidoacetate (4o)

Very viscous brown oil (0.162 g, 93% yield); HRMS (ESI): m/z: calcd for C_16_H_20_N_4_O_5_ [M + Na^+^]: 371.1326, found: 371.1332. ^1^H-NMR (300 MHz, CDCl_3_) δ (ppm) 8.15–8.12 (m, 2H), 6.98–6.96 (m, 2H), 6.24 (brs, 1H), 6.12 (s, 1H), 4.10 (s, 2H), 3.90 (s, 2H), 1.33 (s, 9H). ^13^C NMR (75 MHz, CDCl_3_) δ (ppm) 189.3, 166.3, 164.6, 162.1, 132.3, 126.9, 113.9, 77.5, 55.5, 52.2, 50.1, 28.5.

## Data Availability

All datasets generated for this study are included in the manuscript and/or the Supplementary Files.

## Author Contributions

CS was responsible for designing and performing the experiments and helped writing the publication. CA directed the project and wrote the publication.

### Conflict of Interest Statement

The authors declare that the research was conducted in the absence of any commercial or financial relationships that could be construed as a potential conflict of interest.
