# Supplementary material for: A Mild, Fast, and Scalable Synthesis of Substituted α-Acyloxy Ketones via Multicomponent Reaction Using a Continuous Flow Approach
Source: Front Chem. 2019 Jul 30;7:531. doi: 10.3389/fchem.2019.00531 (PMC6690000; doi:10.3389/fchem.2019.00531)

Supplementary Material

A mild, fast and scalable synthesis of substituted α-acyloxy ketones via multicomponent reaction using a continuous flow approach

Carlos Eduardo M. Salvador, Carlos Kleber Z. Andrade[*].

Laboratório de Química Metodológica e Orgânica Sintética, Instituto de Química, Universidade de Brasília, Campus Universitário Darcy Ribeiro, 70904-970, Brasília-DF, Brazil

*** Correspondence:**
Corresponding Author
ckleber@unb.br

Table of Contents

Flow Setup S1

NMR spectra S2

Mass Spectra S3

S1 - Flow Setup

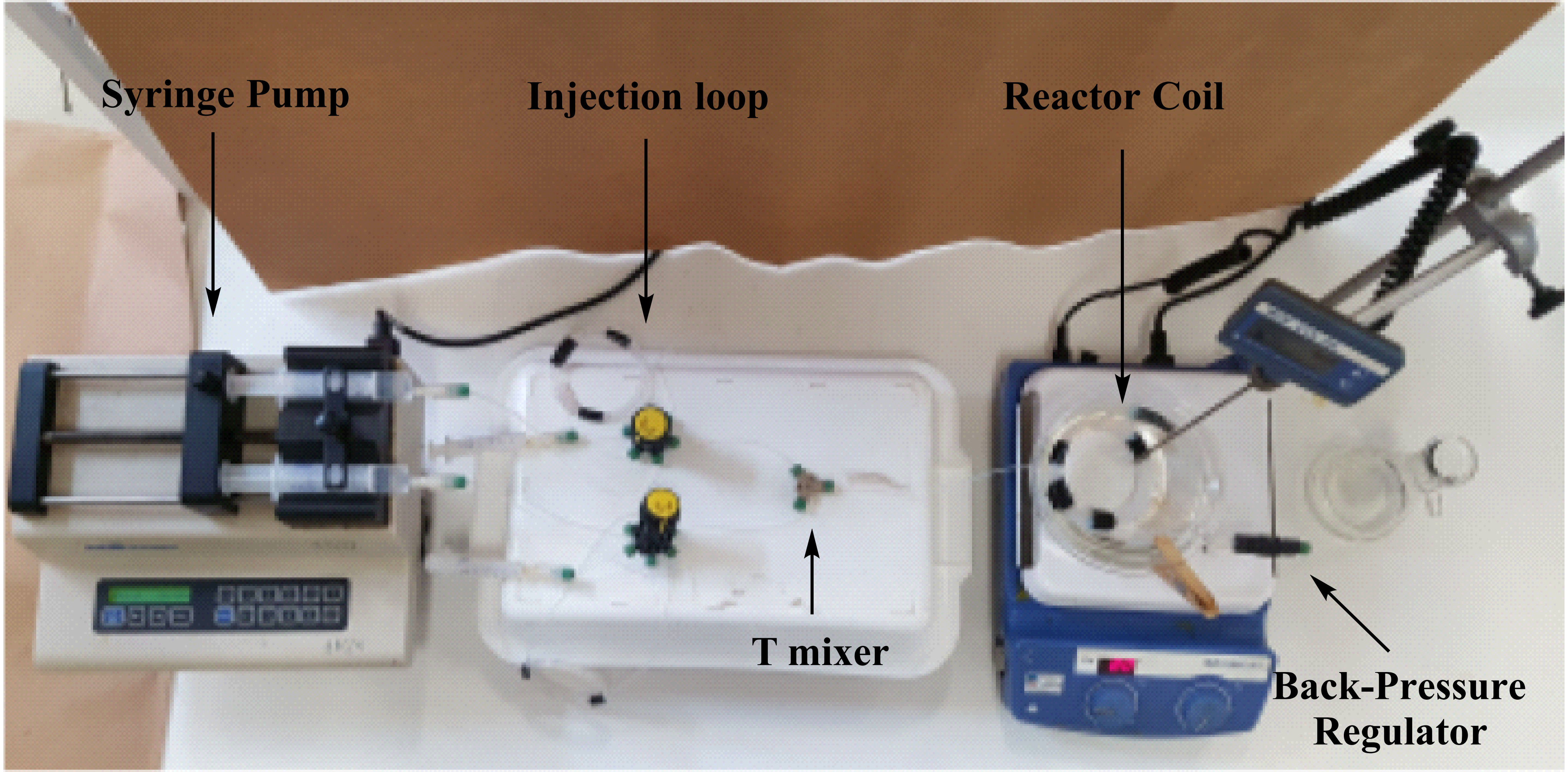

NMR spectra - S2

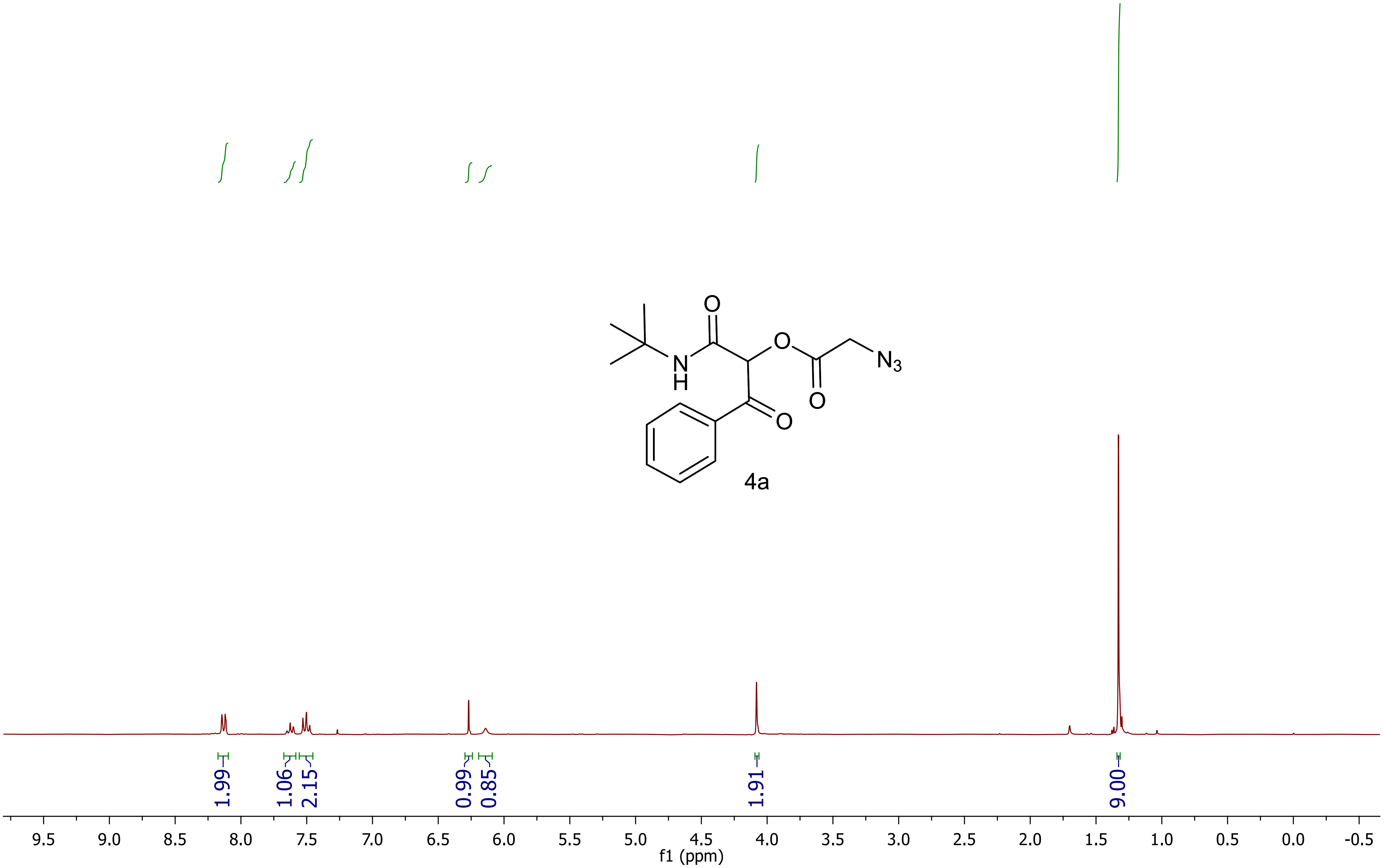

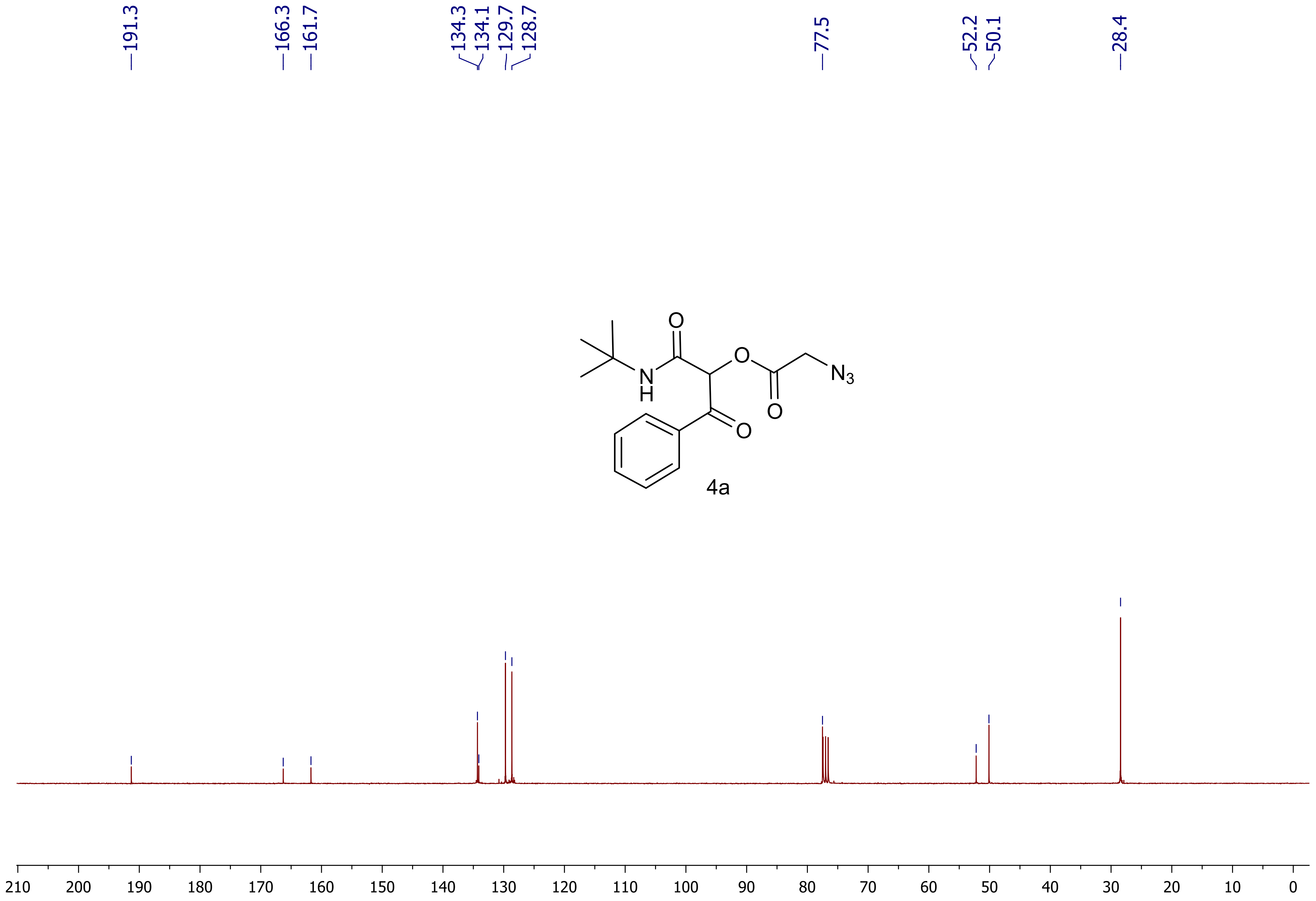
191.3
166.3
161.7
134.3
134.1
129.7
128.7
77.5
52.2
50.1
28.4
O
O
O
O
N
H
N3
4a
210 200 190 180 170 160 150 140 130 120 110 100 90 80 70 60 50 40 30 20 10 0

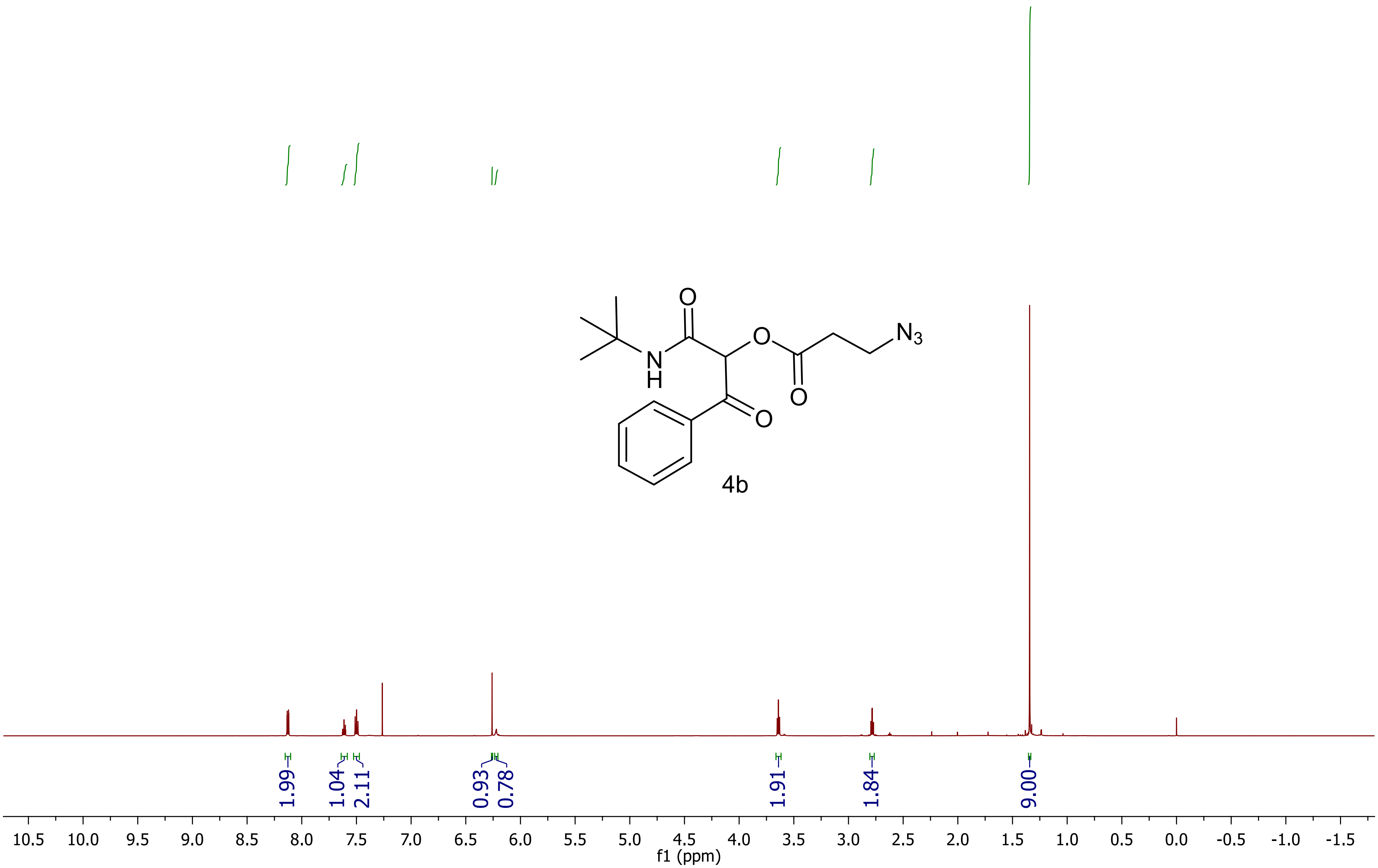

O
N
H
O
N3
O
O
4b
1.99
1.04
2.11
0.93
0.78
1.91
1.84
9.00
10.5 10.0 9.5 9.0 8.5 8.0 7.5 7.0 6.5 6.0 5.5 5.0 4.5 4.0 3.5 3.0 2.5 2.0 1.5 1.0 0.5 0.0 -0.5 -1.0 -1.5
f1 (ppm)

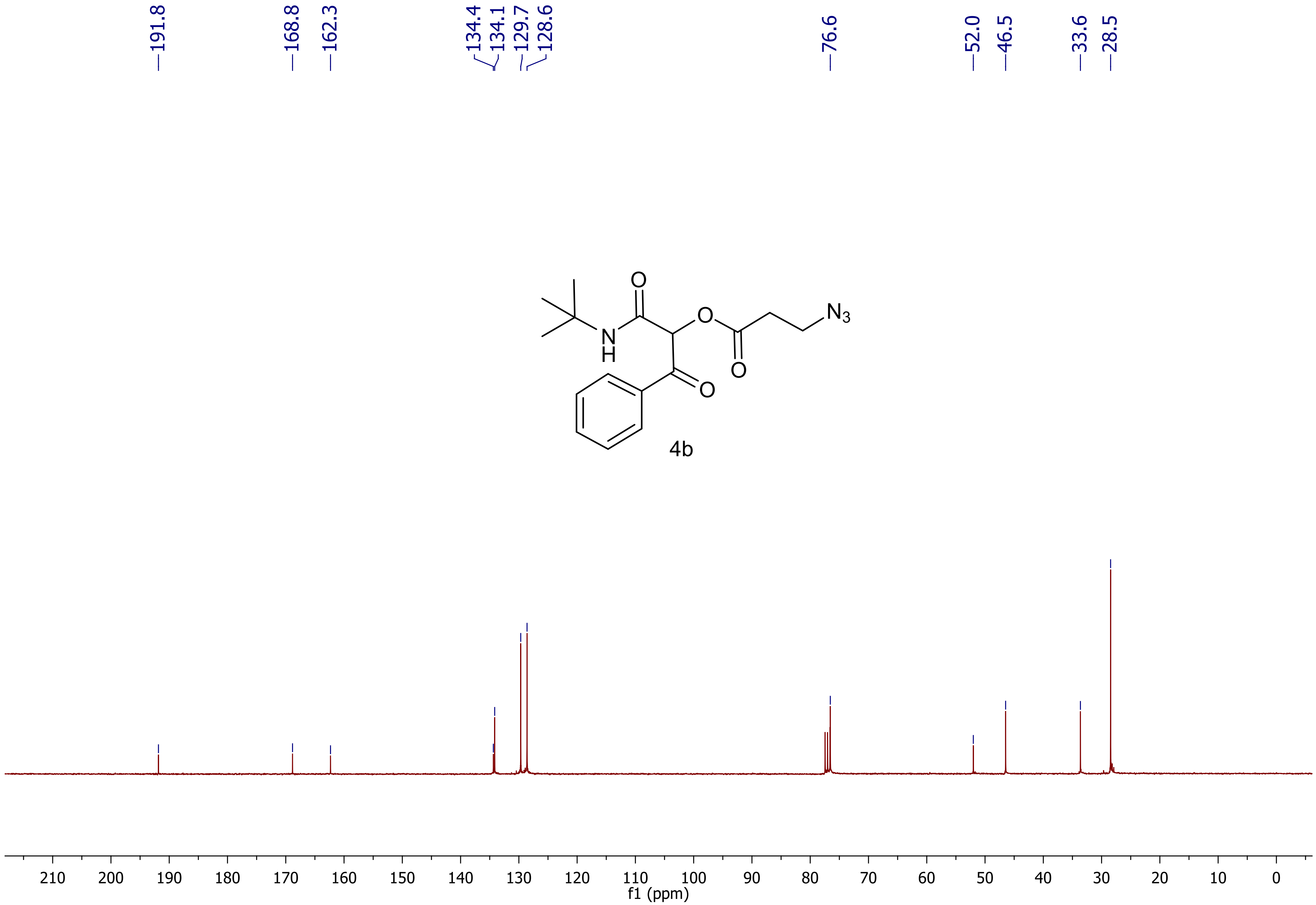
191.8
168.8
162.3
134.4
134.1
129.7
128.6
76.6
52.0
46.5
33.6
28.5
O
O
N
H
N3
O
O
4b
210 200 190 180 170 160 150 140 130 120 110 100 90 80 70 60 50 40 30 20 10 0
f1 (ppm)

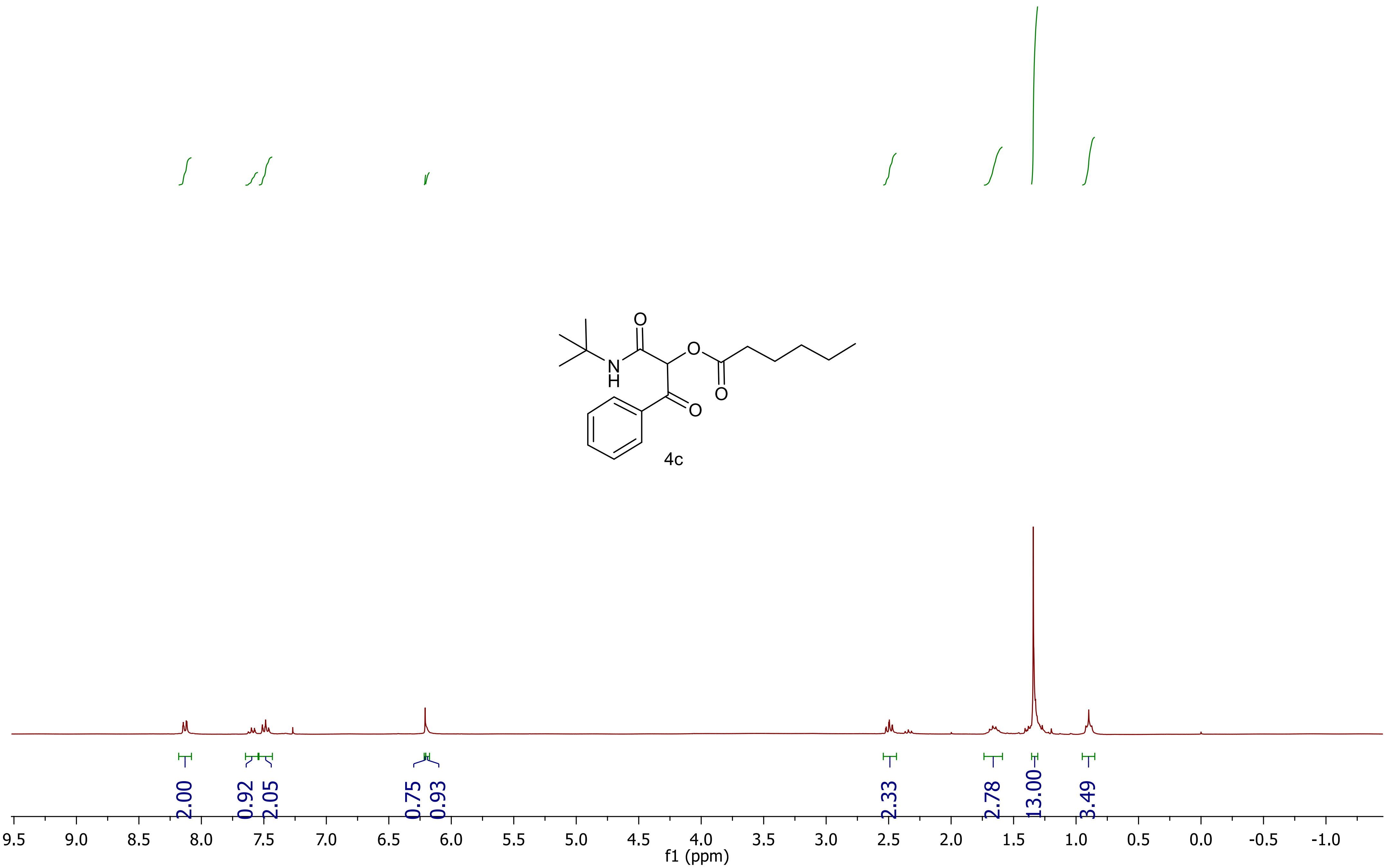

4c
2.00
0.92
2.05
0.75
0.93
2.33
2.78
13.00
3.49
9.5 9.0 8.5 8.0 7.5 7.0 6.5 6.0 5.5 5.0 4.5 4.0 3.5 3.0 2.5 2.0 1.5 1.0 0.5 0.0 -0.5 -1.0
f1 (ppm)

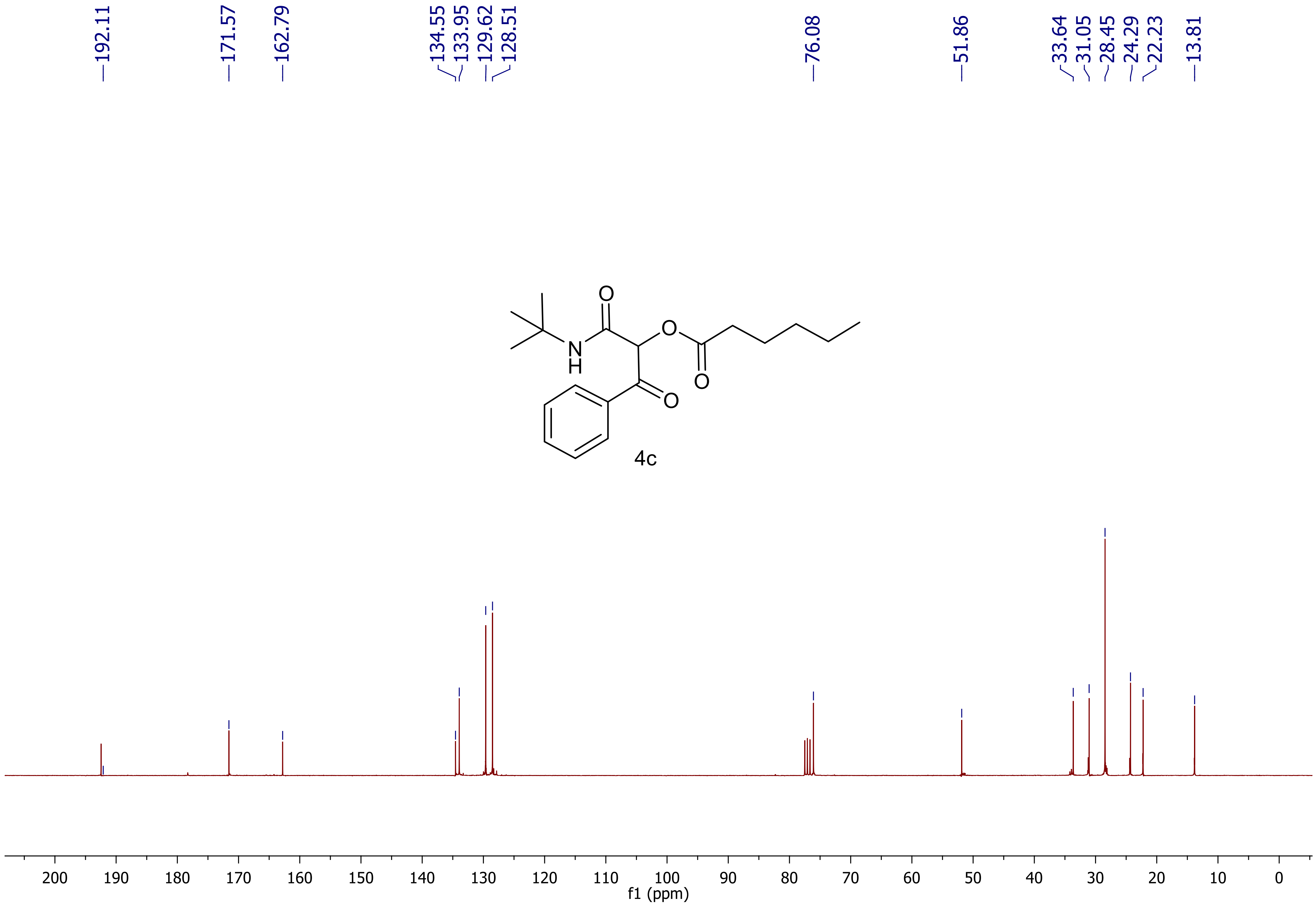
192.11
171.57
162.79
134.55
133.95
129.62
128.51
76.08
51.86
33.64
31.05
28.45
24.29
22.23
13.81
O
N
H
O
O
O
4c
200 190 180 170 160 150 140 130 120 110 100 90 80 70 60 50 40 30 20 10 0
f1 (ppm)

Mixture of Diastereomers

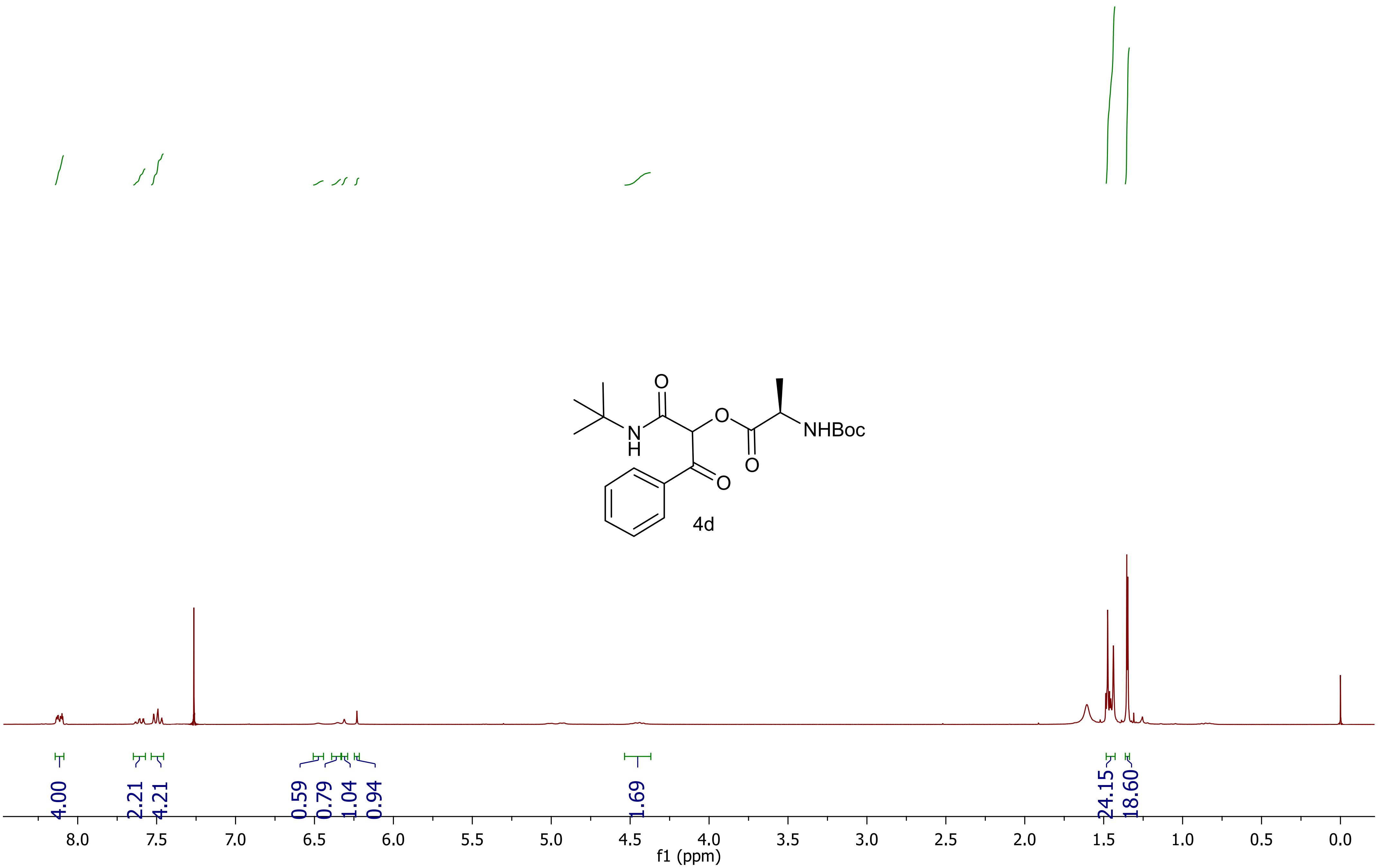

Mixture of Diastereomers

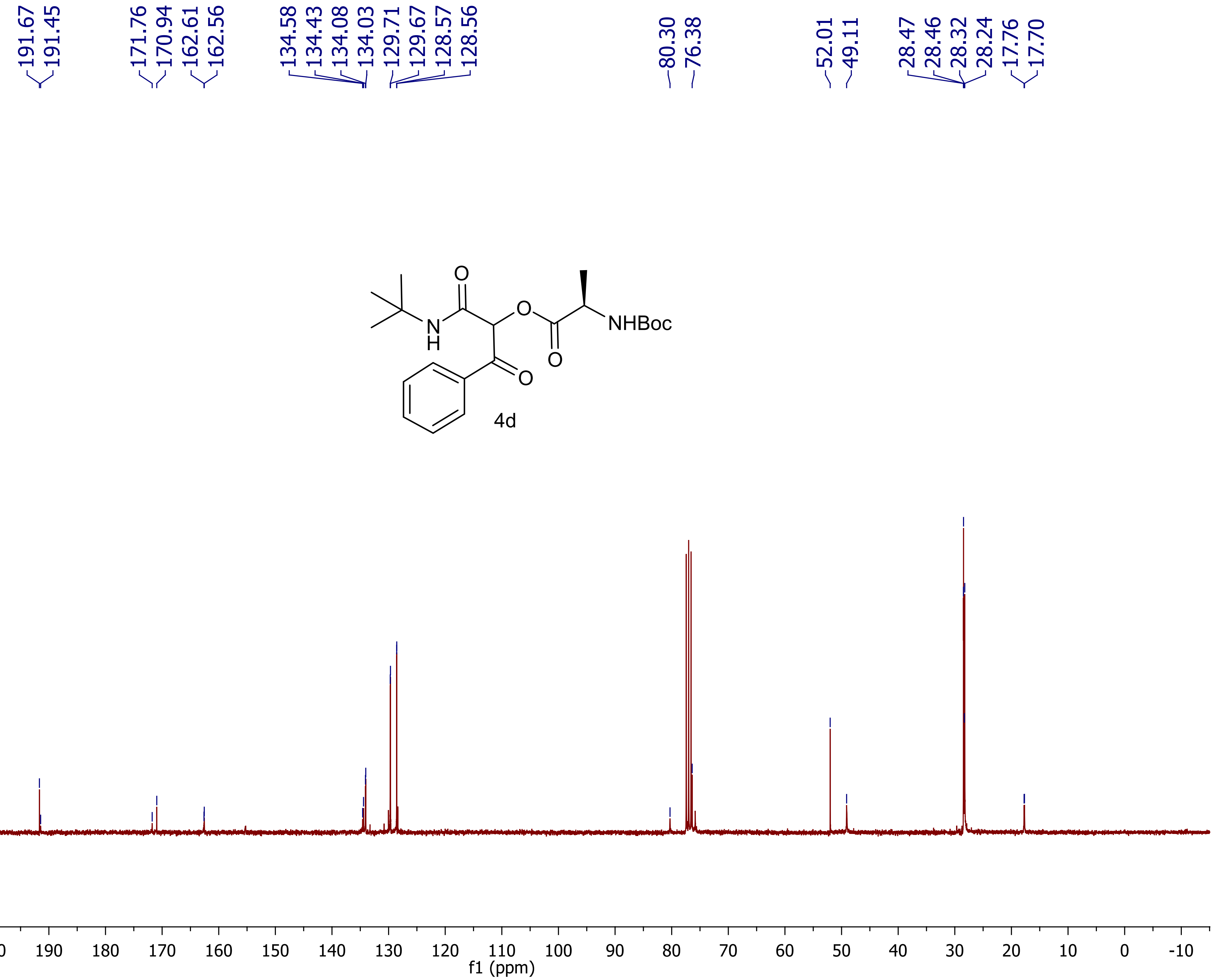

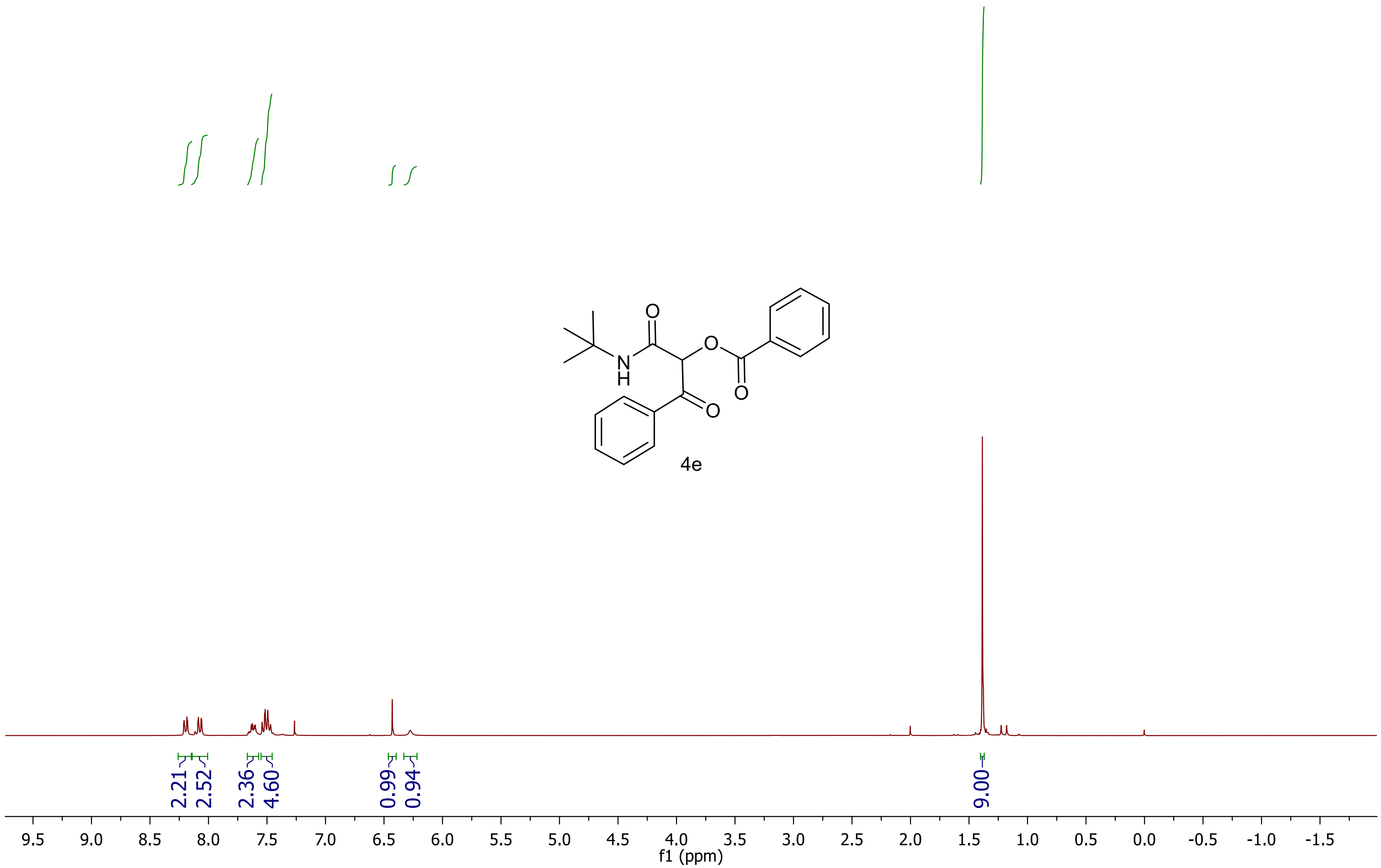

O
O
N
H
O
O
O
4e
2.21
2.52
2.36
4.60
0.99
0.94
9.00
9.5 9.0 8.5 8.0 7.5 7.0 6.5 6.0 5.5 5.0 4.5 4.0 3.5 3.0 2.5 2.0 1.5 1.0 0.5 0.0 -0.5 -1.0 -1.5
f1 (ppm)

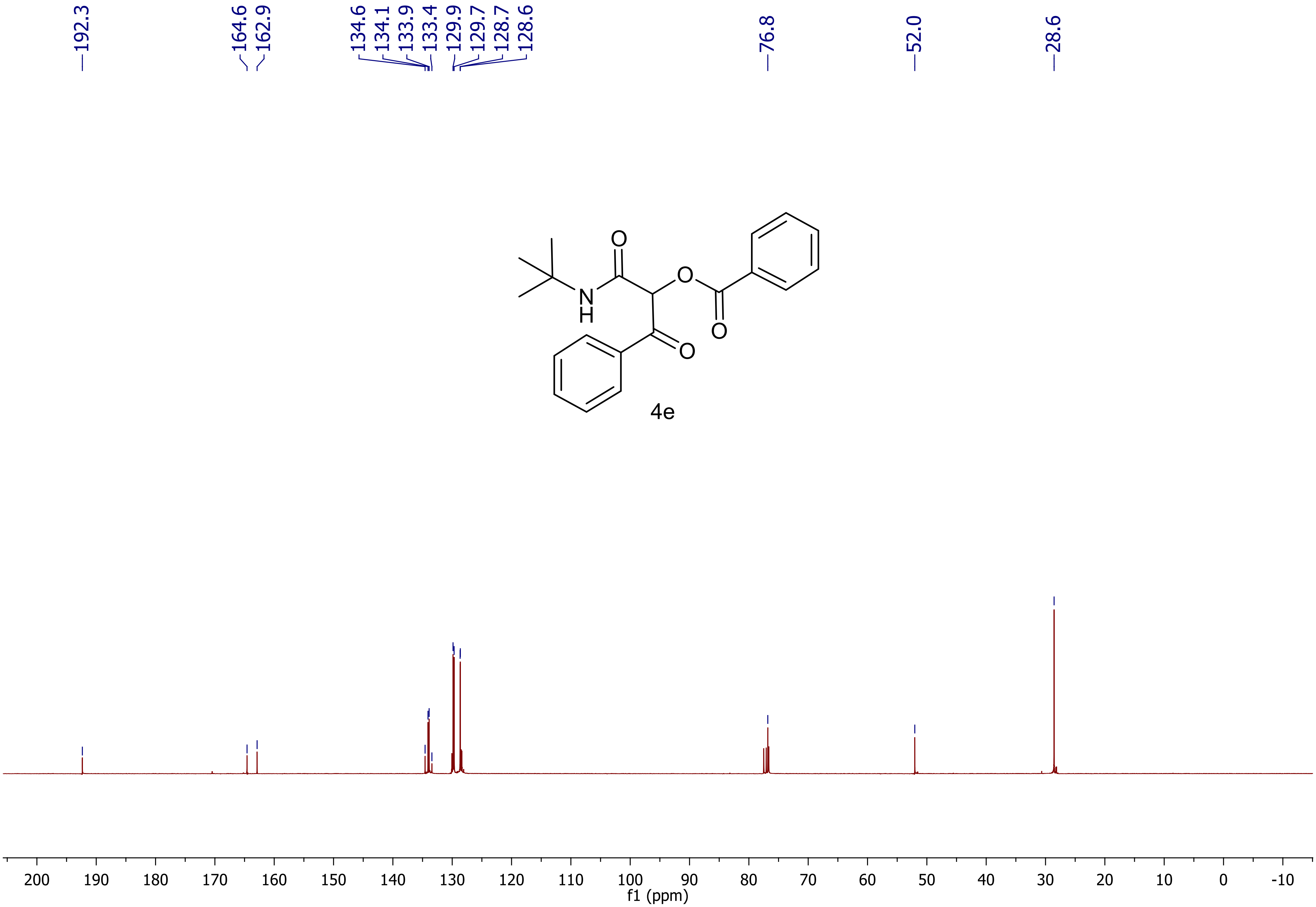
192.3
164.6
162.9
134.6
134.1
133.9
133.4
129.9
129.7
128.7
128.6
76.8
52.0
28.6
O
O
N
H
O
O
4e
200
190
180
170
160
150
140
130
120
110
100
90
80
70
60
50
40
30
20
10
0
-10
f1 (ppm)

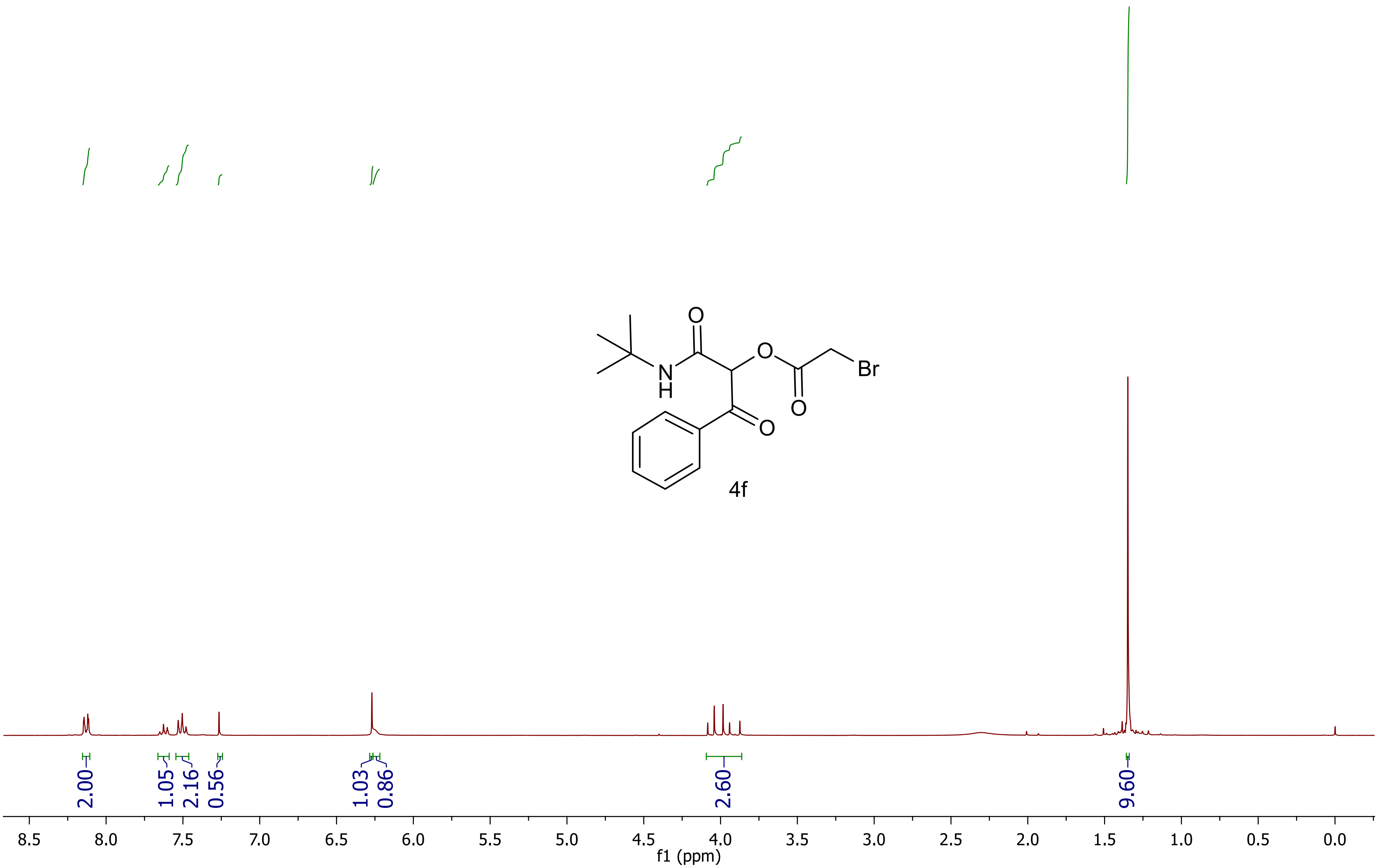

O
N
H
O
O
Br
O
4f
2.00
1.05
2.16
0.56
1.03
0.86
2.60
9.60
8.5 8.0 7.5 7.0 6.5 6.0 5.5 5.0 4.5 4.0 3.5 3.0 2.5 2.0 1.5 1.0 0.5 0.0
f1 (ppm)

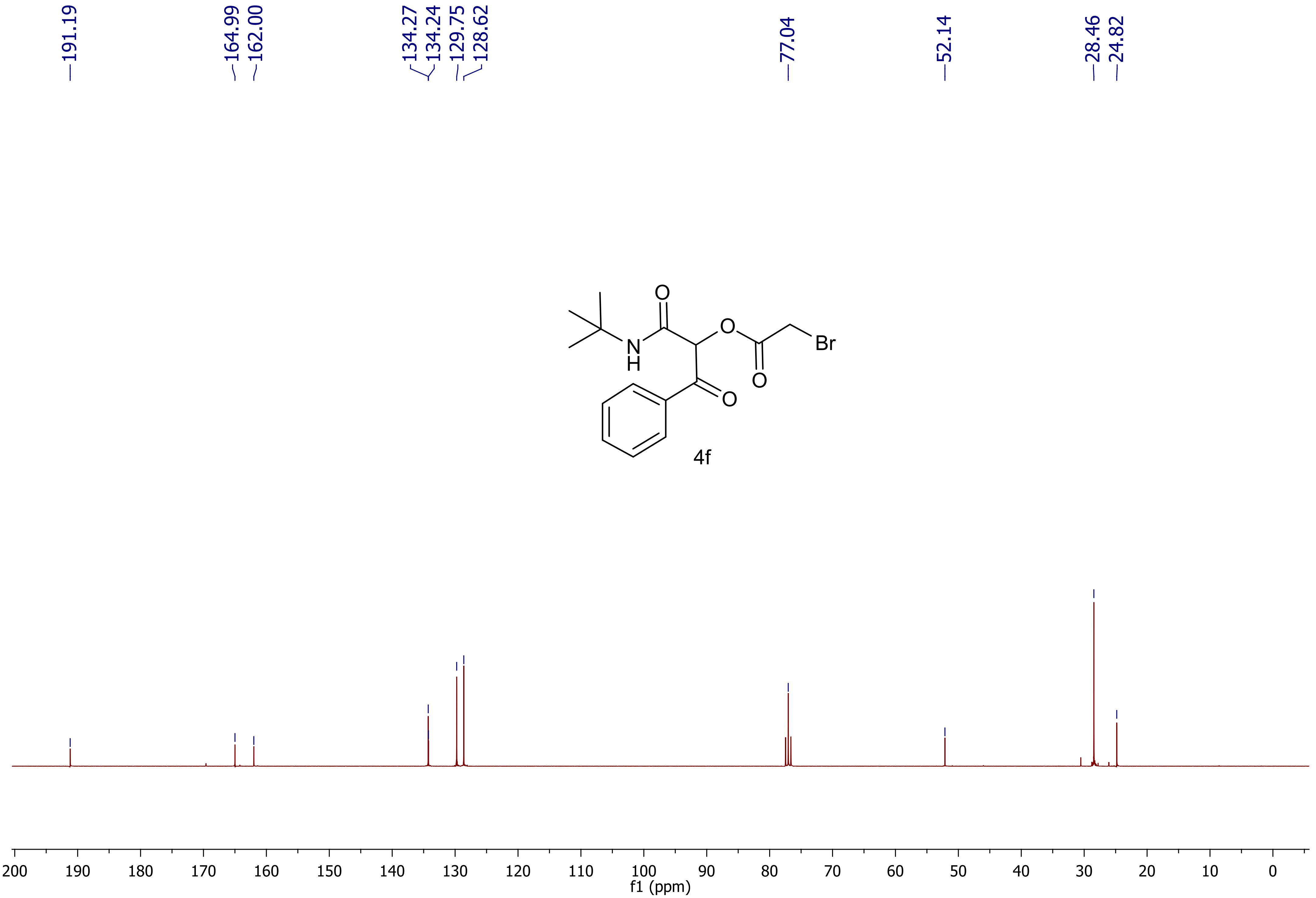

191.19
164.99
162.00
134.27
134.24
129.75
128.62
77.04
52.14
28.46
24.82
O
O
N
H
O
O
Br
O
4f
200
190
180
170
160
150
140
130
120
110
100
90
80
70
60
50
40
30
20
10
0
f1 (ppm)

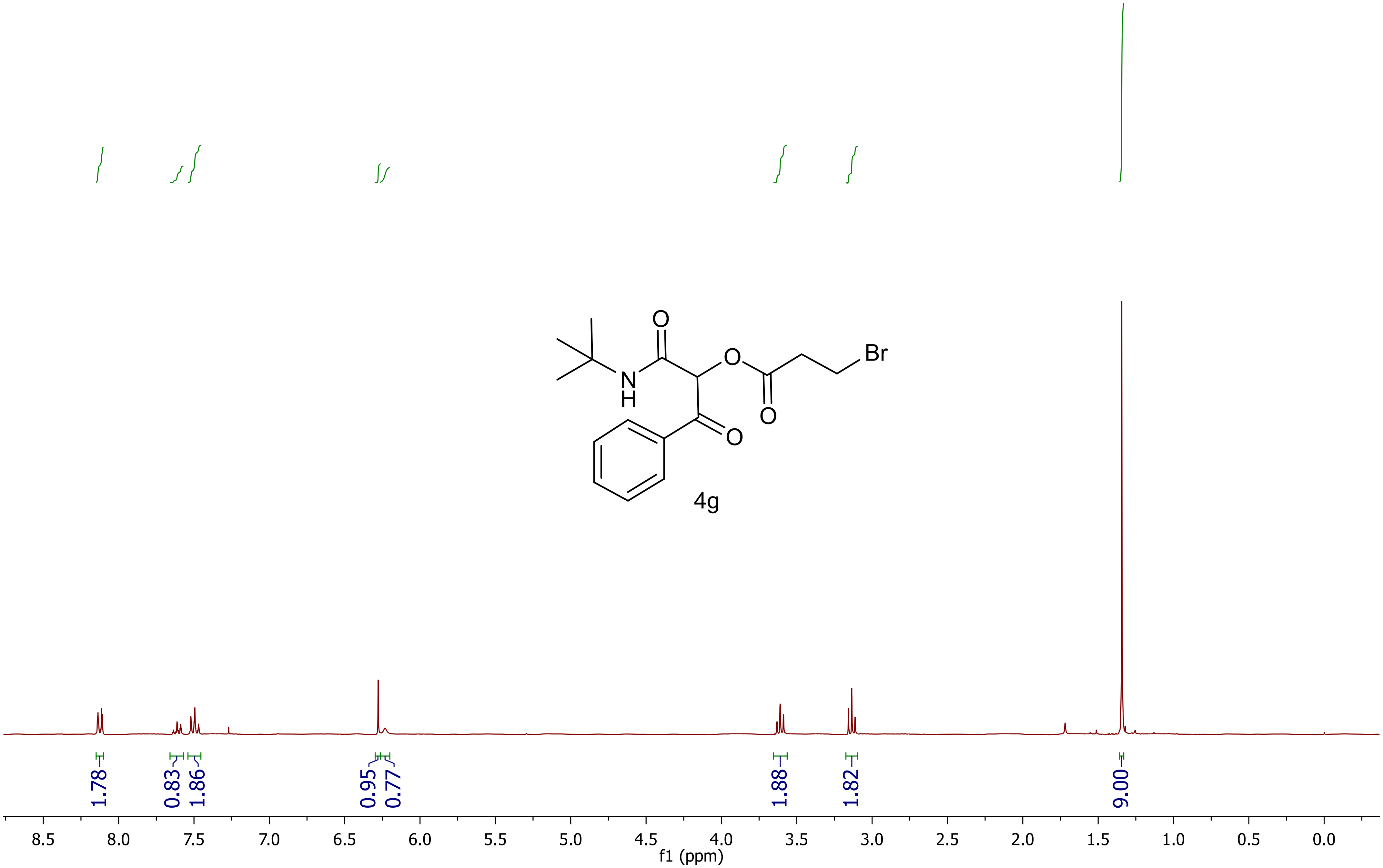

O
O
Br
N
H
O
O
4g
1.78
0.83
1.86
0.95
0.77
1.88
1.82
9.00
8.5
8.0
7.5
7.0
6.5
6.0
5.5
5.0
4.5
4.0
3.5
3.0
2.5
2.0
1.5
1.0
0.5
0.0
f1 (ppm)

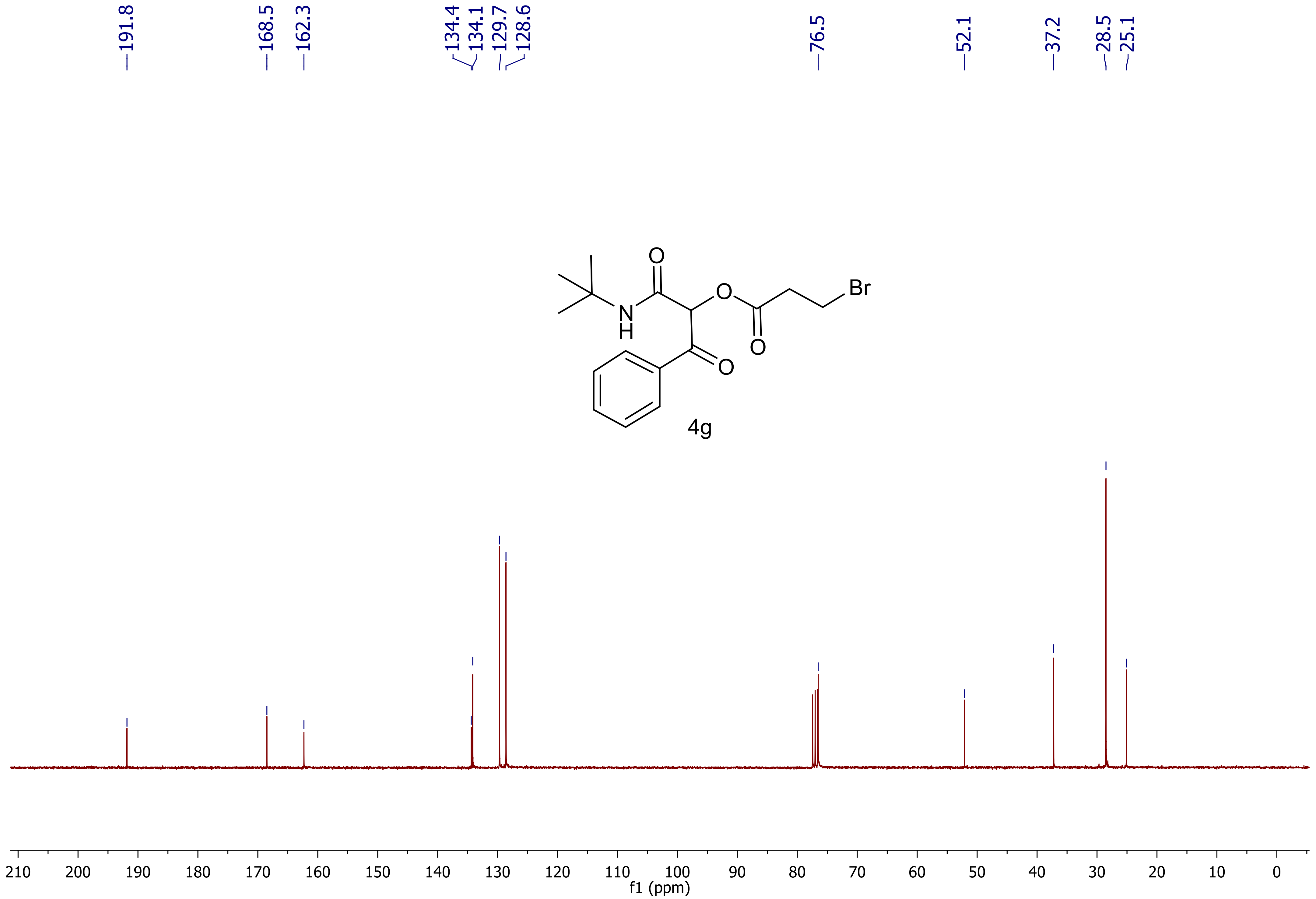
191.8
168.5
162.3
134.4
134.1
129.7
128.6
76.5
52.1
37.2
28.5
25.1
O
N
H
O
Br
O
O
4g
210 200 190 180 170 160 150 140 130 120 110 100 90 80 70 60 50 40 30 20 10 0
f1 (ppm)

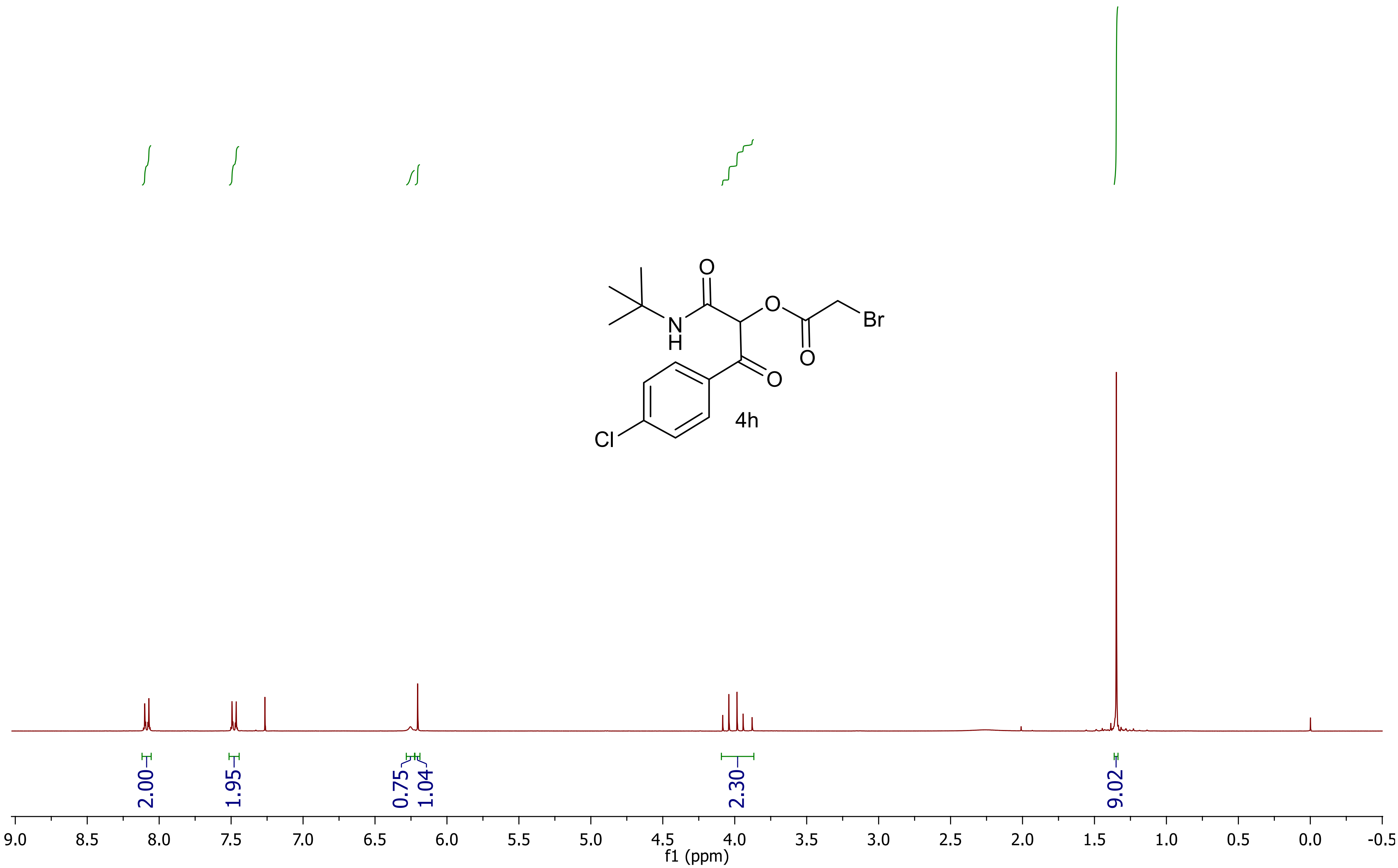

O
N
H
O
O
O
Br
Cl
4h
2.00
1.95
0.75
1.04
2.30
9.02
9.0
8.5
8.0
7.5
7.0
6.5
6.0
5.5
5.0
4.5
4.0
3.5
3.0
2.5
2.0
1.5
1.0
0.5
0.0
-0.5
f1 (ppm)

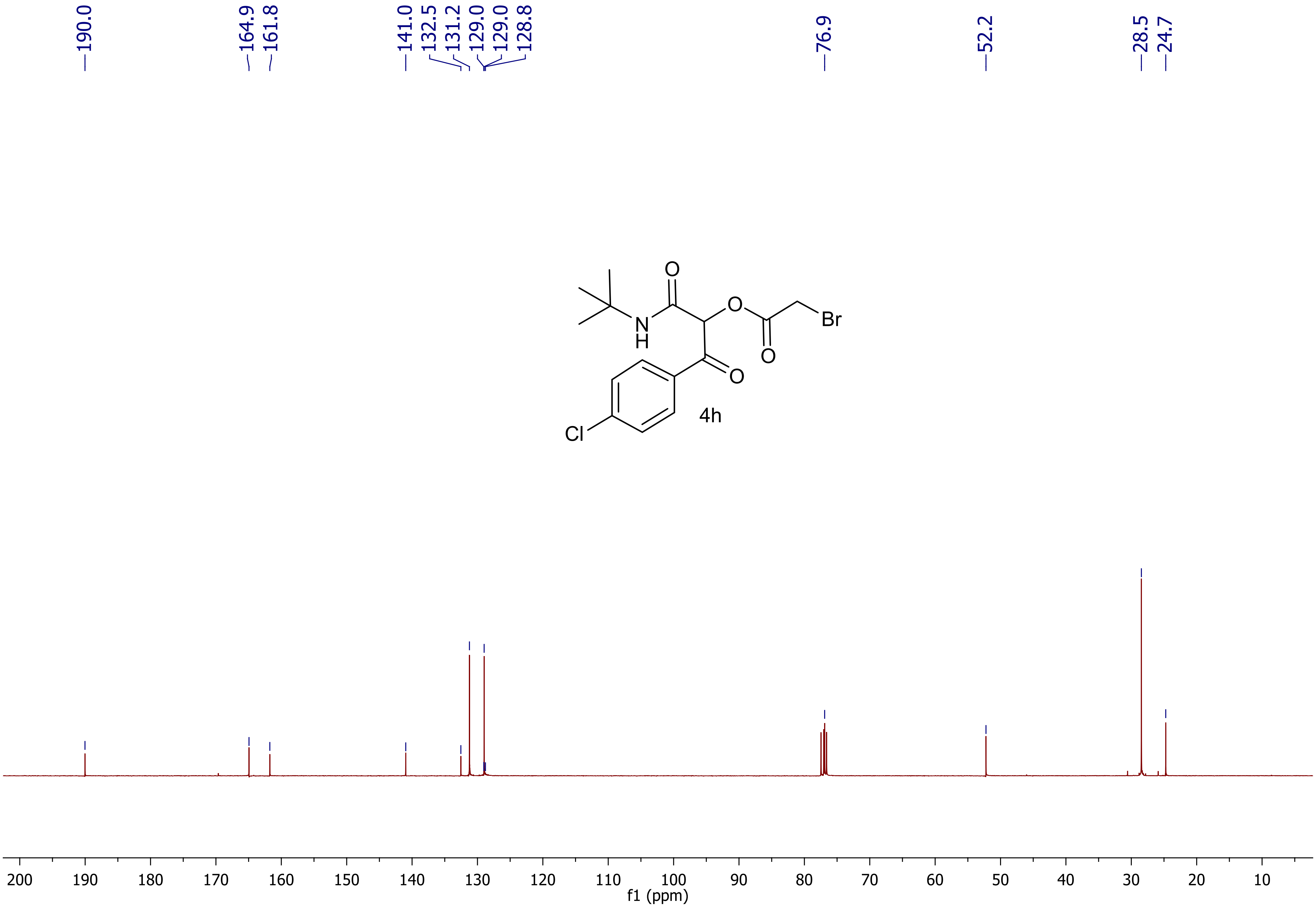
190.0
164.9
161.8
141.0
132.5
131.2
129.0
129.0
128.8
76.9
52.2
28.5
24.7
O
O
N
H
O
O
Br
O
Cl
4h
200
190
180
170
160
150
140
130
120
110
100
90
80
70
60
50
40
30
20
10
f1 (ppm)

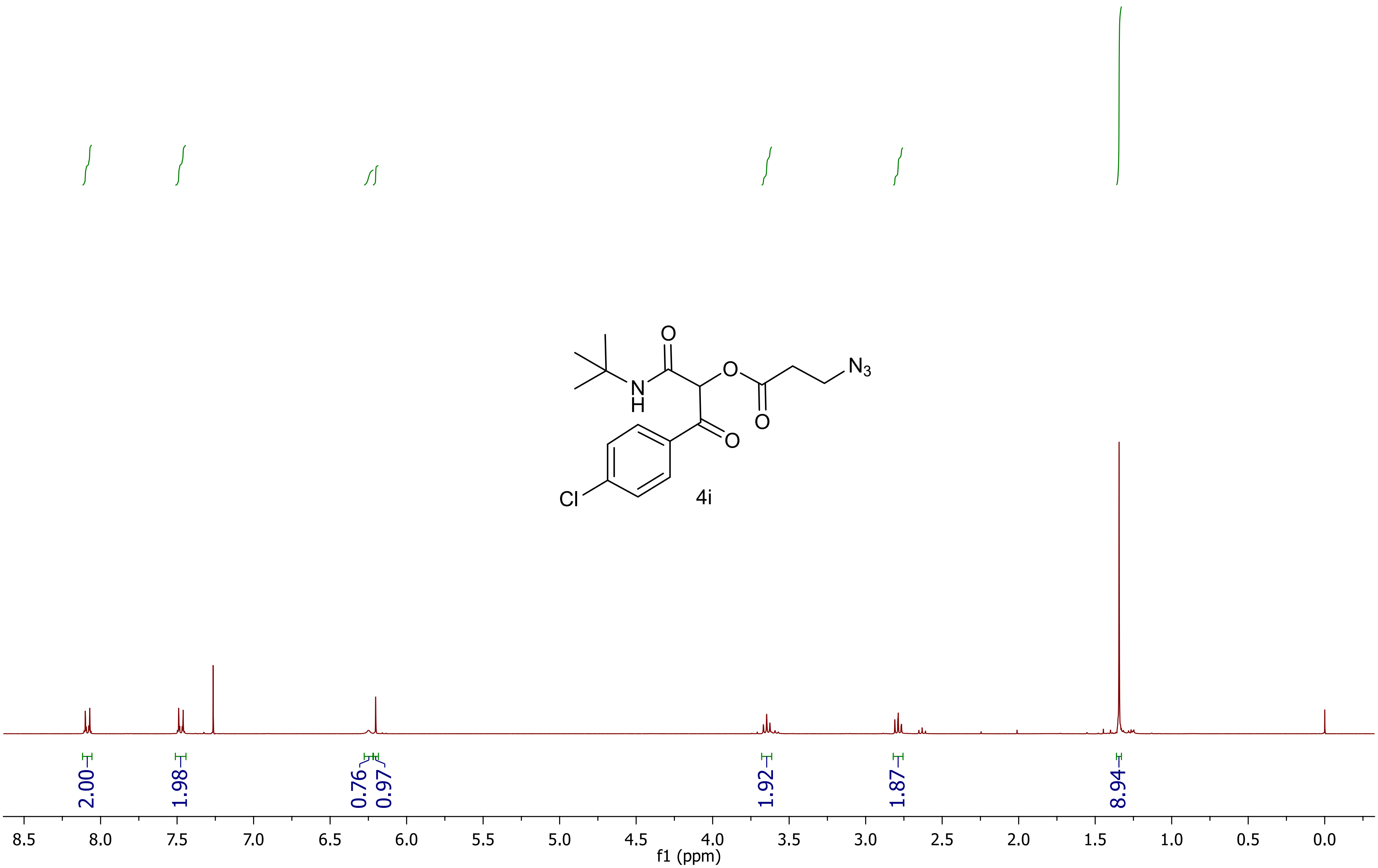

O
O
N
H
O
N3
O
O
Cl
4i
2.00
1.98
0.76
0.97
1.92
1.87
8.94
8.5
8.0
7.5
7.0
6.5
6.0
5.5
5.0
4.5
4.0
3.5
3.0
2.5
2.0
1.5
1.0
0.5
0.0
f1 (ppm)

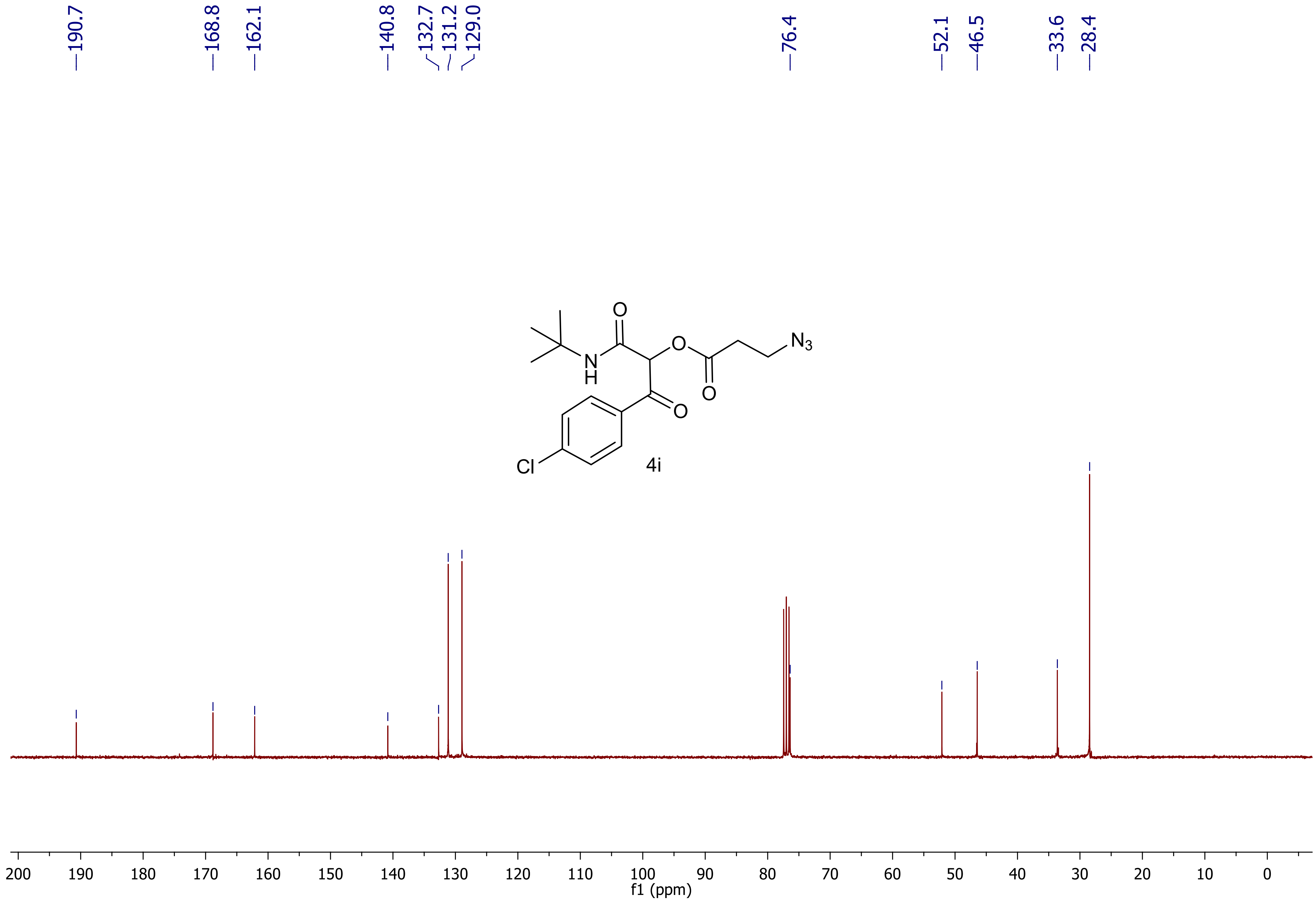
190.7
168.8
162.1
140.8
132.7
131.2
129.0
76.4
52.1
46.5
33.6
28.4
O
O
N
H
O
N3
O
O
Cl
4i
200
190
180
170
160
150
140
130
120
110
100
90
80
70
60
50
40
30
20
10
0
f1 (ppm)

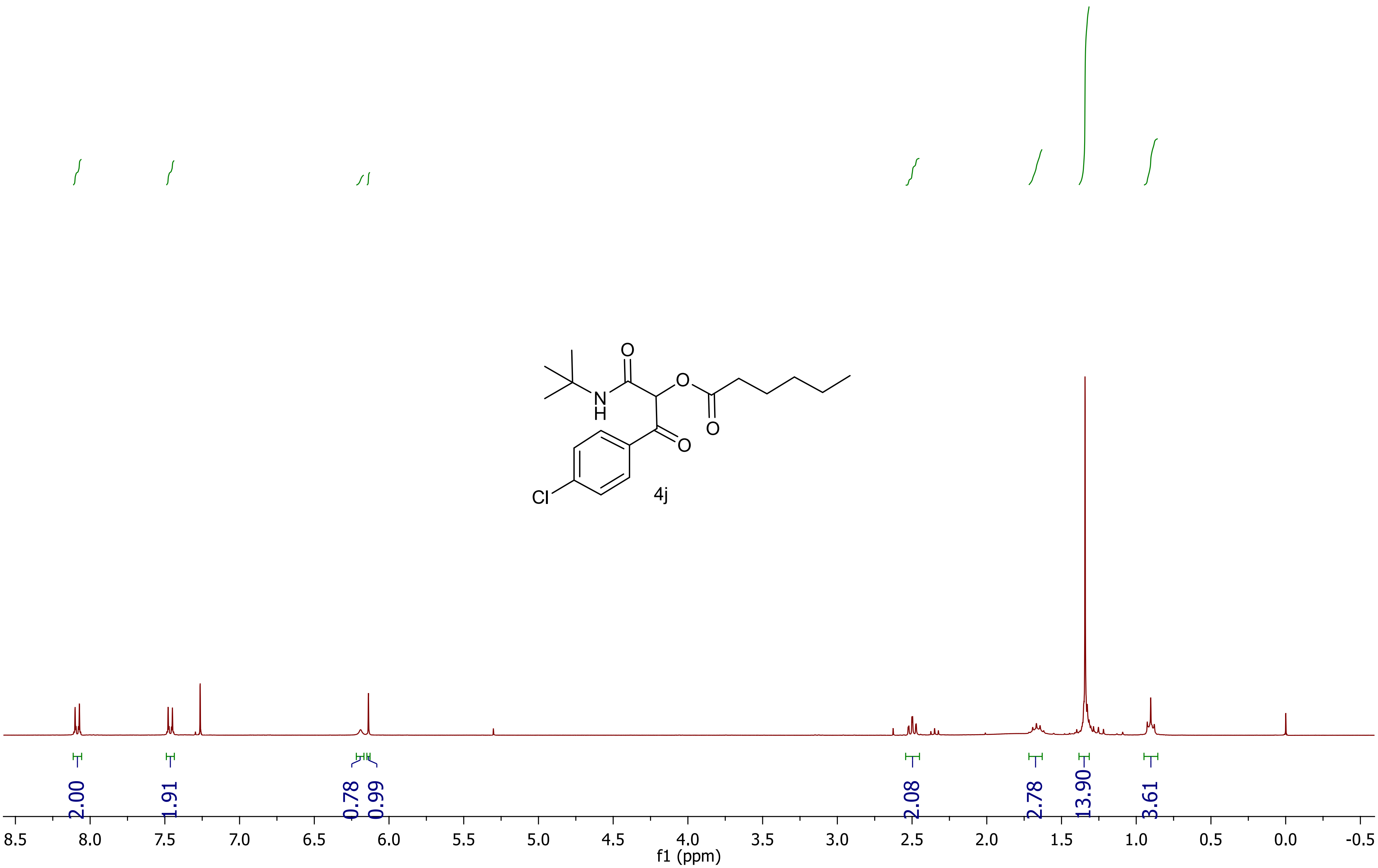

O
N
H
O
O
O
Cl
4j
2.00
1.91
0.78
0.99
2.08
2.78
13.90
3.61
8.5
8.0
7.5
7.0
6.5
6.0
5.5
5.0
4.5
4.0
3.5
3.0
2.5
2.0
1.5
1.0
0.5
0.0
-0.5
f1 (ppm)

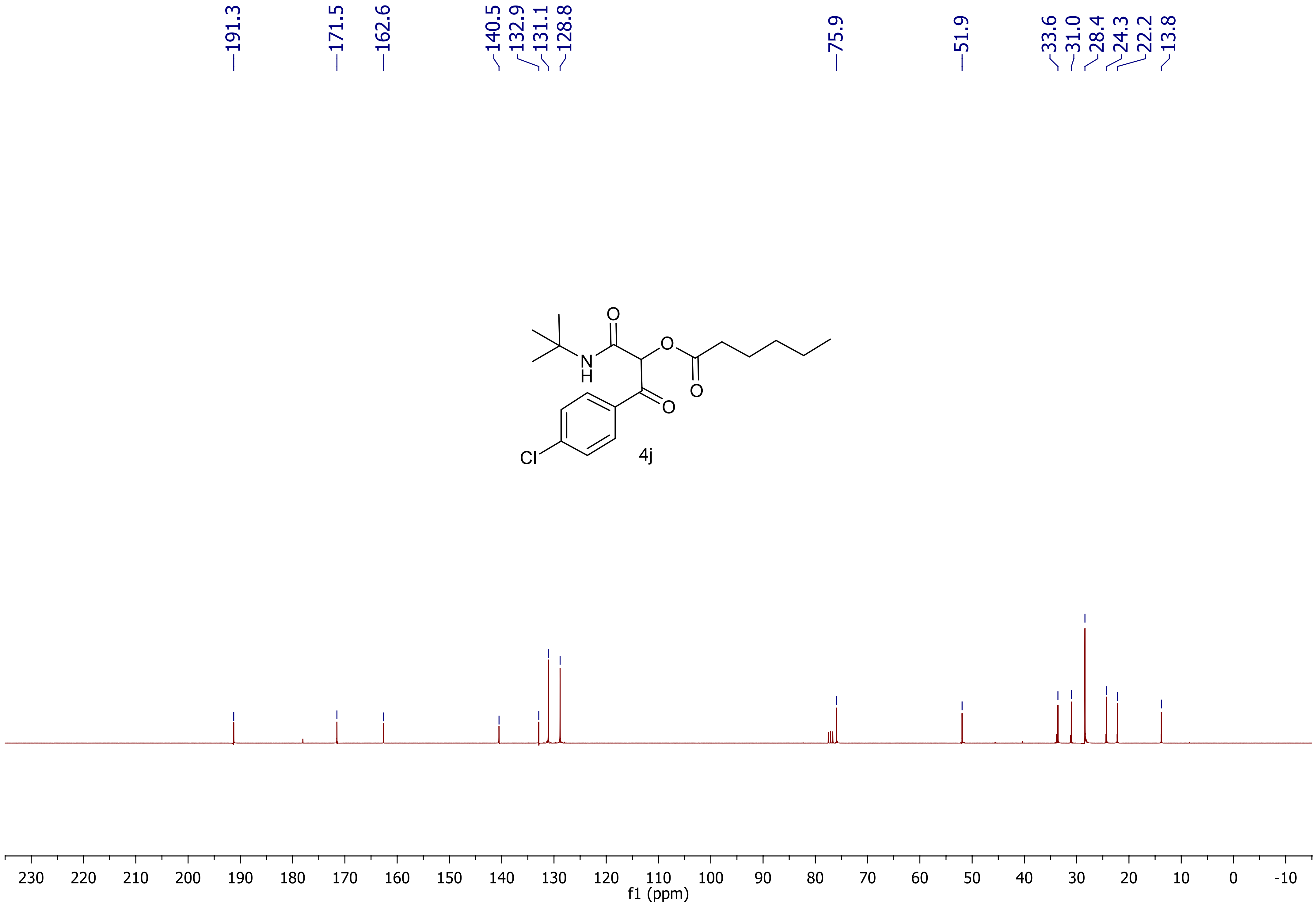
191.3
171.5
162.6
140.5
132.9
131.1
128.8
75.9
51.9
33.6
31.0
28.4
24.3
22.2
13.8
O
N
H
O
O
O
Cl
4j
230 220 210 200 190 180 170 160 150 140 130 120 110 100 90 80 70 60 50 40 30 20 10 0 -10
f1 (ppm)

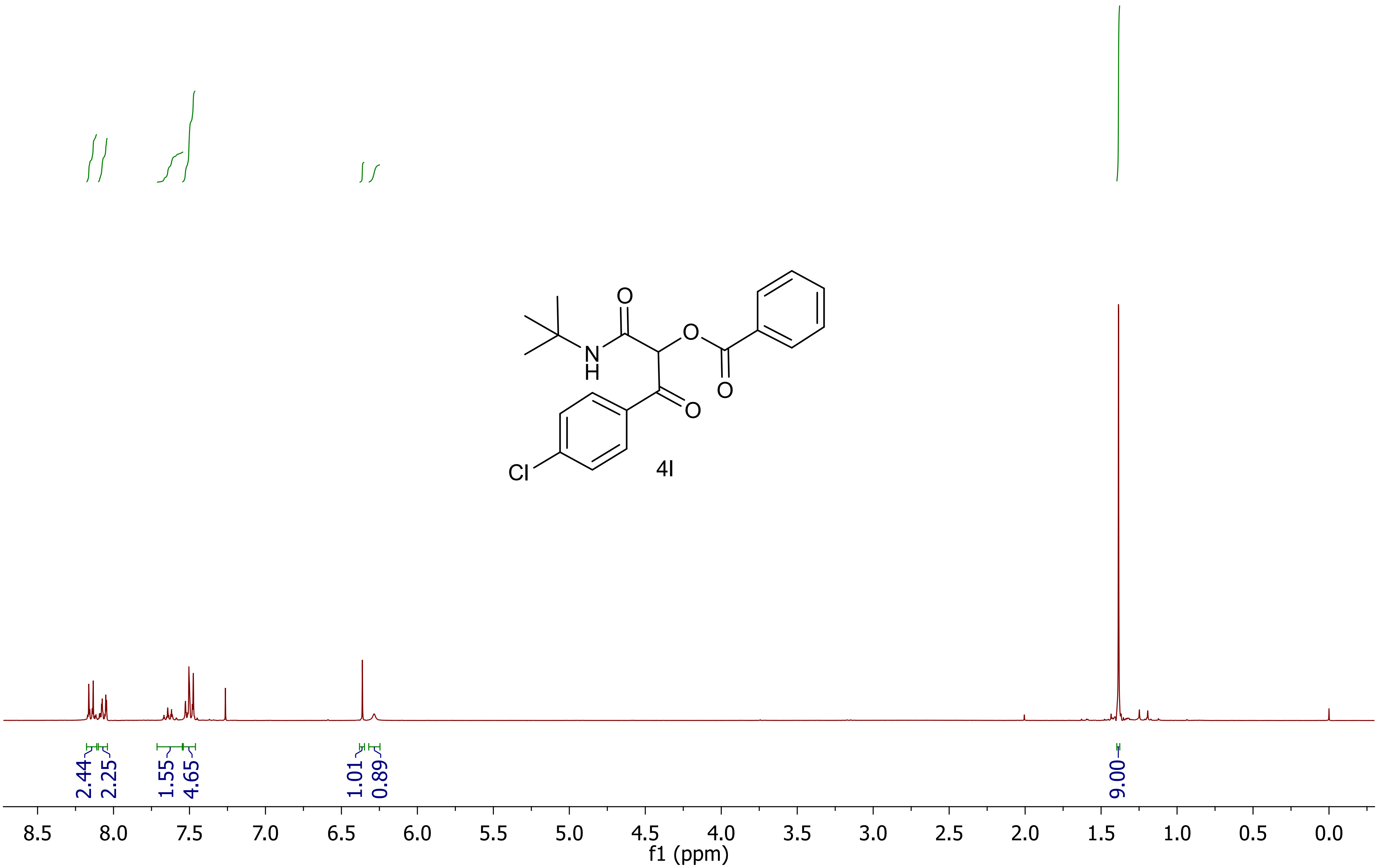

O
O
N
H
O
O
Cl
4l
2.44
2.25
1.55
4.65
1.01
0.89
9.00
8.5
8.0
7.5
7.0
6.5
6.0
5.5
5.0
4.5
4.0
3.5
3.0
2.5
2.0
1.5
1.0
0.5
0.0
f1 (ppm)

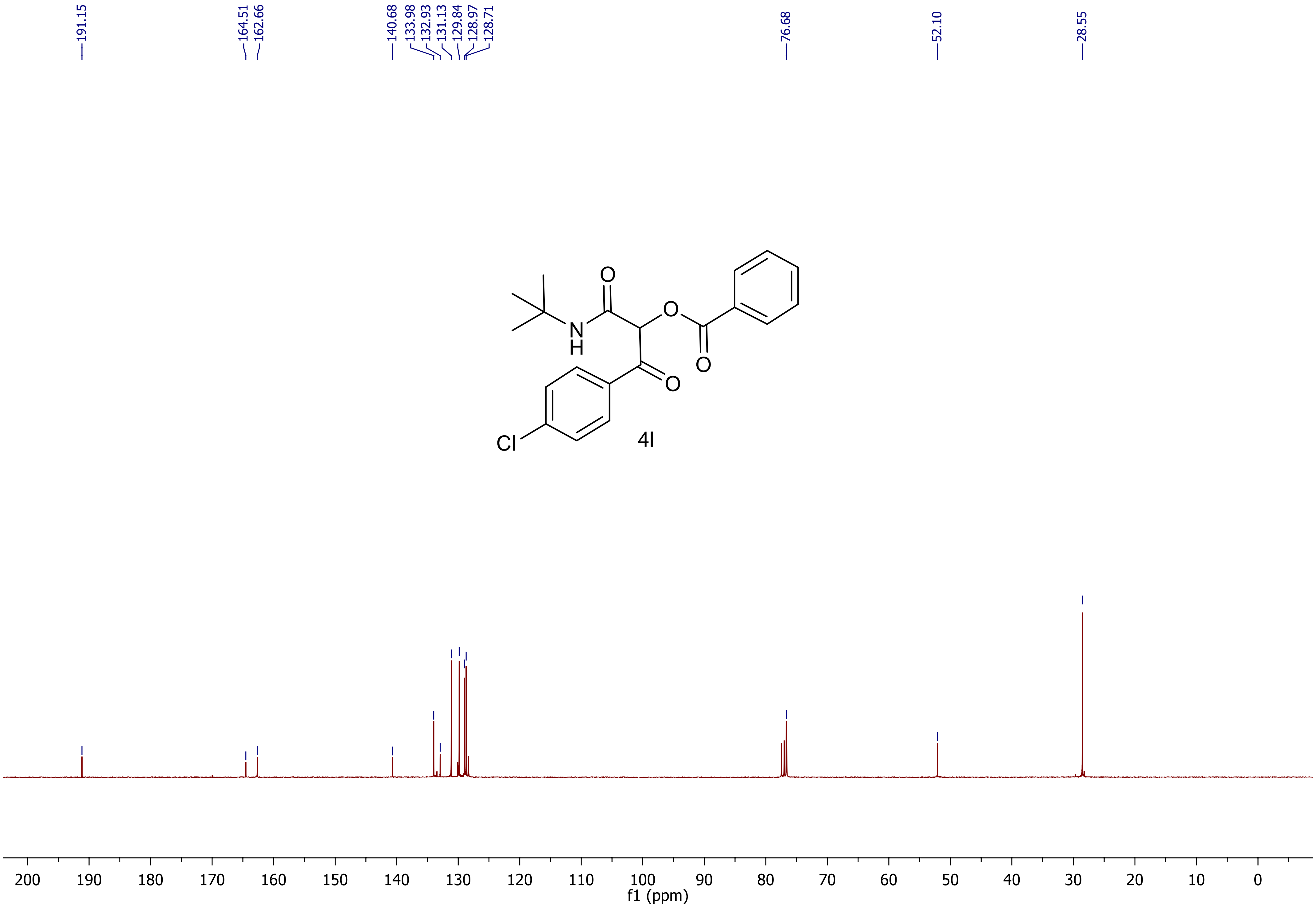
191.15
164.51
162.66
140.68
133.98
132.93
131.13
129.84
128.97
128.71
76.68
52.10
28.55
O
O
N
H
O
O
Cl
4l
200 190 180 170 160 150 140 130 120 110 100 90 80 70 60 50 40 30 20 10 0
f1 (ppm)

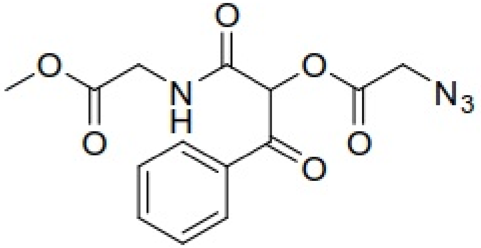

O
N
H
O
O
O
N3
O
O

4m

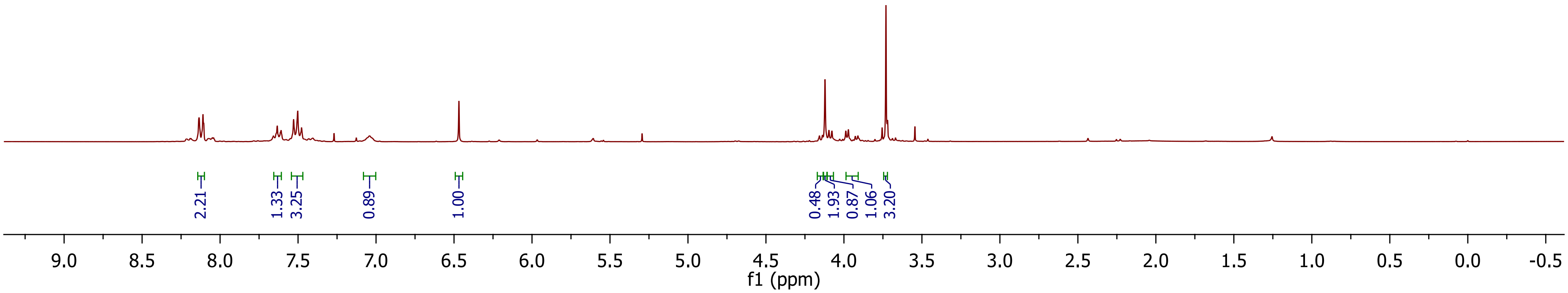

2.21
1.33
3.25
0.89
1.00
0.48
1.93
0.87
1.06
3.20
9.0
8.5
8.0
7.5
7.0
6.5
6.0
5.5
5.0
4.5
4.0
3.5
3.0
2.5
2.0
1.5
1.0
0.5
0.0
-0.5
f1 (ppm)

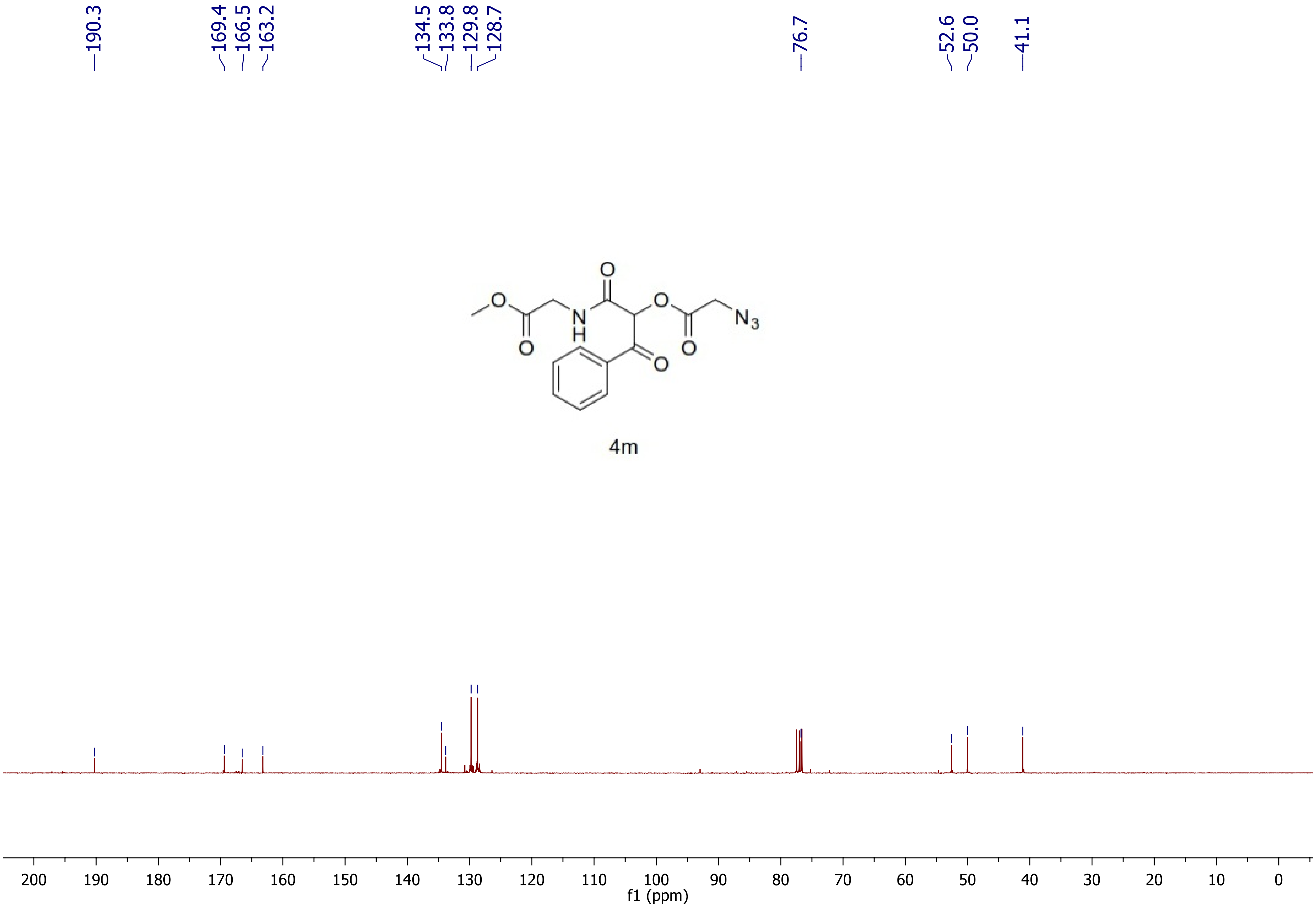
190.3
169.4
166.5
163.2
134.5
133.8
129.8
128.7
76.7
52.6
50.0
41.1
4m
200
190
180
170
160
150
140
130
120
110
100
90
80
70
60
50
40
30
20
10
0
f1 (ppm)

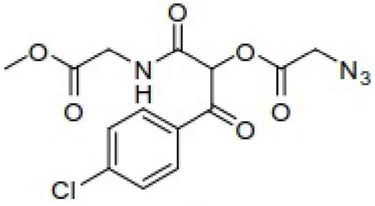
N3
Cl

4n

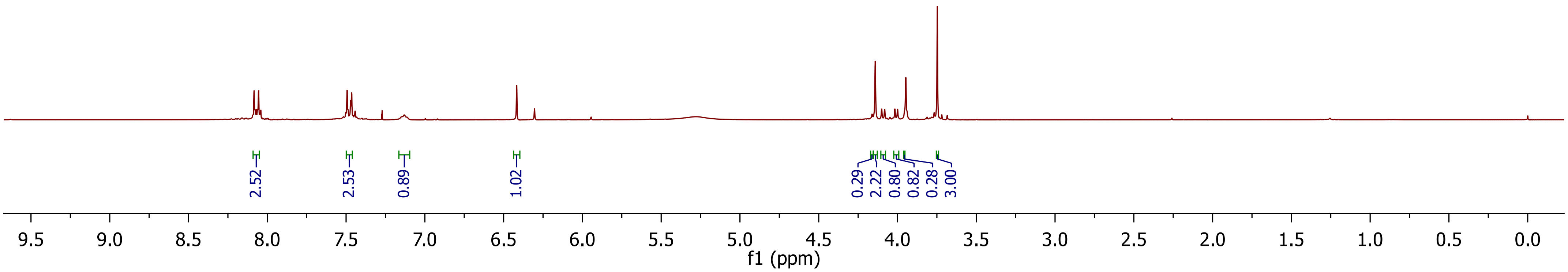
2.52
2.53
0.89
1.02
0.29
2.22
0.80
0.82
0.28
3.00
9.5 9.0 8.5 8.0 7.5 7.0 6.5 6.0 5.5 5.0 4.5 4.0 3.5 3.0 2.5 2.0 1.5 1.0 0.5 0.0
f1 (ppm)

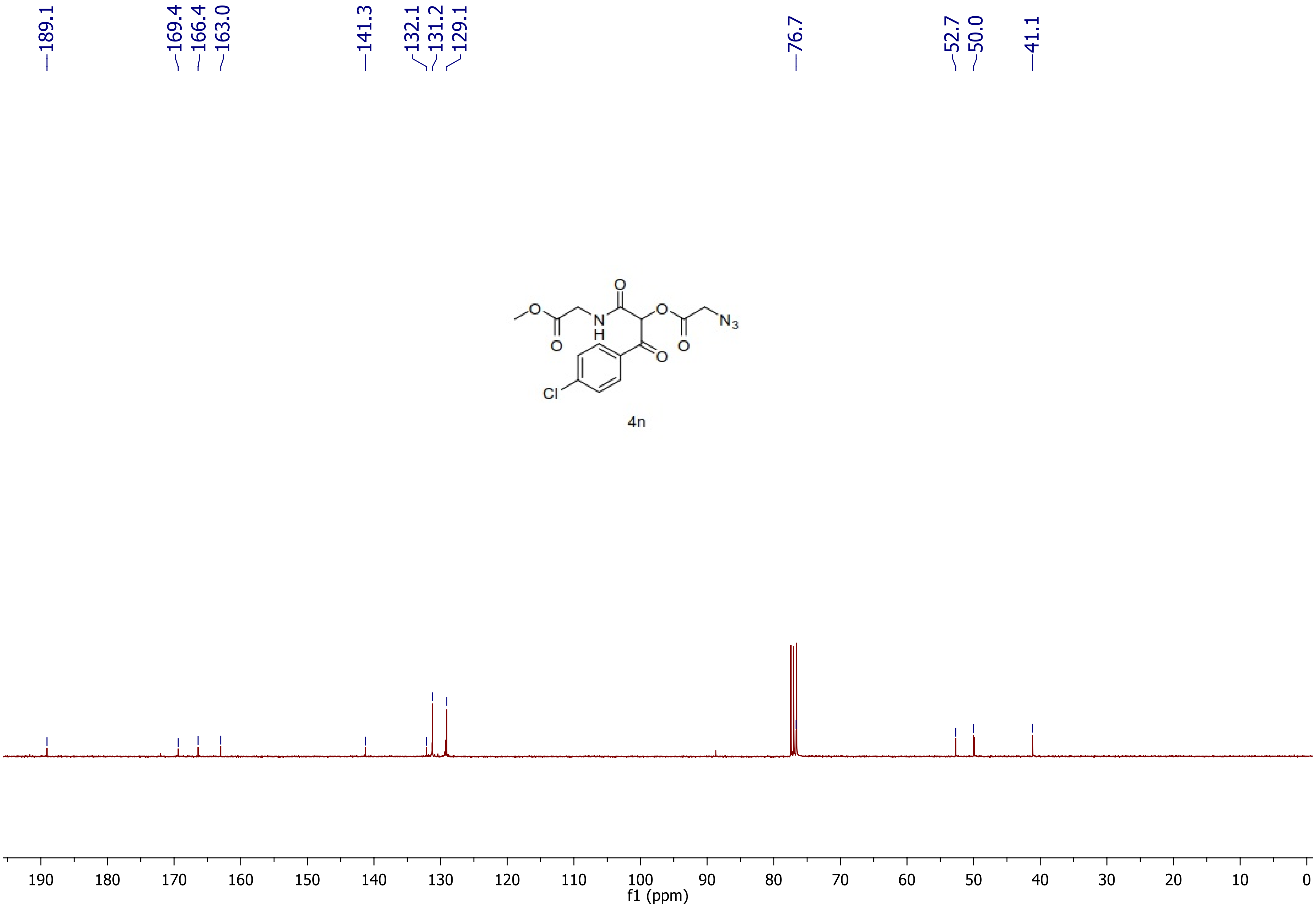
189.1
169.4
166.4
163.0
141.3
132.1
131.2
129.1
76.7
52.7
50.0
41.1
O
O
N
H
O
O
N3
O
O
Cl
4n
190 180 170 160 150 140 130 120 110 100 90 80 70 60 50 40 30 20 10 0
f1 (ppm)

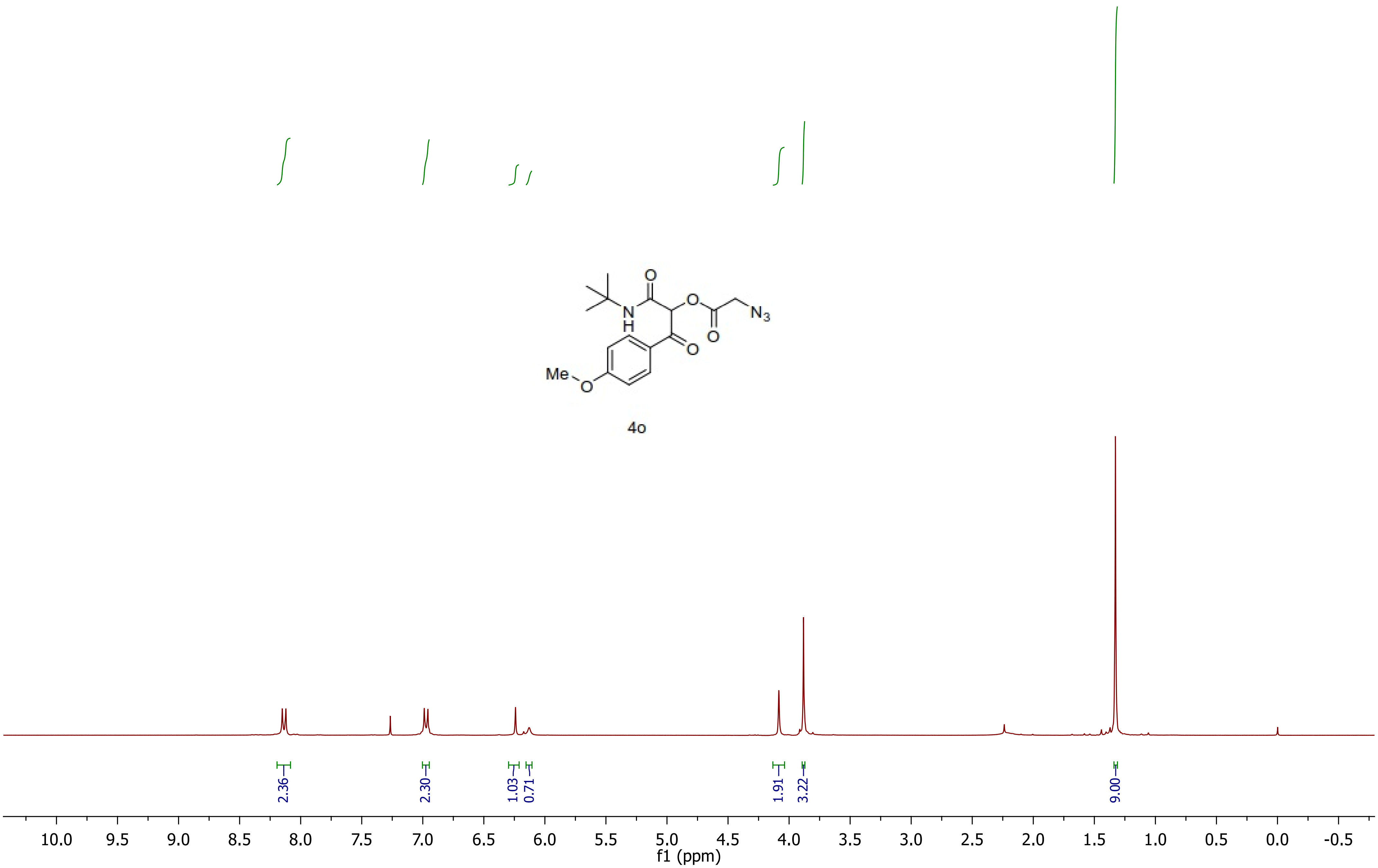

Me
O
O
N
H
O
O
O
N_3
4o
2.36
2.30
1.03
0.71
1.91
3.22
9.00
10.0 9.5 9.0 8.5 8.0 7.5 7.0 6.5 6.0 5.5 5.0 4.5 4.0 3.5 3.0 2.5 2.0 1.5 1.0 0.5 0.0 -0.5
f1 (ppm)

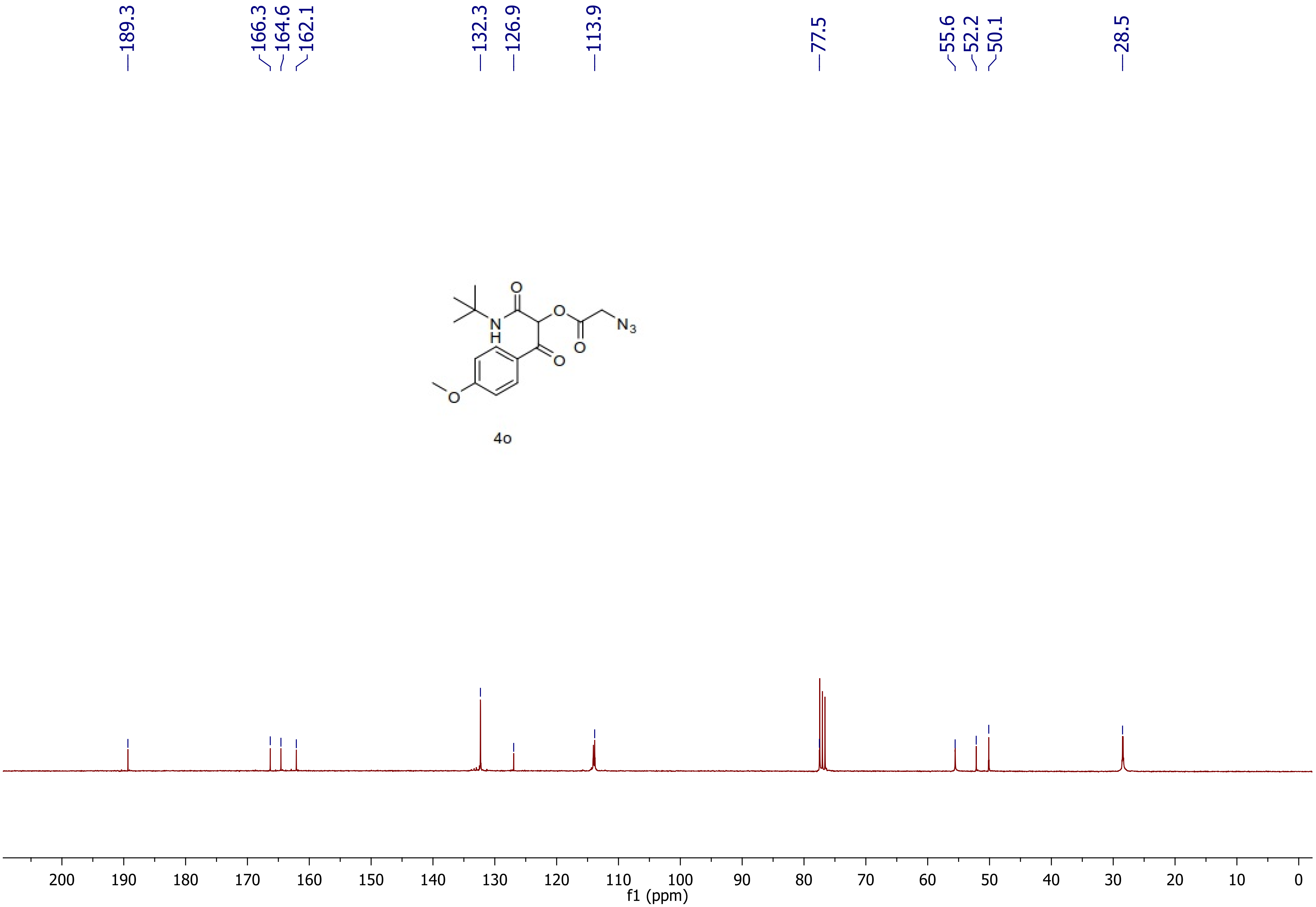
189.3
166.3
164.6
162.1
132.3
126.9
113.9
77.5
55.6
52.2
50.1
28.5
O
N
H
O
O
N3
O
O
O
4o
200
190
180
170
160
150
140
130
120
110
100
90
80
70
60
50
40
30
20
10
0
f1 (ppm)

Mass Spectra - S3

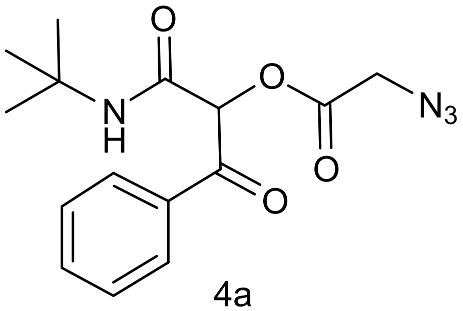

4a

Acquisition Parameter

Source Type	ESI	Ion Polarity	Positive	Set Nebulizer	2.0 Bar
Focus	Active	Set Capillary	4500 V	Set Dry Heater	180 °C
Scan Begin	50 m/z	Set End Plate Offset	-500 V	Set Dry Gas	9.0 l/min
Scan End	1000 m/z	Set Charging Voltage	2000 V	Set Divert Valve	Waste
		Set Corona	0 nA	Set APCI Heater	0 °C

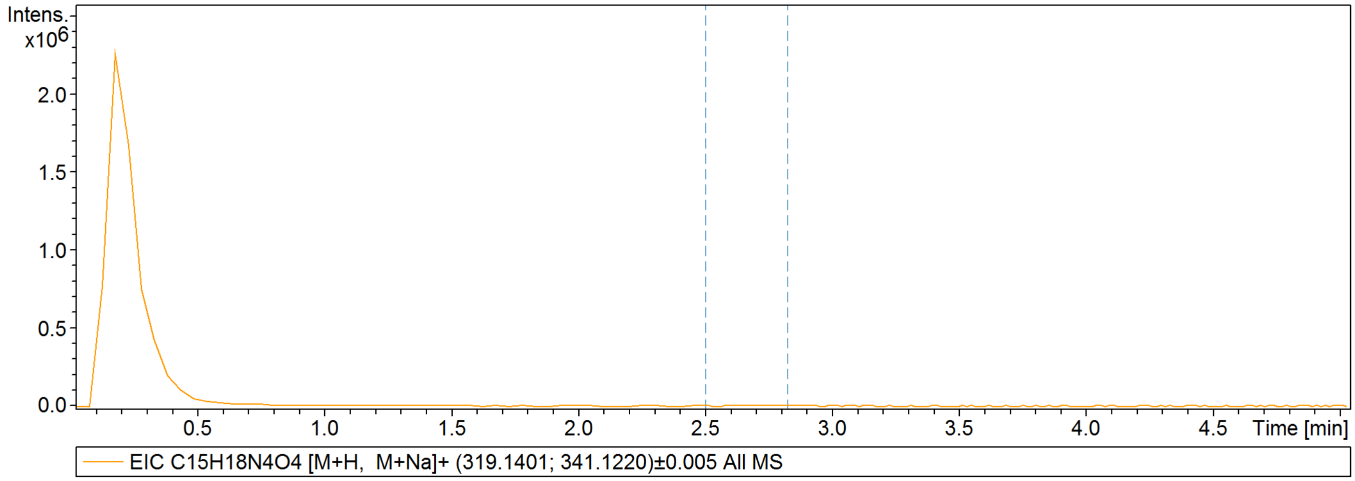

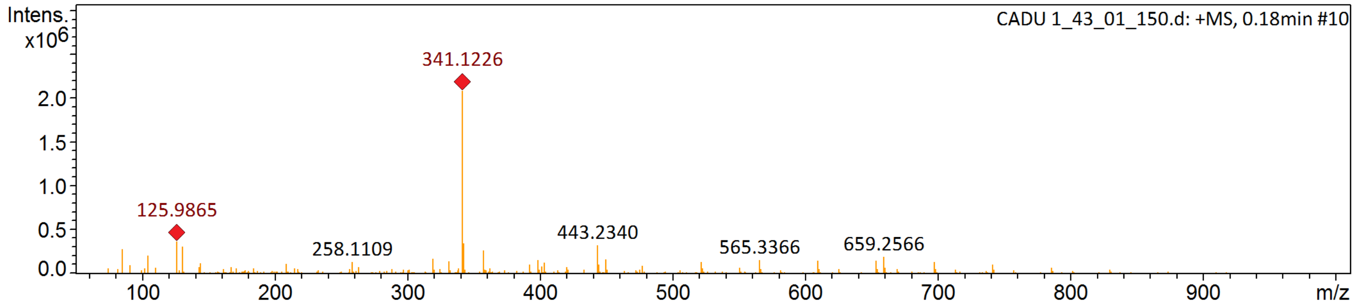

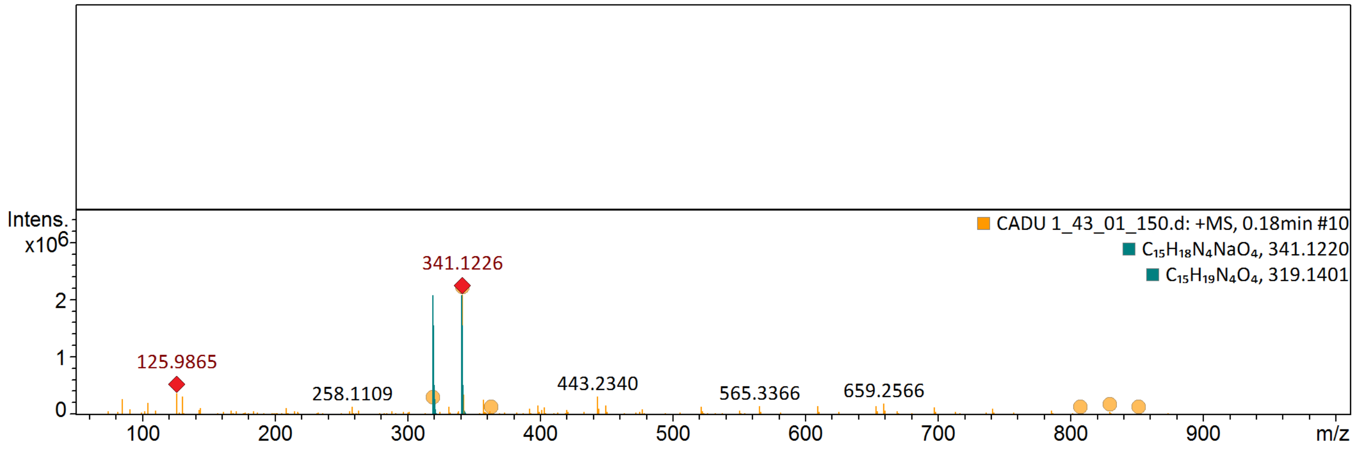

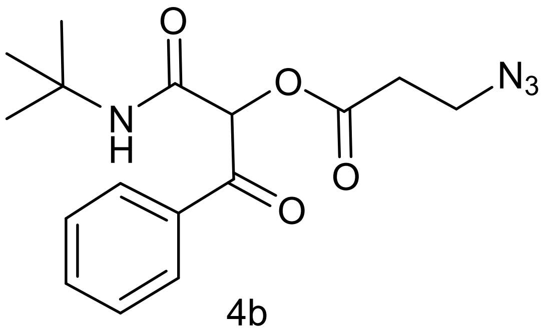

4b

Acquisition Parameter

Source Type	ESI	Ion Polarity	Positive	Set Nebulizer	2.0 Bar
Focus	Active	Set Capillary	4500 V	Set Dry Heater	180 °C
Scan Begin	50 m/z	Set End Plate Offset	-500 V	Set Dry Gas	9.0 l/min
Scan End	1000 m/z	Set Charging Voltage	2000 V	Set Divert Valve	Waste
		Set Corona	0 nA	Set APCI Heater	0 °C

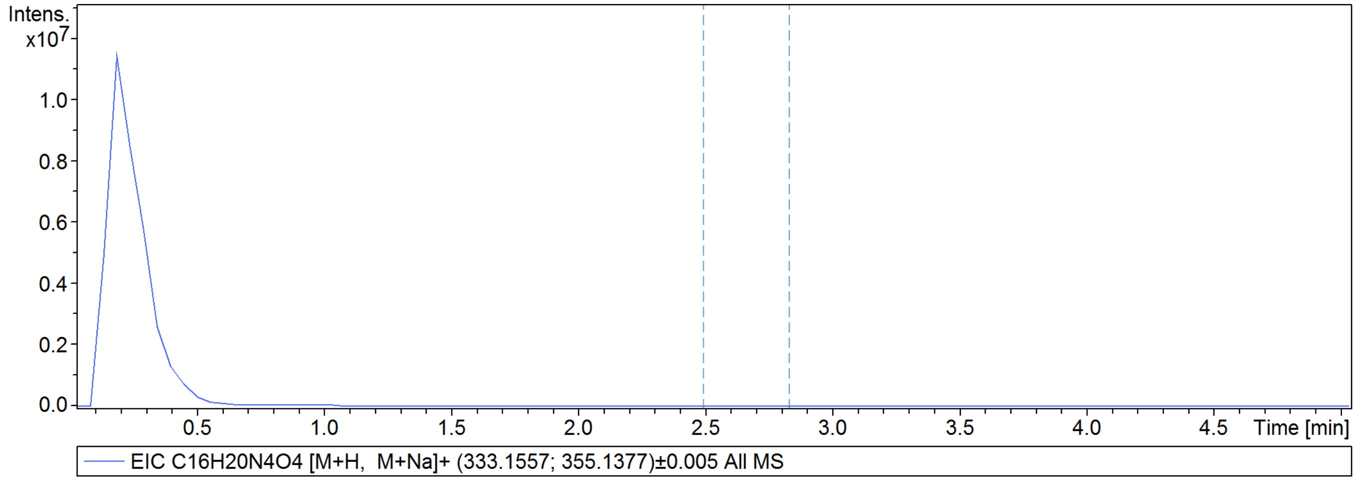

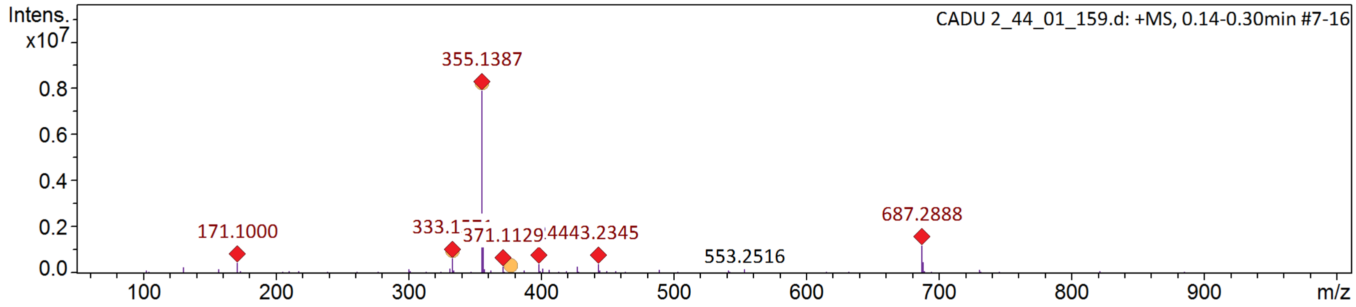

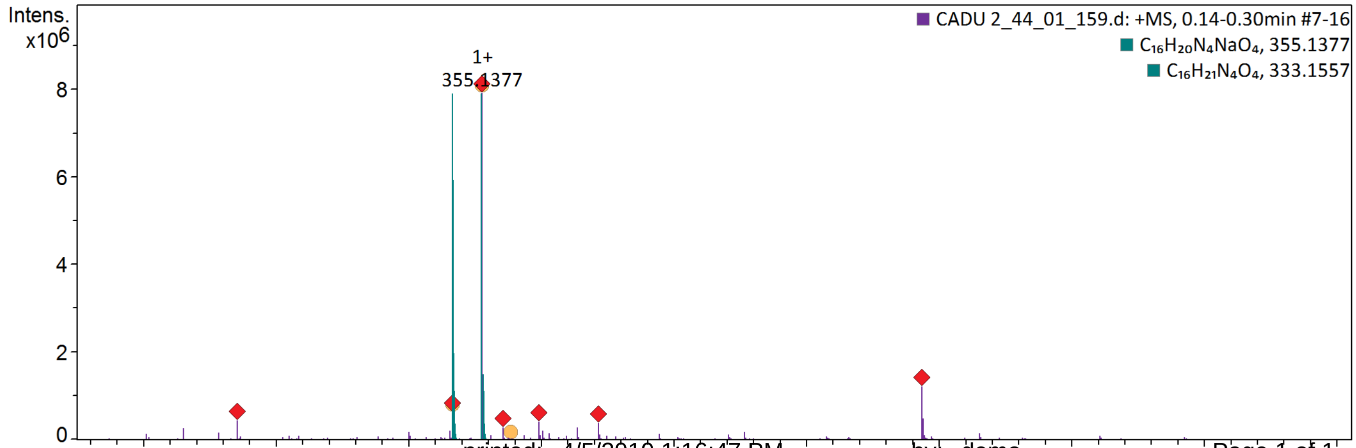

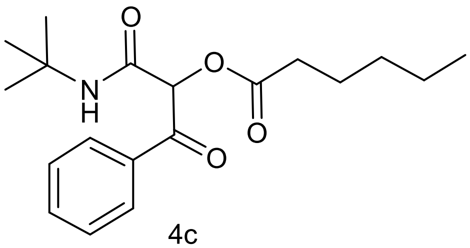

4c

Acquisition Parameter

Source Type	ESI	Ion Polarity	Positive	Set Nebulizer	2.0 Bar
Focus	Active	Set Capillary	4500 V	Set Dry Heater	180 °C
Scan Begin	50 m/z	Set End Plate Offset	-500 V	Set Dry Gas	9.0 l/min
Scan End	1000 m/z	Set Charging Voltage	2000 V	Set Divert Valve	Waste
		Set Corona	0 nA	Set APCI Heater	0 °C

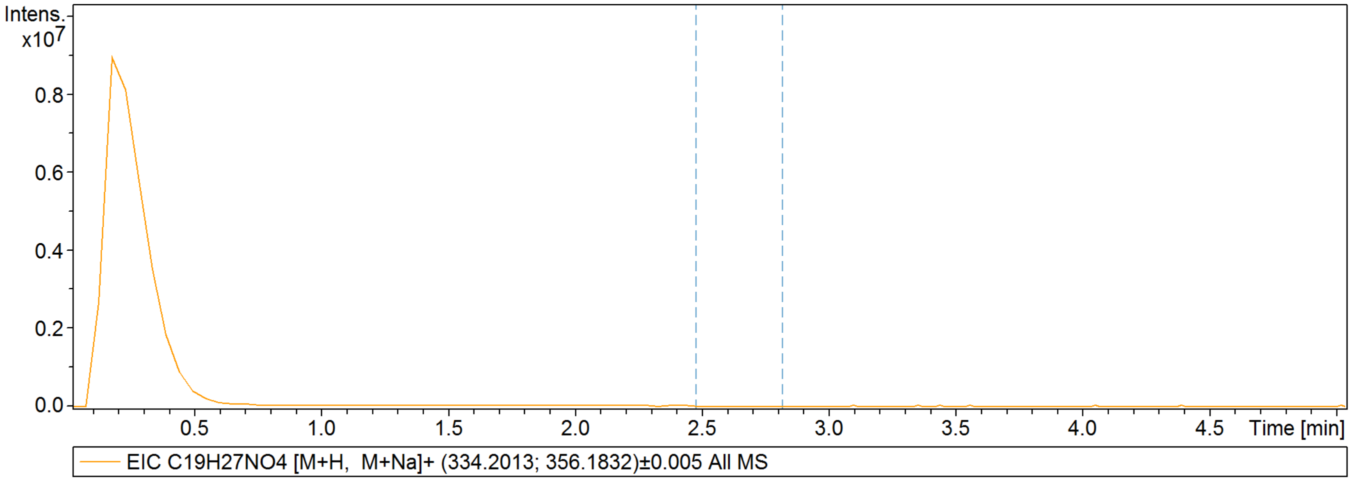

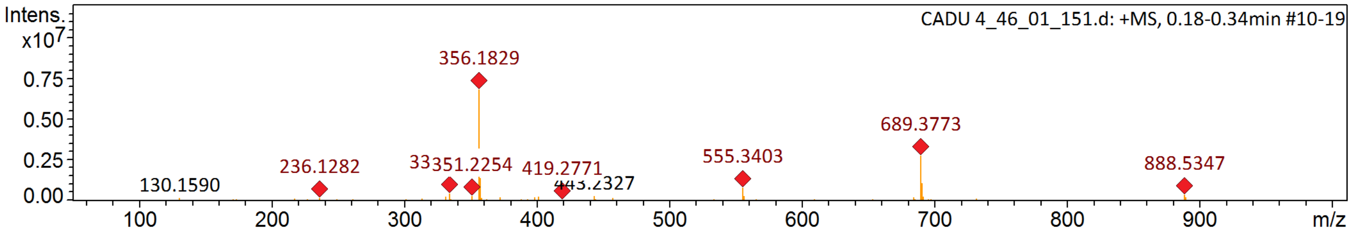

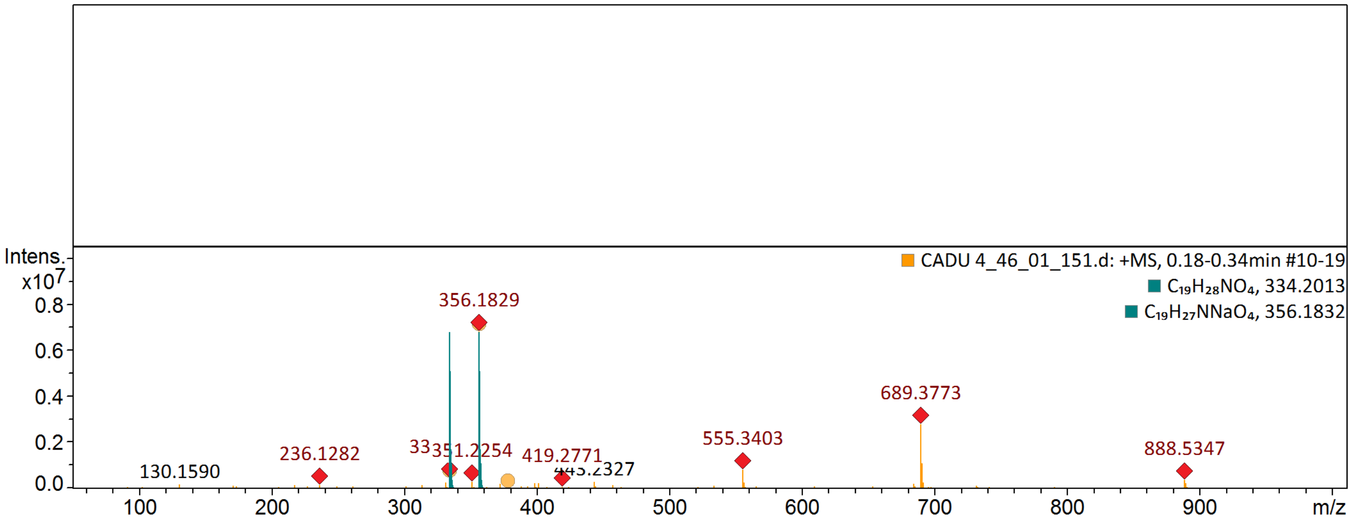

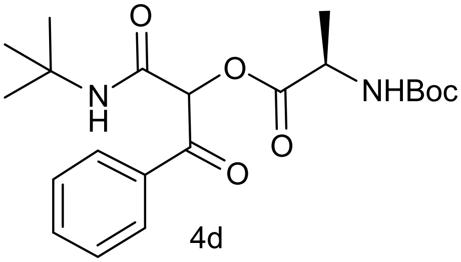

4d

Acquisition Parameter

Source Type	ESI	Ion Polarity	Positive	Set Nebulizer	2.0 Bar
Focus	Active	Set Capillary	4500 V	Set Dry Heater	180 °C
Scan Begin	50 m/z	Set End Plate Offset	-500 V	Set Dry Gas	9.0 l/min
Scan End	1000 m/z	Set Charging Voltage	2000 V	Set Divert Valve	Waste
		Set Corona	0 nA	Set APCI Heater	0 °C

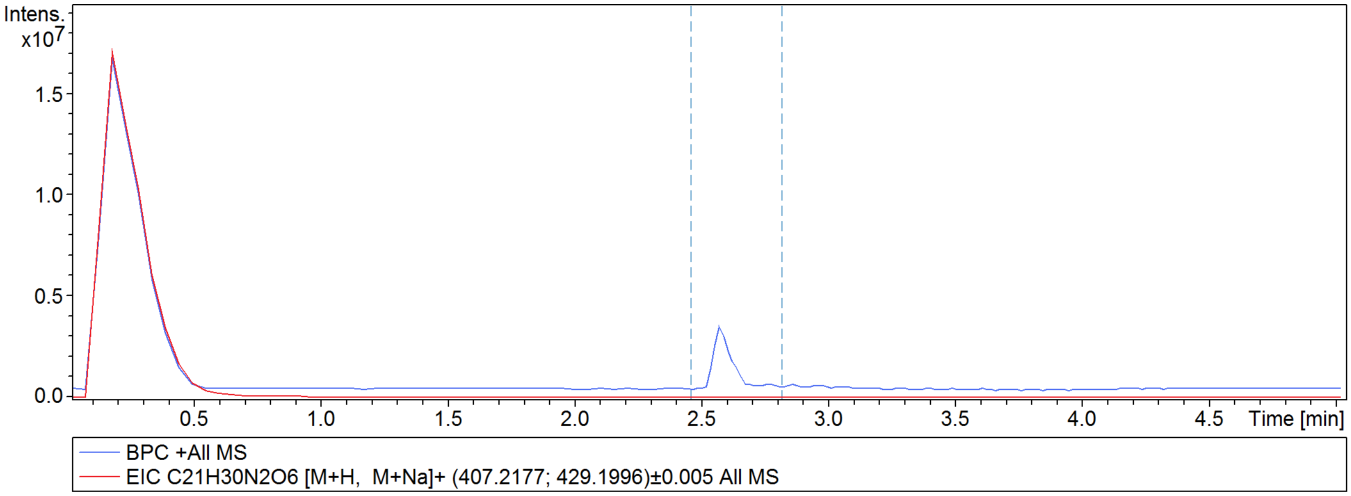

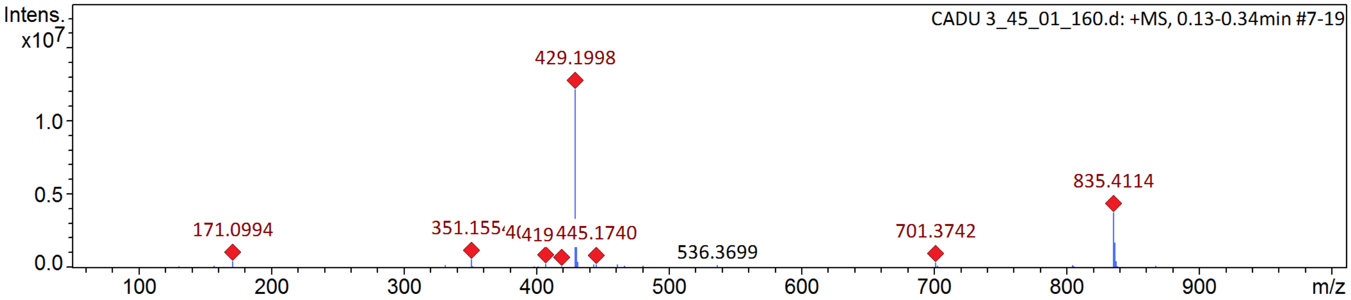

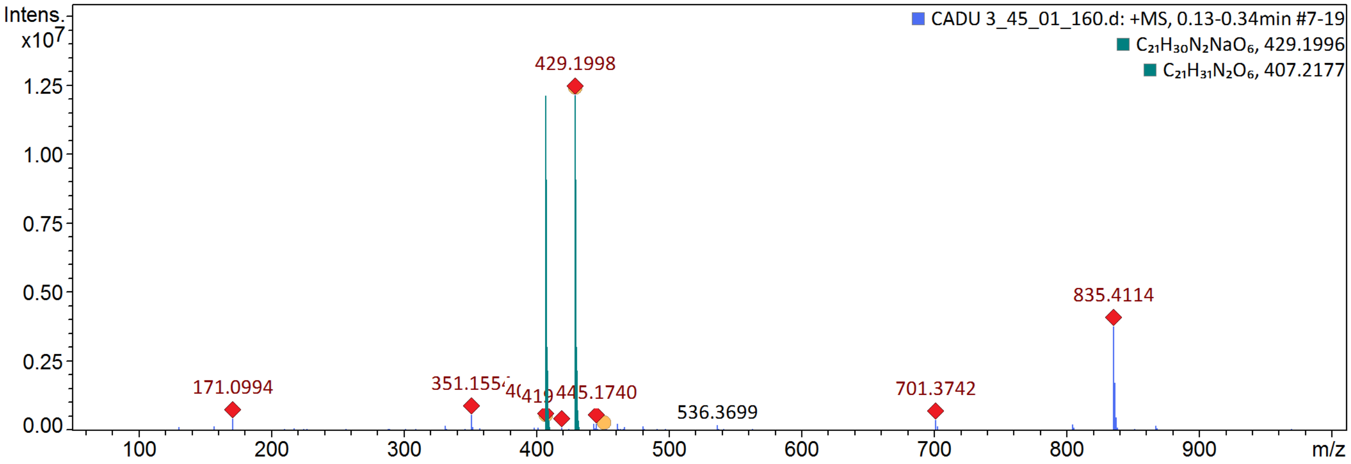

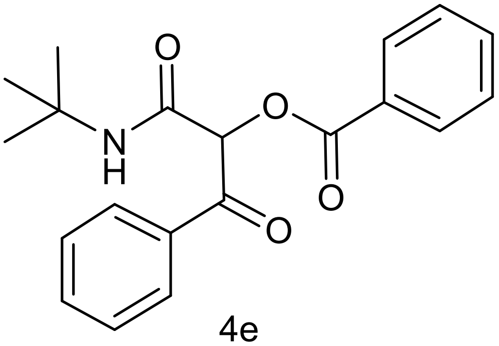

4e

Acquisition Parameter

Source Type	ESI	Ion Polarity	Positive	Set Nebulizer	2.0 Bar
Focus	Active	Set Capillary	4500 V	Set Dry Heater	180 °C
Scan Begin	50 m/z	Set End Plate Offset	-500 V	Set Dry Gas	9.0 l/min
Scan End	1000 m/z	Set Charging Voltage	2000 V	Set Divert Valve	Waste
		Set Corona	0 nA	Set APCI Heater	0 °C

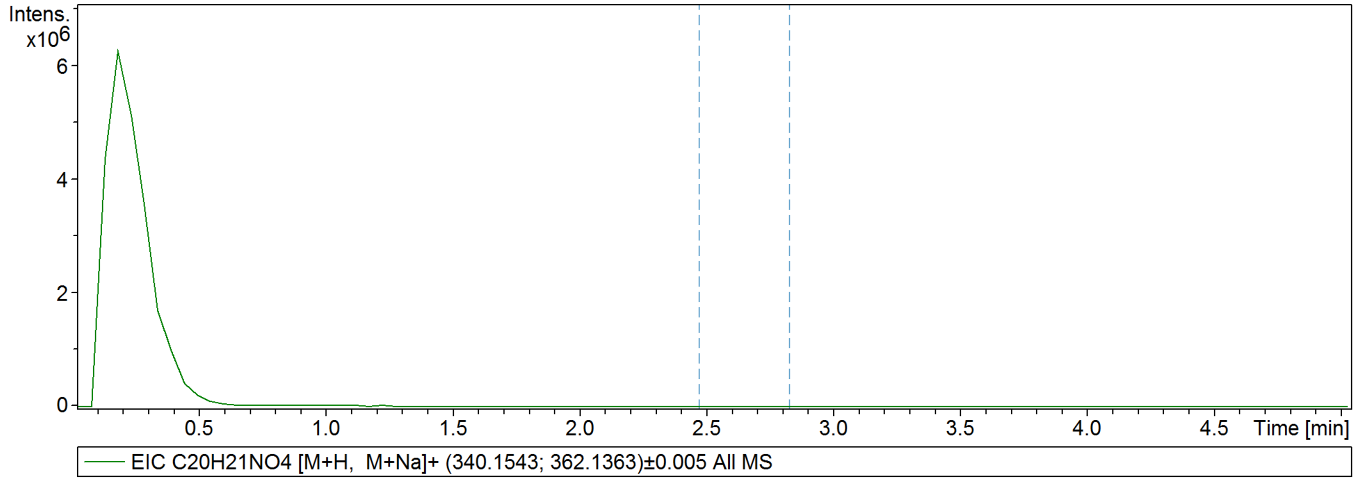

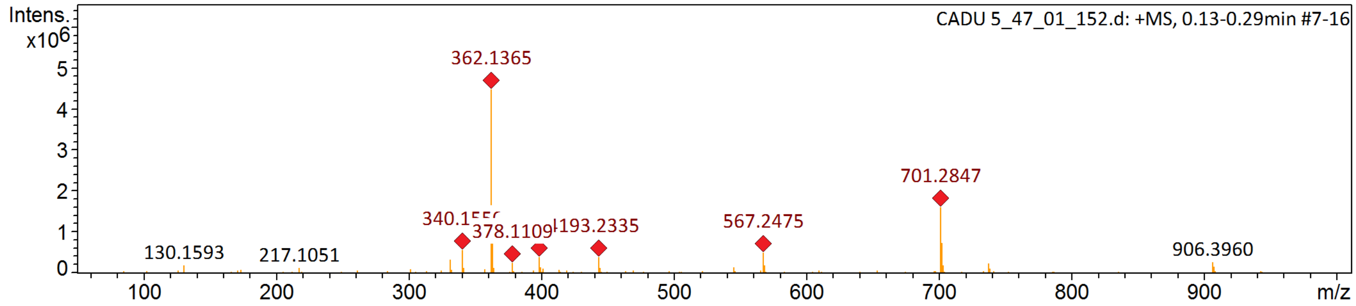

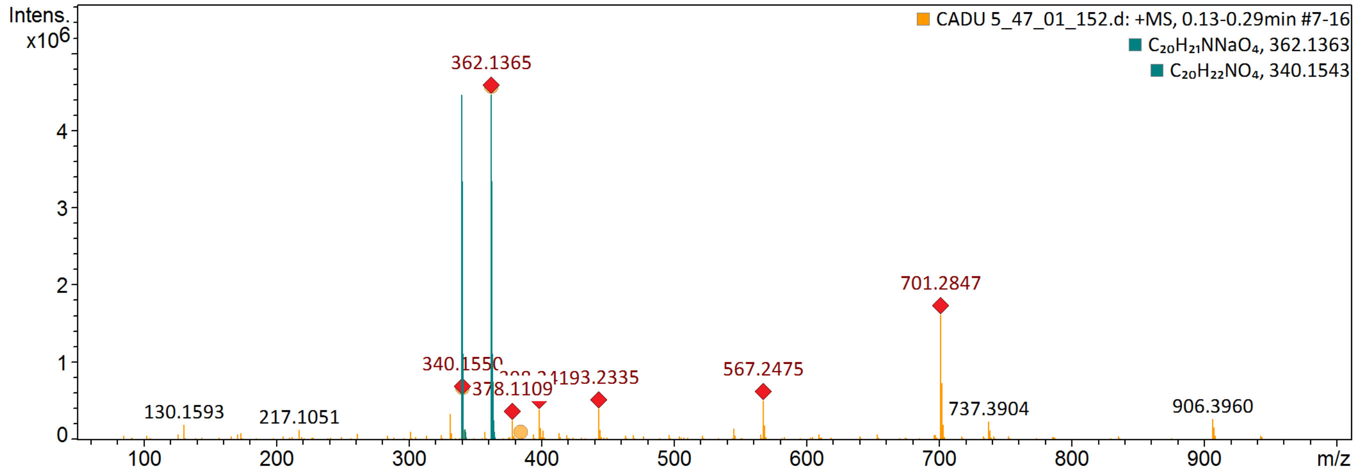

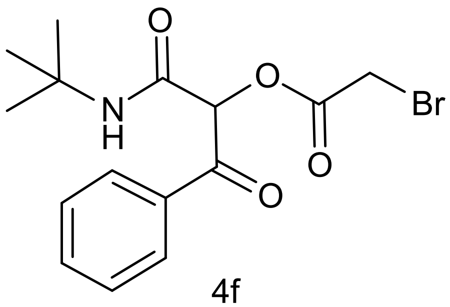

4f

Acquisition Parameter

Source Type	ESI	Ion Polarity	Positive
Focus	Active	Set Capillary	4500 V
Scan Begin	50 m/z	Set End Plate Offset	-500 V
Scan End	1000 m/z	Set Charging Voltage	2000 V
		Set Corona	0 nA

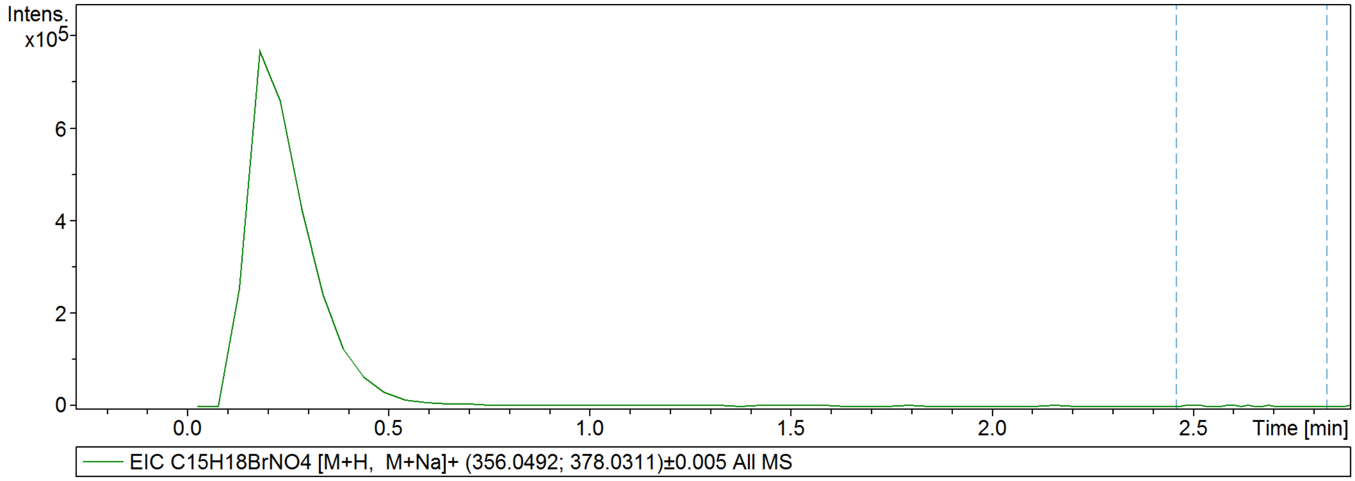

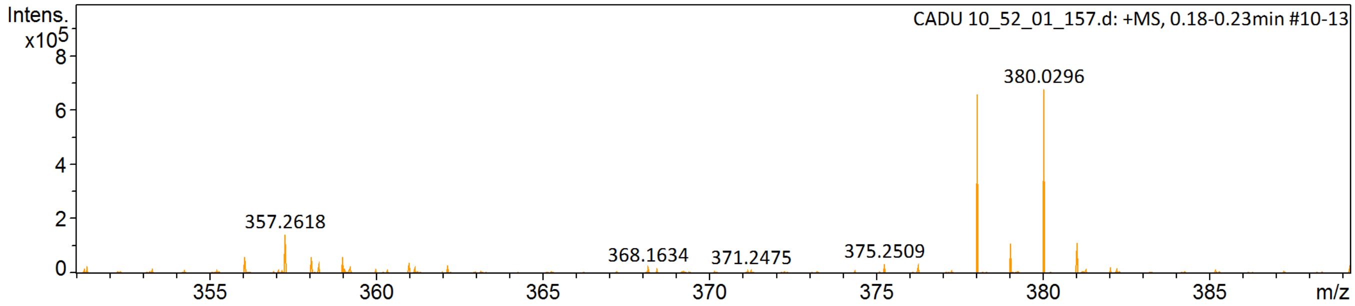

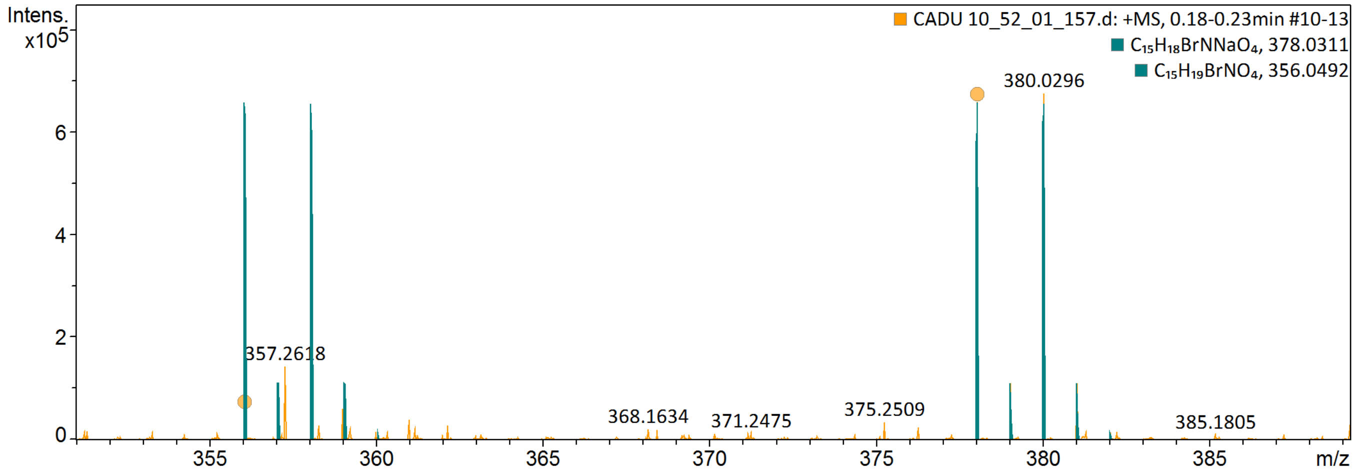

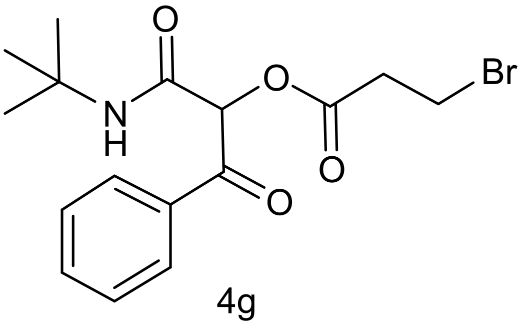

4g

Acquisition Parameter

Source Type	ESI	Ion Polarity	Positive	Set Nebulizer	2.0 Bar
Focus	Active	Set Capillary	4500 V	Set Dry Heater	180 °C
Scan Begin	50 m/z	Set End Plate Offset	-500 V	Set Dry Gas	9.0 l/min
Scan End	1000 m/z	Set Charging Voltage	2000 V	Set Divert Valve	Waste
		Set Corona	0 nA	Set APCI Heater	0 °C

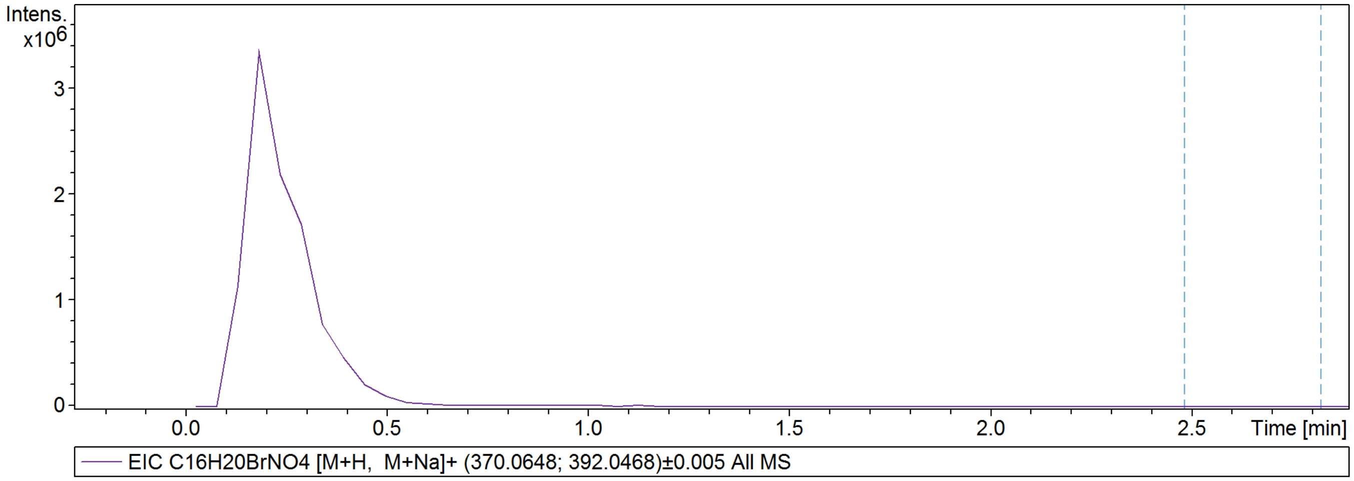

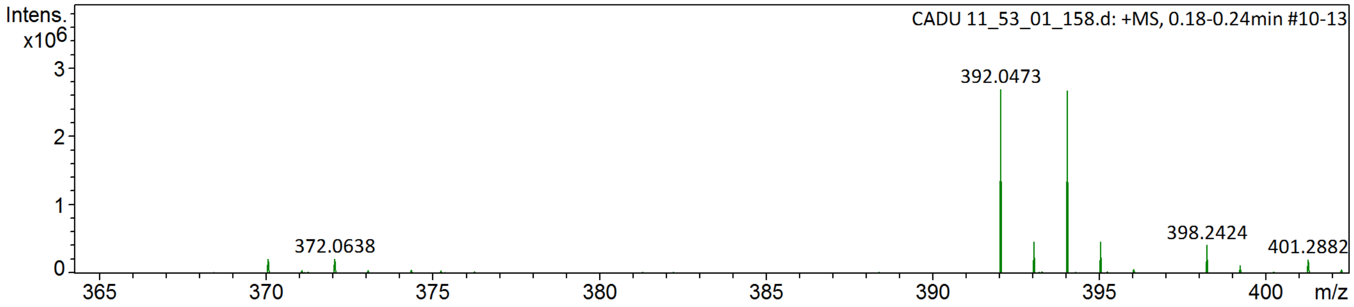

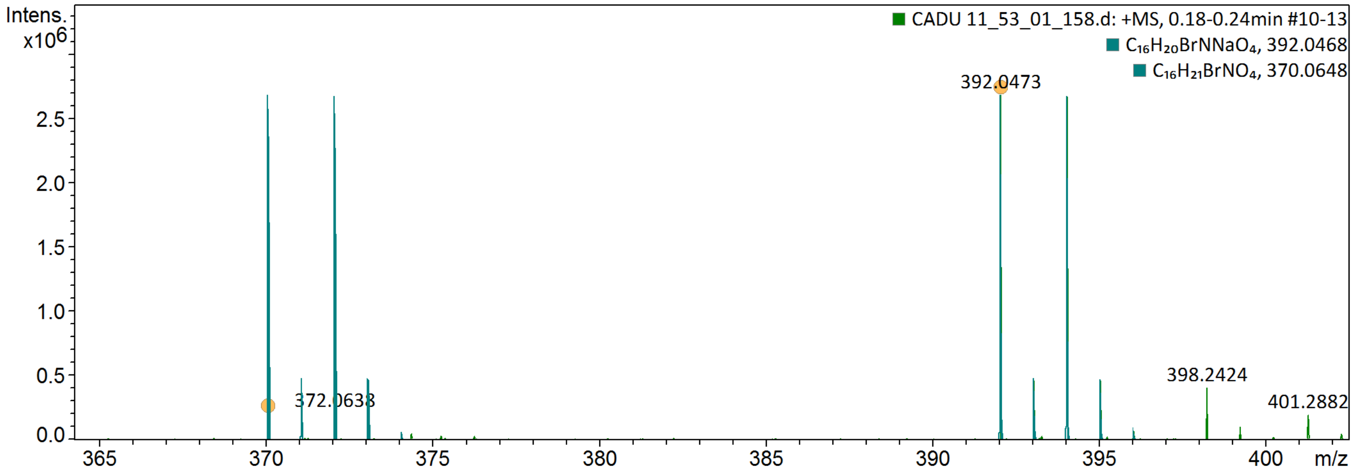

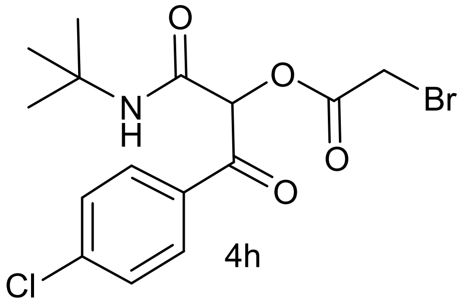

4h

Acquisition Parameter

Source Type	ESI	Ion Polarity	Positive	Set Nebulizer	2.0 Bar
Focus	Active	Set Capillary	4500 V	Set Dry Heater	180 °C
Scan Begin	50 m/z	Set End Plate Offset	-500 V	Set Dry Gas	9.0 l/min
Scan End	1000 m/z	Set Charging Voltage	2000 V	Set Divert Valve	Waste
		Set Corona	0 nA	Set APCI Heater	0 °C

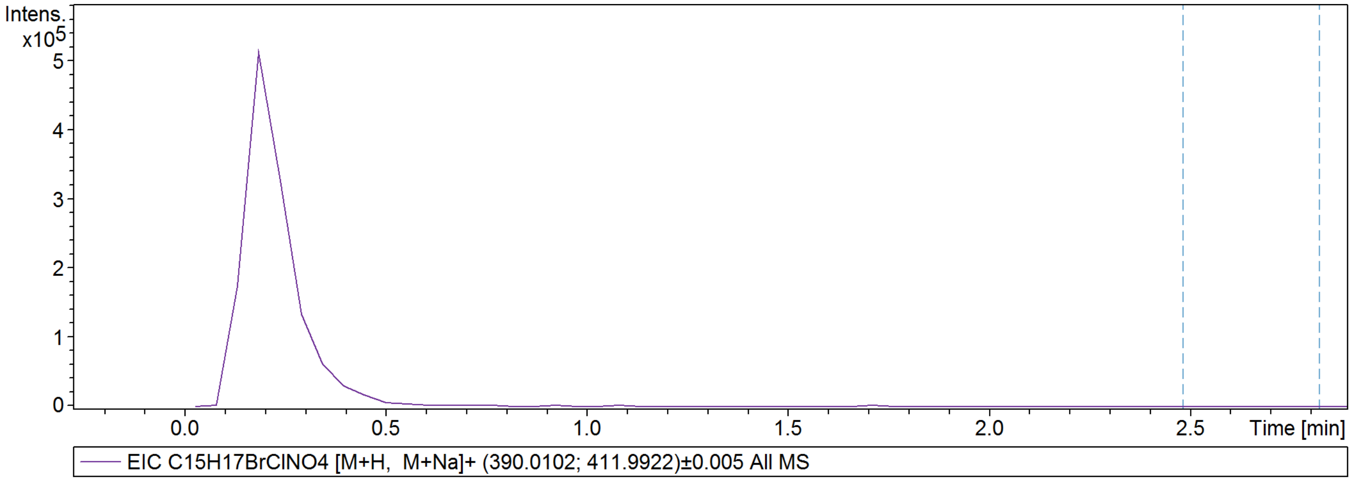

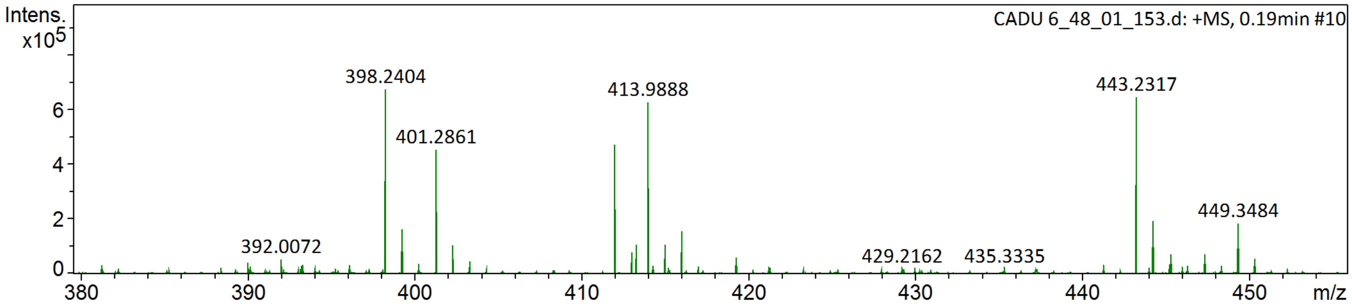

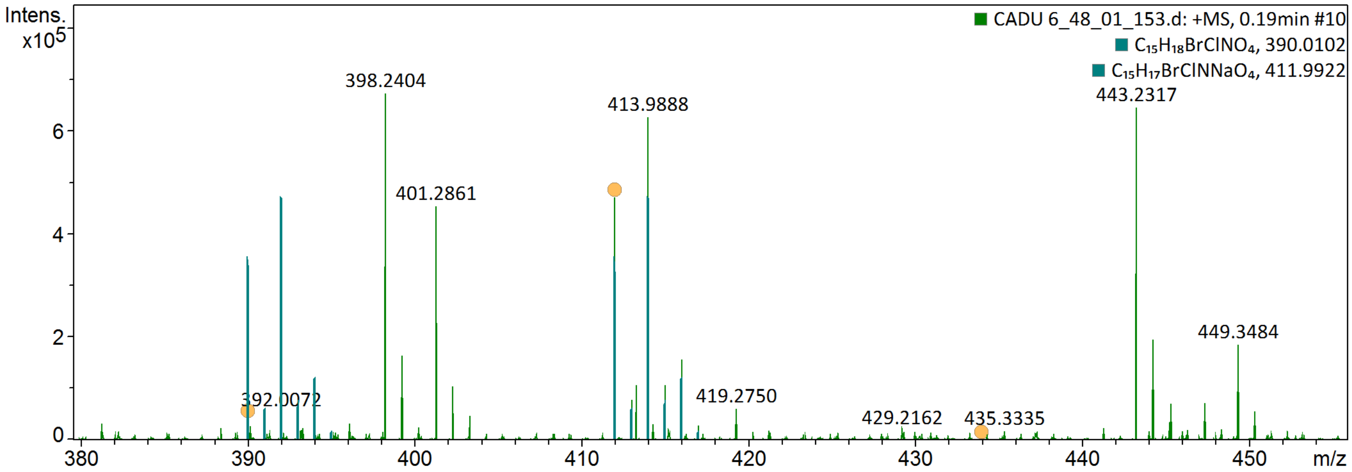

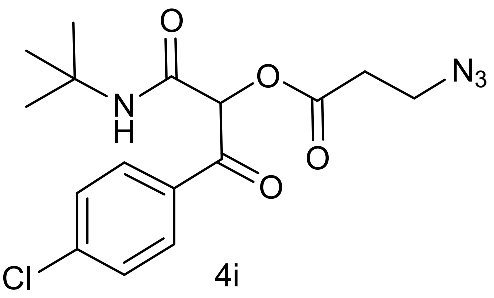

4i

Acquisition Parameter

Source Type	ESI	Ion Polarity	Positive	Set Nebulizer	2.0 Bar
Focus	Active	Set Capillary	4500 V	Set Dry Heater	180 °C
Scan Begin	50 m/z	Set End Plate Offset	-500 V	Set Dry Gas	9.0 l/min
Scan End	1000 m/z	Set Charging Voltage	2000 V	Set Divert Valve	Waste
		Set Corona	0 nA	Set APCI Heater	0 °C

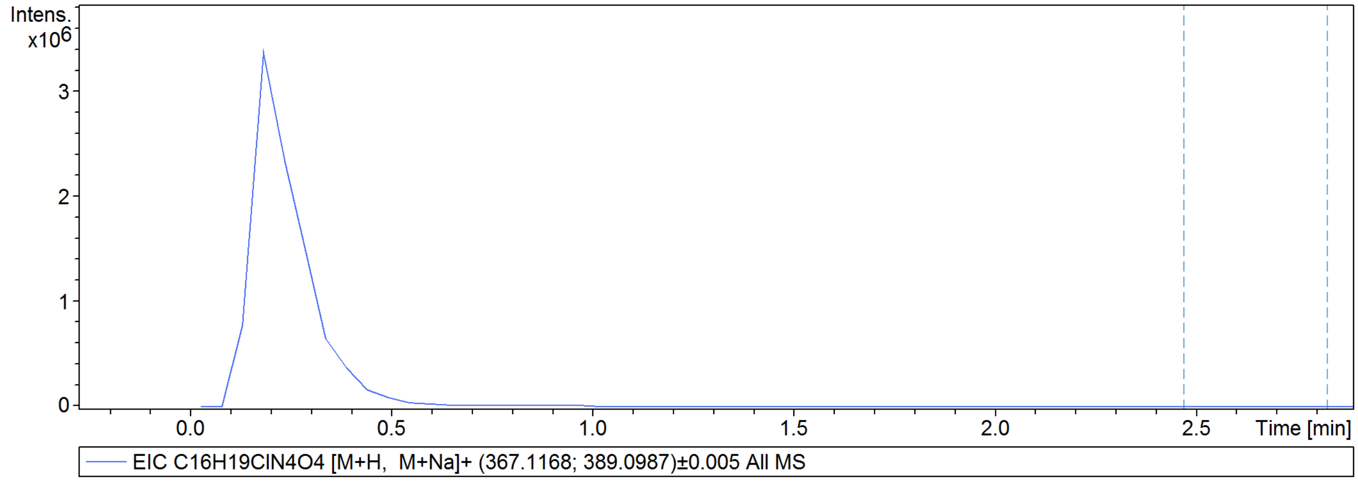

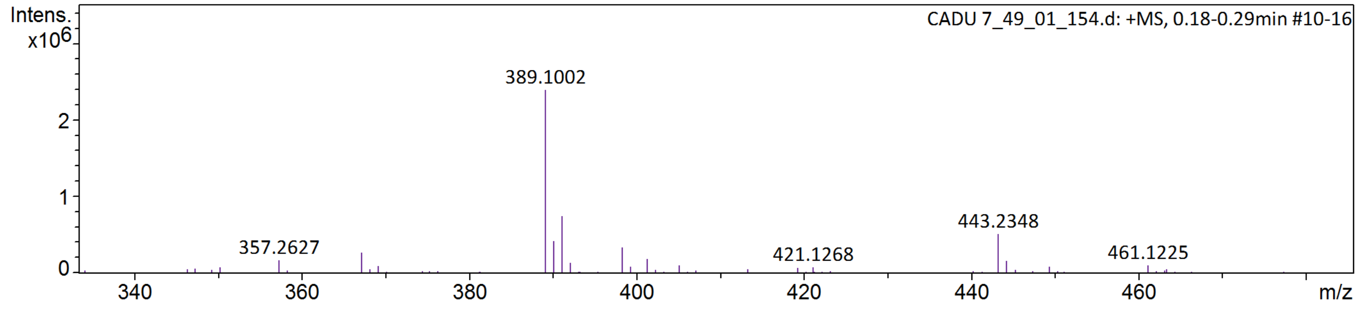

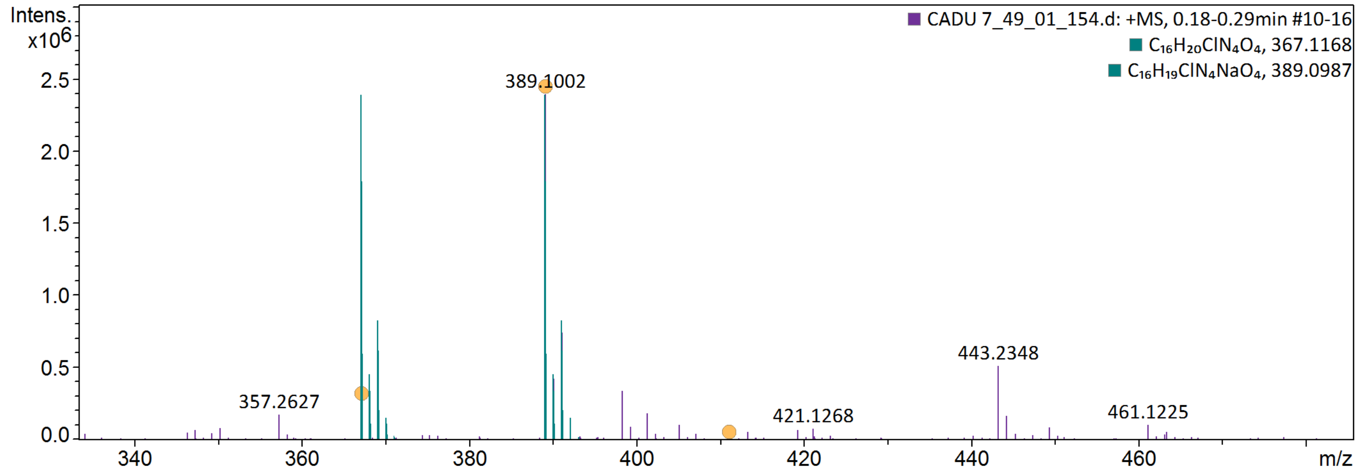

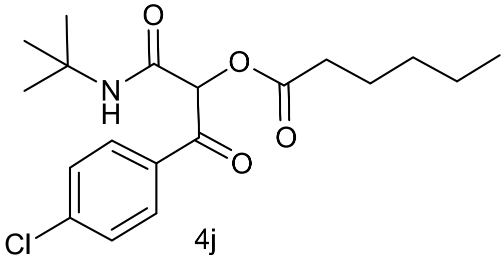

4j

Acquisition Parameter

Source Type	ESI	Ion Polarity	Positive	Set Nebulizer	2.0 Bar
Focus	Active	Set Capillary	4500 V	Set Dry Heater	180 °C
Scan Begin	50 m/z	Set End Plate Offset	-500 V	Set Dry Gas	9.0 l/min
Scan End	1000 m/z	Set Charging Voltage	2000 V	Set Divert Valve	Waste
		Set Corona	0 nA	Set APCI Heater	0 °C

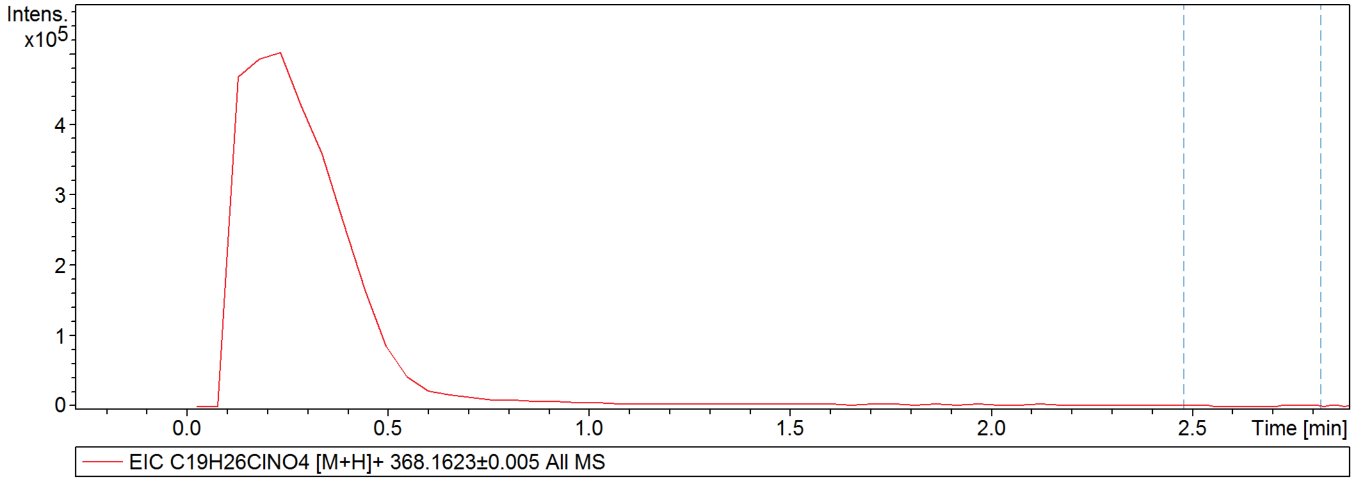

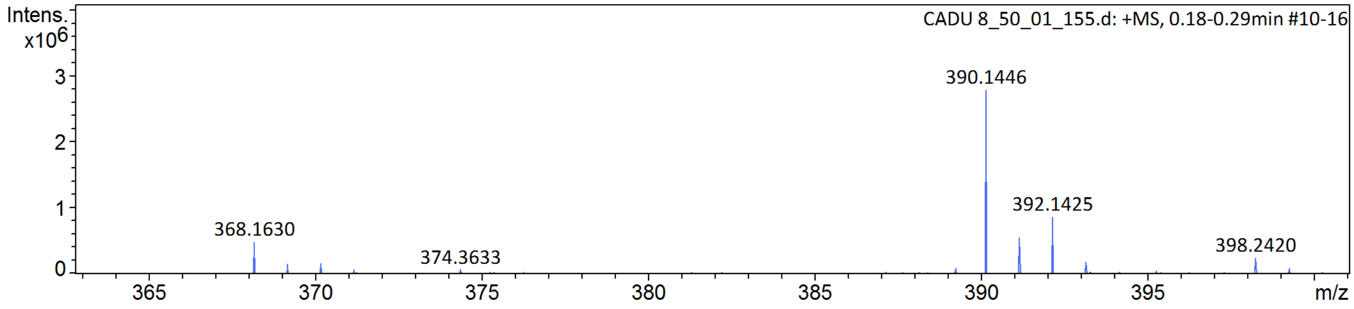

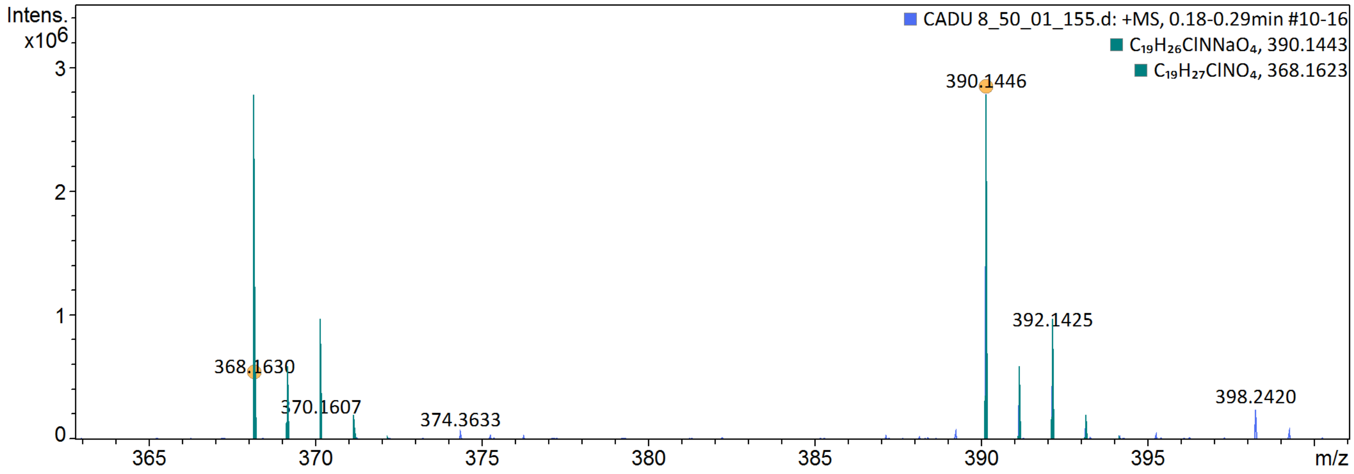

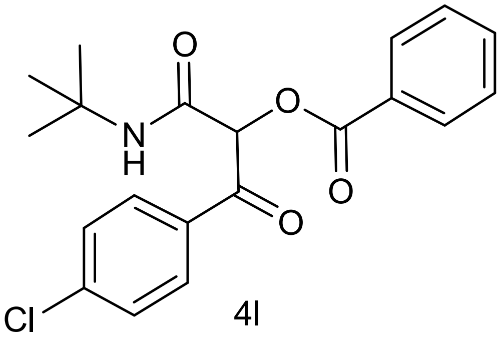

4l

Acquisition Parameter

Source Type	ESI	Ion Polarity	Positive	Set Nebulizer	2.0 Bar
Focus	Active	Set Capillary	4500 V	Set Dry Heater	180 °C
Scan Begin	50 m/z	Set End Plate Offset	-500 V	Set Dry Gas	9.0 l/min
Scan End	1000 m/z	Set Charging Voltage	2000 V	Set Divert Valve	Waste
		Set Corona	0 nA	Set APCI Heater	0 °C

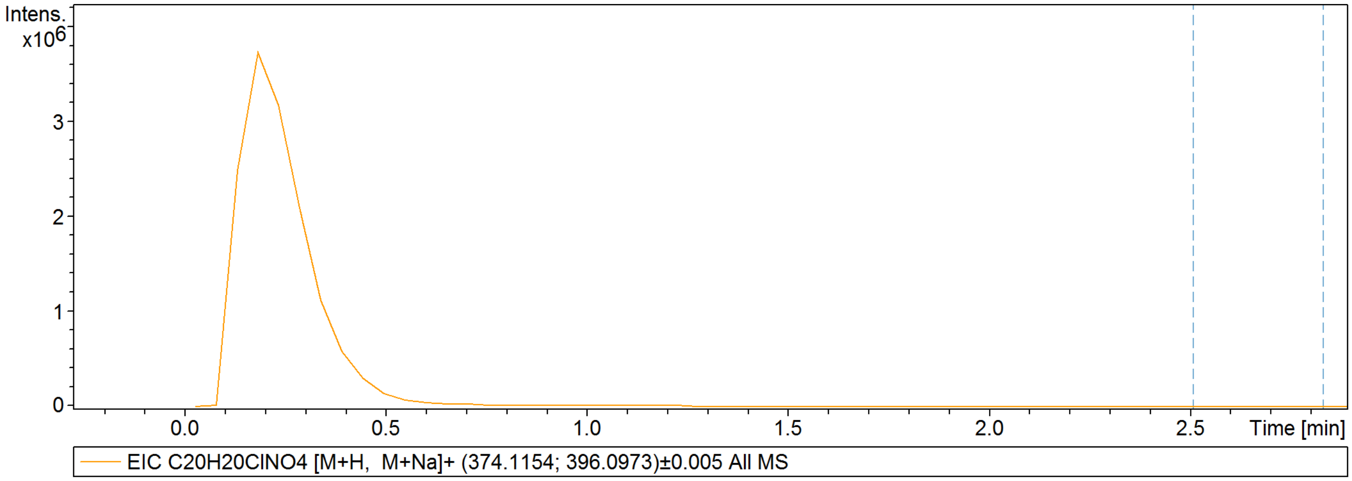

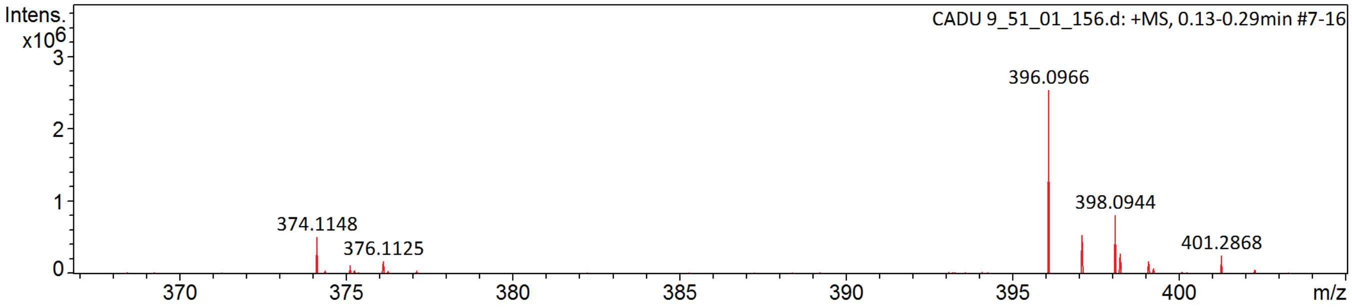

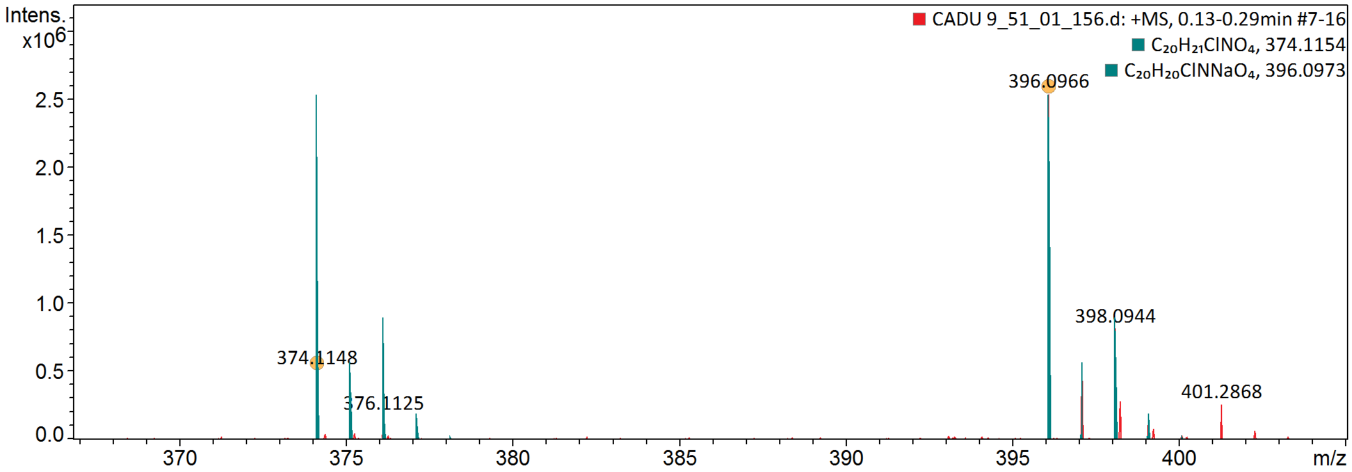

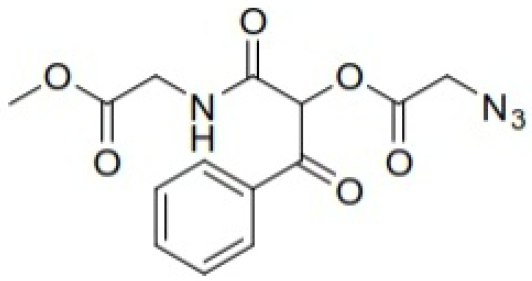

4m

Acquisition Parameter

Source Type	ESI	Ion Polarity	Positive	Set Nebulizer	2.0 Bar
Focus	Active	Set Capillary	4500 V	Set Dry Heater	180 °C
Scan Begin	50 m/z	Set End Plate Offset	-500 V	Set Dry Gas	9.0 l/min
Scan End	1000 m/z	Set Charging Voltage	2000 V	Set Divert Valve	Waste
		Set Corona	0 nA	Set APCI Heater	0 °C

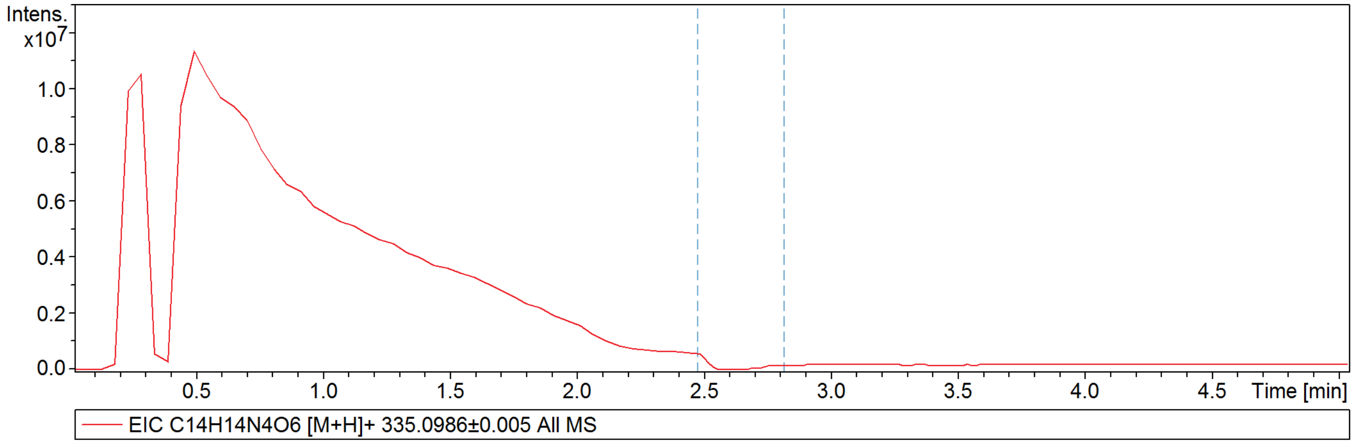

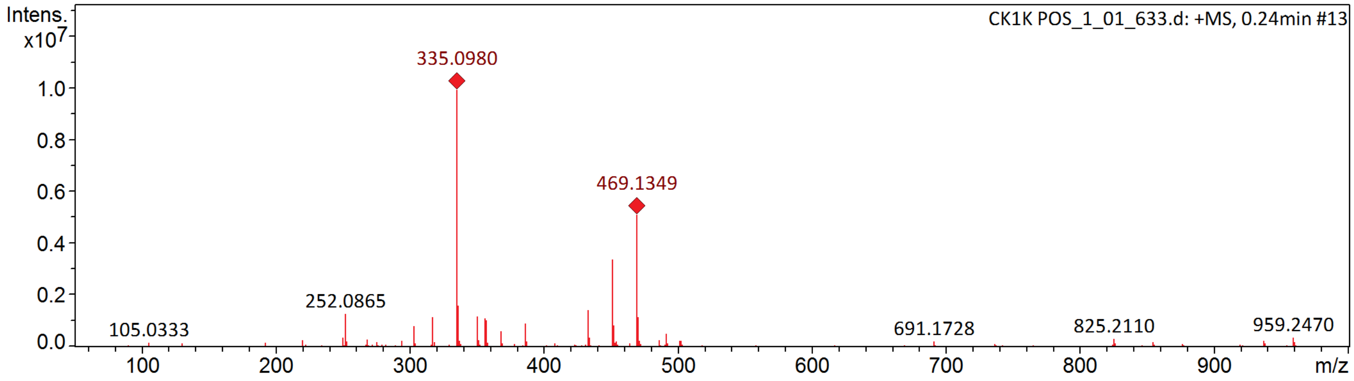

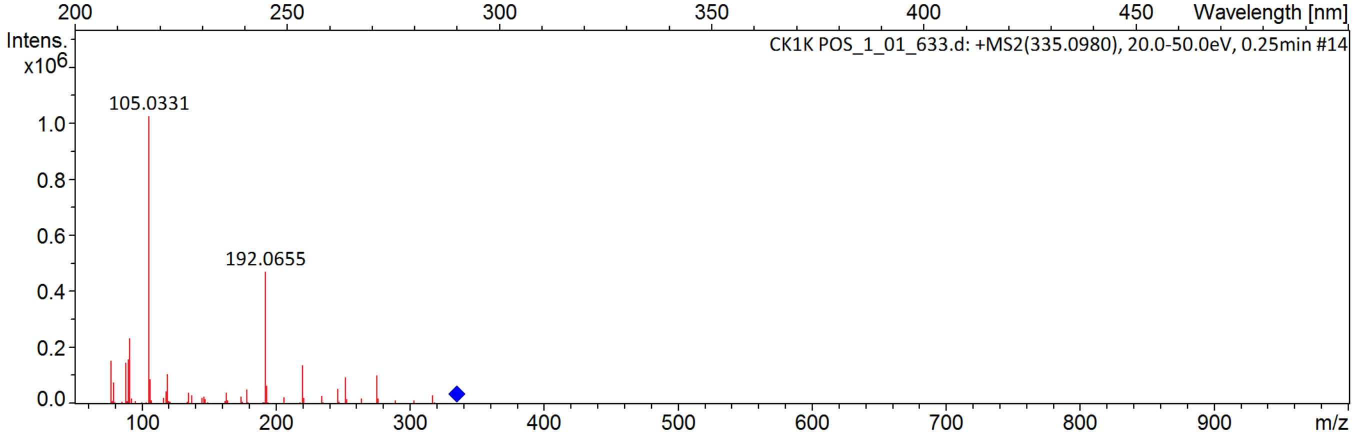

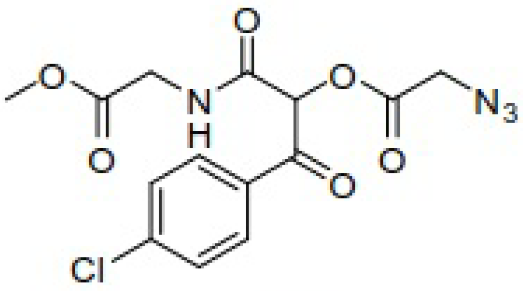

4n

Acquisition Parameter

Source Type	ESI	Ion Polarity	Positive	Set Nebulizer	2.0 Bar
Focus	Active	Set Capillary	4500 V	Set Dry Heater	180 °C
Scan Begin	50 m/z	Set End Plate Offset	-500 V	Set Dry Gas	9.0 l/min
Scan End	1000 m/z	Set Charging Voltage	2000 V	Set Divert Valve	Waste
		Set Corona	0 nA	Set APCI Heater	0 °C

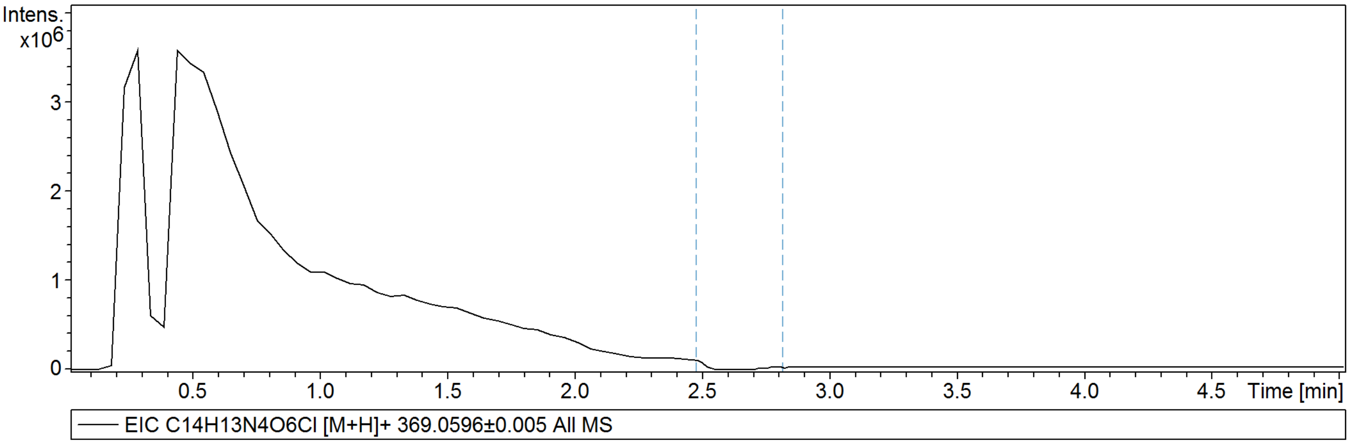

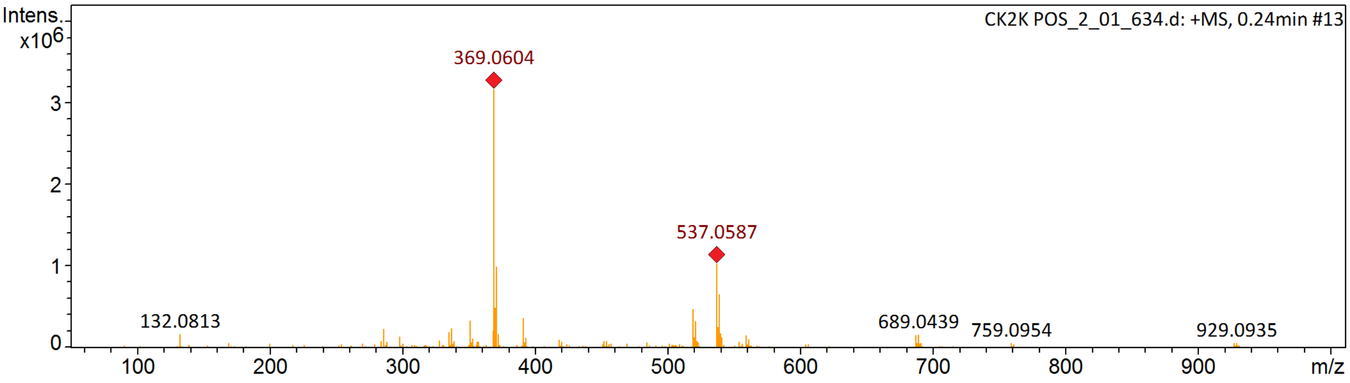

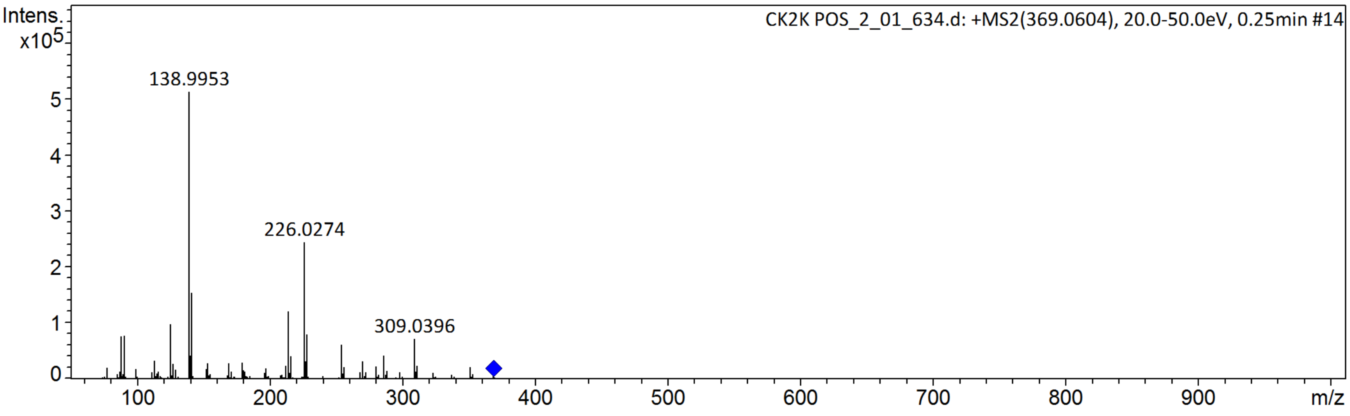

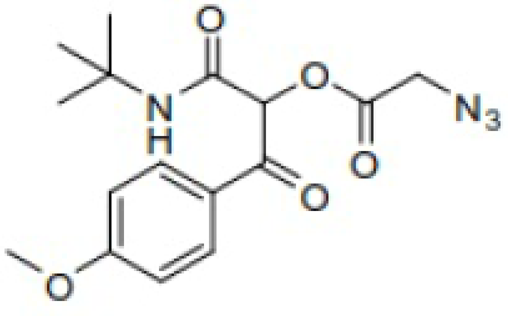

4o

Acquisition Parameter

Source Type	ESI	Ion Polarity	Positive	Set Nebulizer	2.0 Bar
Focus	Active	Set Capillary	4500 V	Set Dry Heater	180 °C
Scan Begin	50 m/z	Set End Plate Offset	-500 V	Set Dry Gas	9.0 l/min
Scan End	1000 m/z	Set Charging Voltage	2000 V	Set Divert Valve	Waste
		Set Corona	0 nA	Set APCI Heater	0 °C

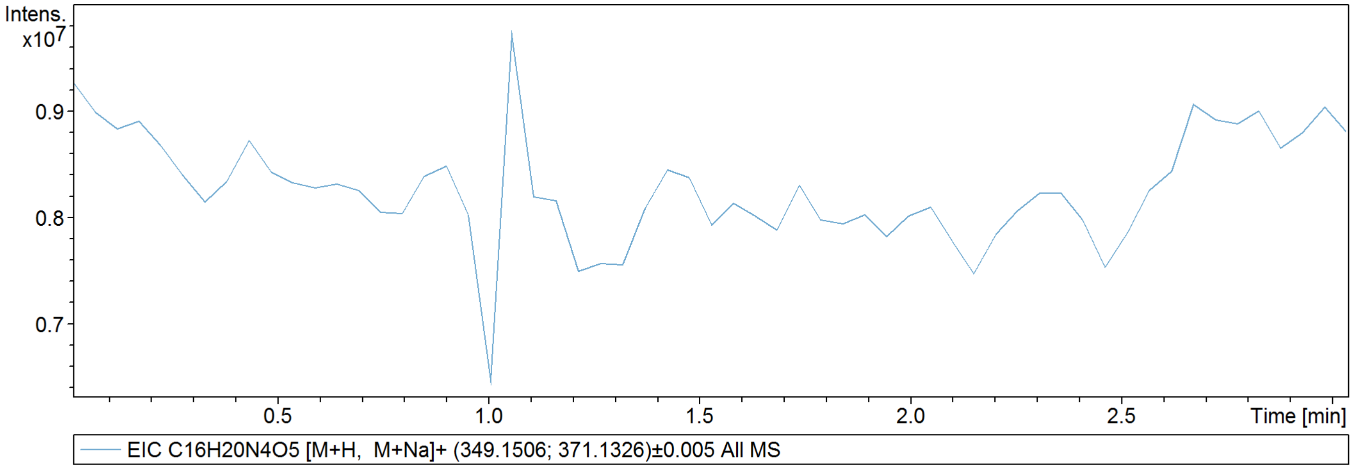

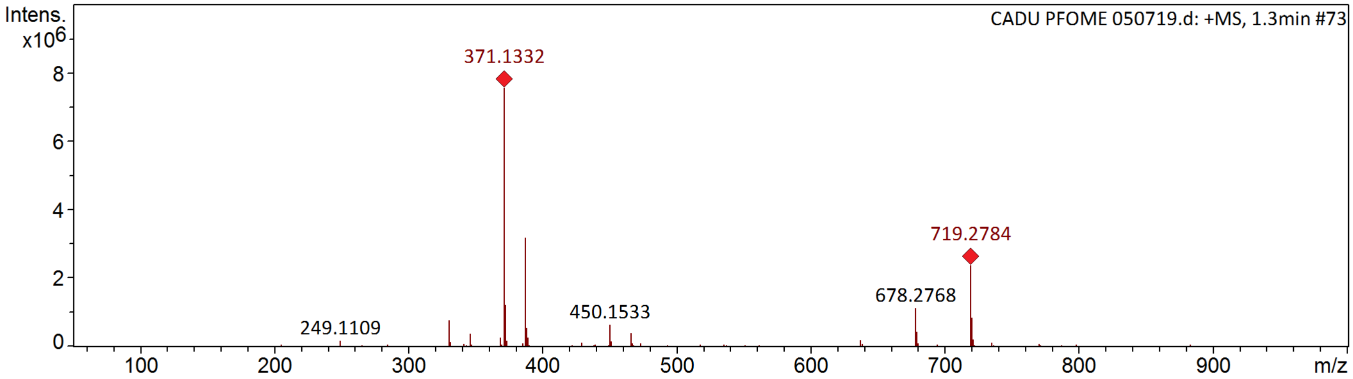

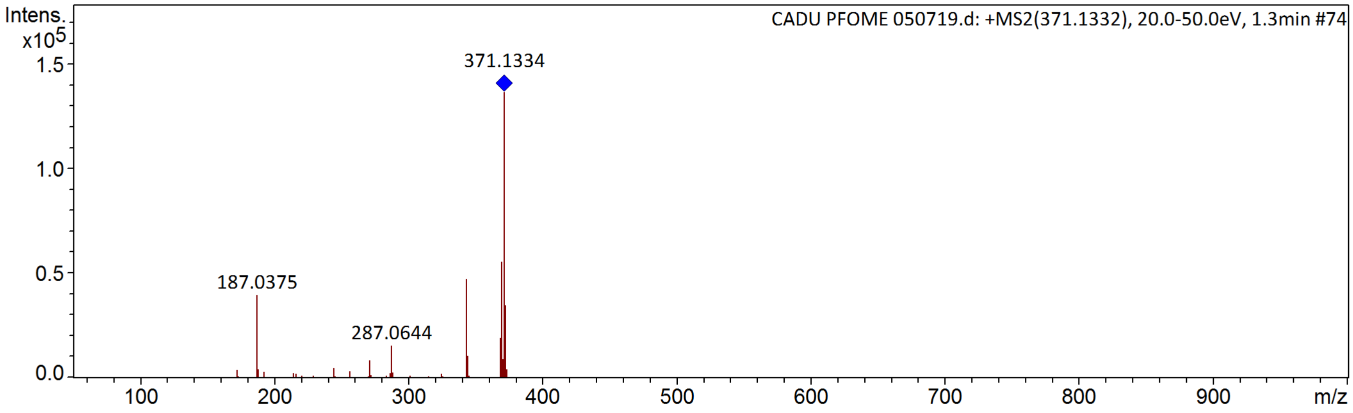

Supplement: Supplementary file 1 [file Data_Sheet_1.PDF]
